# Software for Computing and Annotating Genomic Ranges

**DOI:** 10.1371/journal.pcbi.1003118

**Published:** 2013-08-08

**Authors:** Michael Lawrence, Wolfgang Huber, Hervé Pagès, Patrick Aboyoun, Marc Carlson, Robert Gentleman, Martin T. Morgan, Vincent J. Carey

**Affiliations:** 1 Bioinformatics and Computational Biology, Genentech, Inc., South San Francisco, California, United States of America; 2 European Molecular Biology Laboratory Genome Biology Unit, Heidelberg, Germany; 3 The European Bioinformatics Institute, Cambridge, United Kingdom; 4 Computational Biology, Fred Hutchinson Cancer Research Center, Seattle, Washington, United States of America; 5 Channing Division of Network Medicine, Brigham and Women's Hospital, Harvard Medical School, Boston, Massachusetts, United States of America; University of California, San Diego, United States of America

## Abstract

We describe Bioconductor infrastructure for representing and computing on annotated genomic ranges and integrating genomic data with the statistical computing features of R and its extensions. At the core of the infrastructure are three packages: *IRanges*, *GenomicRanges*, and *GenomicFeatures*. These packages provide scalable data structures for representing annotated ranges on the genome, with special support for transcript structures, read alignments and coverage vectors. Computational facilities include efficient algorithms for overlap and nearest neighbor detection, coverage calculation and other range operations. This infrastructure directly supports more than 80 other Bioconductor packages, including those for sequence analysis, differential expression analysis and visualization.

This is a *PLOS Computational Biology* Software Article.

## Introduction

The genome is typically represented as a linear sequence, split over multiple chromosomes, and data are linked to the genome by occupying a range of positions on the sequence. These data fall into two broad categories. First, there are the annotations, such as gene models, transcription factor binding site predictions, GC percentage, polymorphisms, and conservation scores. Such annotations are highly processed and are often served by public databases such as NCBI or EBI. Second, there are primary experimental measurements, such as read alignments from high-throughput sequencing. Data integration, within and between those two categories, is made possible by treating the data as ranges on the genome, which acts as a common scaffold. Thus, ranges play a central role in genomic data analysis, and statistical tools should consider ranges to be as fundamental as quantitative and categorical data types.

For example, ranges are integral to the manipulation of gene model annotations. Examples include deriving candidate promoter regions, finding introns, calculating the total exonic length of a transcript or finding the exonic regions that are unique to a particular transcript in an alternatively spliced gene. Ranges also play a central role in the analysis of experimental data, where they are used to represent read alignments. In the analysis of ChIP-seq data, it is typical to calculate the depth of alignment coverage, which then serves as input to calling algorithms which output peaks as ranges. These ranges are then annotated according to their overlap with and proximity to other ranges, such as gene structures. Similarly, for RNA-seq data, analysts measure gene expression based on counting the alignments overlapping exons.

All these analyses depend on specialized, range-based algorithms and data structures. For example, computations on gene models involve set operations on ranges, including intersection, union and complement. Coverage calculation is important for detecting regions of enrichment and for producing visual summaries. Overlap and nearest neighbor detection is fundamental to the annotation of ChIP-seq peaks, estimating expression from RNA-seq data and many other integrative analyses.

The primary argument for storing ranges in specialized, formal data structures is efficiency, in terms of both implementation and language. The notion of ranges can be made explicit in the application programming interface (API), permitting the expression of algorithms in a succinct and readable language that illustrates concepts instead of exposing implementation details. Another goal is interoperability: by using the same data structures, multiple routines, spread across different packages, can operate on the data without cumbersome conversions. Also, a data structure can be accessed through an abstraction that hides the details of the optimized implementation, and this results in looser coupling between components. Together, these benefits lead to more robust, maintainable software.

Data structures should support the storage of per-range metadata, because genomic data is multivariate and consists of much more than the ranges alone. This enables the storage of gene identifiers and other symbols with the gene ranges, and the peak heights or confidence scores with the peak ranges. Some metadata merit special treatment, such as the chromosome name and the strand. Also necessary is a data structure for storing summaries and processing results for a common set of ranges across multiple samples. Such a structure would hold, for example, the RNA-seq per-exon counts or a set of variant calls. Finally, there should be support for storing hierarchies of ranges, at least for one level of nesting, to represent, for example, the nesting of exons into transcripts. Whether it is appropriate to treat the exons as individual ranges or the transcript as a compound range depends on the use case; both should be supported.

These data structures are represented as classes, through which we communicate the formal definition of each data structure to the programming language. One benefit is that we can defer the regulation of data access and the tracking of data integrity to the language. In the case of functional object-oriented languages, there is another benefit: we can implement behaviors as methods on generic functions. A generic function is one that dispatches to a particular implementation, termed a method, based on the classes of passed arguments. This means that the same API will exhibit specialized behavior depending on the input. For example calling start on a range data structure would return the starting positions for the ranges, while calling the same function on a base R time-series object would behave differently.

This paper describes the infrastructure in Bioconductor [1] for the integrative statistical analysis of range-based genomic data. Main features include scalable data structures for annotated genomic ranges and genome-length vectors, and efficient algorithms for overlap detection and other range operations. The packages that form the core of the infrastructure include *IRanges*, *GenomicRanges* and *GenomicFeatures*. Source code for the packages is included in the supplement, under Software S1, S2, and S3, respectively. The *IRanges* package provides the fundamental range data structures and operations, while *GenomicRanges* builds upon it to add biological semantics to the metadata, including explicit treatment of sequence name and strand. Finally, *GenomicFeatures* enables access to and manipulation of gene models and other annotations. Together, these packages support more than 80 other packages in Bioconductor.

Other software tools provide facilities for working with genomic ranges, e.g., *bedtools* [2] and *cisGenome* [3]. Those provide UNIX command-line interfaces and rely on common file formats (which are often incompletely specified) to interoperate with other tools, leading to workflows embodied as: collections of heterogeneous scripts, system dependencies and data files. Such workflows can be difficult to maintain and challenging to reproduce. In contrast, the Bioconductor infrastructure is tightly integrated with other R packages through in-memory data structures, while still supporting interaction with external tools. The Bioconductor package *genomeIntervals* provides data structures for representing genomic ranges and utilities, such as overlap detection, that have much in common with the tools described here, but our tools are more extensive and have been more widely adopted.

## Design and Implementation

### Working with Simple Ranges

**Figure 1:**
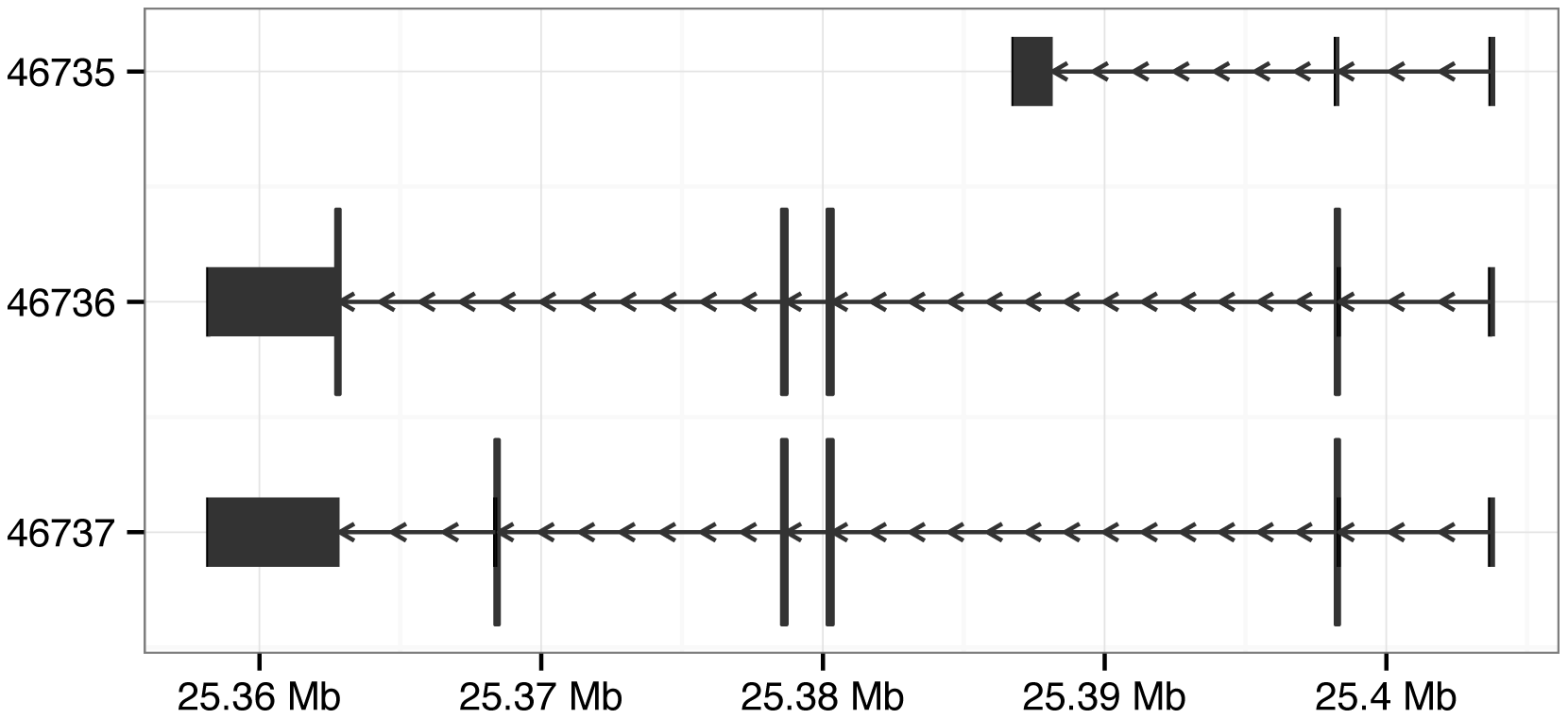
Tabular (top) and visual (bottom) representation of the exons for the human *KRAS* gene, derived from the UCSC known gene annotation. In the table, the columns *seqnames*, *start* and *end* locate the exons in the genome. The *strand* column indicates the direction of transcription. The exons are grouped into transcripts by *tx_id*, and the exon IDs are given by *exon_id*. Virtually all genomic data sets fit this pattern: genomic location, followed by a series of columns, often including strand and/or score, that annotate that location. In the plot, the rectangles represent exonic regions, and the arrows represent the introns, as well as the strand.

We use the term “range” to denote an ordered set of consecutive integers. A range is represented by a pair of integers 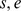 satisfying 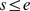. In Figure 1, 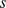 and 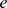 correspond to the *start* and *end* columns, respectively. The “width” of a range is given by 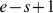, so a range for a single integer (modeling, for example, a single nucleotide position) has 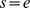.

The *IRanges* package, which is designed to be general and thus avoids biology-specific considerations, introduces the *IRanges* class to represent a vector of ranges. The *GenomicRanges* package builds on *IRanges* to include biologically relevant features such as strand and sequence (e.g., chromosome) name.

In Figure 1, we show a table of the exons of the human gene *KRAS*. The *tx_id* column indicates the transcripts to which each exon belongs. A single *IRanges* object can store those exon ranges, and this model is appropriate for per-exon analyses.

The *IRanges* class supports the basic R vector API, including the length accessor, extraction and subsetting functions like [[ and [ , concatenation with c, etc. This will hold true for all vector-like objects in the range infrastructure.

**Figure 2:**
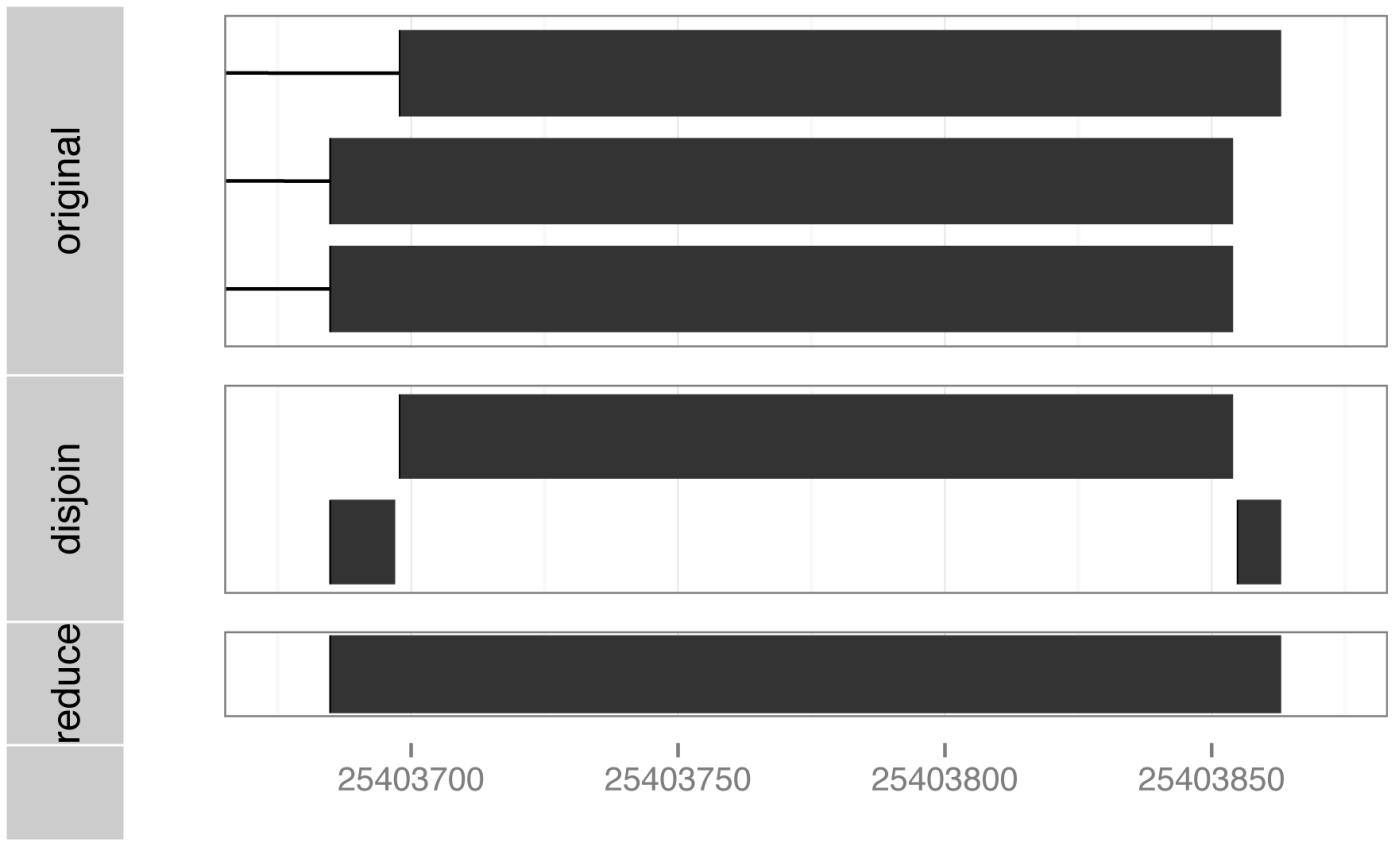
Illustration of the reduce and disjoin operations on the last exon from each of the *KRAS* transcripts.

The available range operations are listed in Table 1. The *IRanges* object supports direct manipulation of the start, end and width of the contained ranges. In applications, many of these operations follow recurrent patterns, and manipulating start and end directly can be needlessly tedious and error-prone. For this reason, *shift*, *resize* and similar frequently useful range operations are provided. Ranges can be simplified and summarized with several functions, including range, reduce and disjoin. Figure 2 illustrates the latter two. It is often appropriate to conceive of an *IRanges* object as a mathematical set of integers, or, in the biological context, a set of nucleotide positions. gaps (complement), union, intersect and setdiff support this notion. For example, taking the union of two transcripts would yield the ranges covered by any *KRAS* exon. The flank function could be used to demarcate putative promoter regions of transcripts.

**Table 1:** Summary of the Ranges API.

Category	Function	Description
Accessors	start, end, width	Get or set the starts, ends and widths
	names	Get or set the names
	elementMetadata, metadata	Get or set metadata on elements or object
	length	Number of ranges in the vector
	range	Range formed from min(start) and max(end)
Ordering	<, < = , >, > = , = = , ! =	Compare ranges, ordering by start then width
	sort, order, rank	Sort by the ordering defined above
	duplicated	Find ranges with multiple instances
	unique	Find unique instances, removing duplicates
Arithmetic	r+x, r−x, r * x	Shrink or expand ranges r by number x
	shift	Move the ranges by specified amount
	resize	Change width, anchoring on start, end or mid
	distance	Separation between ranges (closest endpoints)
	restrict	Clamp ranges to within some start and end
	flank	Generate adjacent regions on start or end
Set operations	reduce	Merge overlapping and adjacent ranges
	intersect, union, setdiff	Set operations on reduced ranges
	pintersect, punion, psetdiff	Parallel set operations, on each x[i], y[i]
	gaps, pgap	Find regions not covered by reduced ranges
	disjoin	Ranges formed from union of endpoints
Overlaps	findOverlaps	Find all overlaps for each x in y
	countOverlaps	Count overlaps of each x range in y
	nearest	Find nearest neighbors (closest endpoints)
	precede, follow	Find nearest y that x precedes or follows
	x %in% y	Find ranges in x that overlap range in y
Coverage	coverage	Count ranges covering each position
Extraction	r[i]	Get or set by logical or numeric index
	r[[i]]	Get integer sequence from start[i] to end[i]
	subsetByOverlaps	Subset x for those that overlap in y
	head, tail, rev, rep	Conventional R semantics
Split, combine	split	Split ranges by a factor into a *RangesList*
	c	Concatenate two or more range objects

Categorized listing and description of the API for range-based objects, such as *IRanges*, *RangesList*, *GRanges* and *GRangesList*.

**Figure 3:**
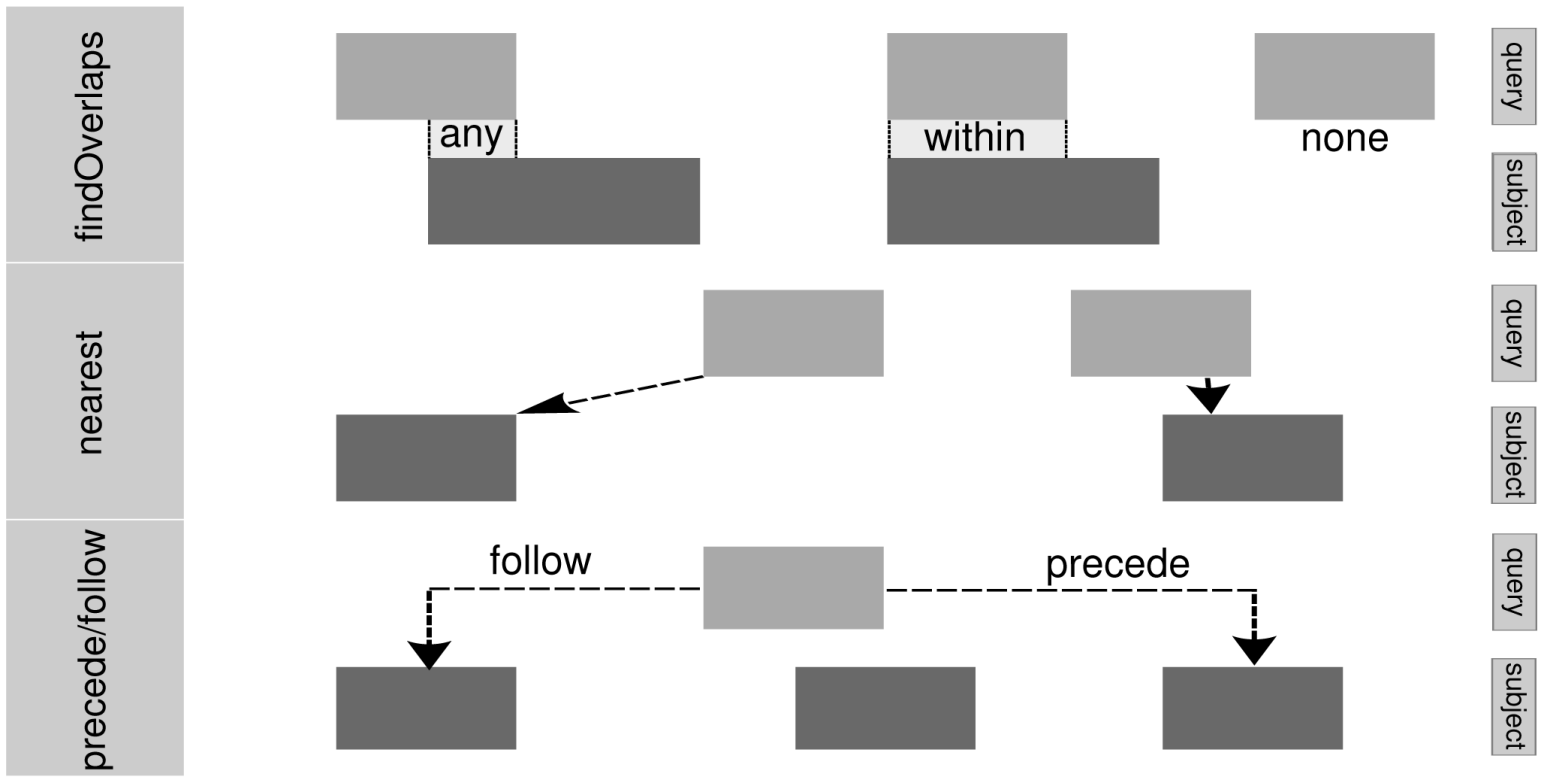
Illustration of overlap (top) and adjacency (bottom) relationships. The *any* mode detects hits with partial or complete overlap, while *within* requires that the query range represents a subregion of the subject range.

A recurrent operation is overlap detection; various instances are illustrated in Figure 3. In later sections, we apply overlap counting for finding the percentage of ChIP-seq peaks that overlap a promoter, counting the number of RNA-seq reads for each transcript, and other tasks. The findOverlaps function uses an efficient interval tree algorithm [4] to detect overlaps between two *IRanges* objects, as well as the more complex range-based data structures introduced later. The algorithm supports several types of overlap, including those defined by Allen's Interval Algebra [5]. The one-time cost of constructing the interval tree is 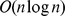, and queries are performed in logarithmic time. In accordance with the vectorized semantics of R, if multiple queries are submitted, they are efficiently processed in batch, without restarting at the root of the tree for each query. The language of implementation is C, which avoids the potentially expensive iteration over the tree in R.

### Working with Genomic Ranges

The *IRanges* class encodes only the start and end of ranges but not the chromosome, strand nor other information that is important in genomic applications. The *GenomicRanges* package adds biological semantics on top of *IRanges*. At its core is the *GRanges* class. Each element of a *GRanges* instance includes a chromosome identifier and strand designation. Each data set is associated with a particular, versioned reference genome sequence consisting of a discrete set of chromosomes or contigs, along with their lengths, if known. The *GRanges* class thus fully represents the data in Figure 1, and encourages best-practices (e.g., tracking genome build) to minimize book-keeping errors.

The *GRanges* class supports many of the same range operations as *IRanges* and specializes them for genomic data. We achieved API specialization by implementing methods for both classes on the same generic functions. In general, we believe method specialization is an effective practice for providing the same interface on top of different data structures. Abstracting implementation details leads to user code that is more robust and easier to write and maintain.

The *GRanges* methods give special consideration to the chromosome and, when appropriate, the strand. For instance, the findOverlaps generic has methods for both *IRanges* and *GRanges*, and the *GRanges* method is specifically able to take advantage of the chromosome information when detecting overlaps. Operations that depend on a notion of direction optionally consider strand. For example, the resize function will resize from the start or end of the ranges in a *IRanges* object. For a *GRanges* object, resize will take the start to be the leftmost position for positive strand features and the rightmost position for negative strand features.

Some types of genomic data, for example gene models or aligned paired-end reads, have a hierarchical structure. To represent this, multiple *GRanges* objects may be combined into a *GRangesList*, where each *GRanges* is considered a compound feature. *GRangesList* groups transcripts by gene, groups exons by transcript, and represents read alignments, where each alignment consists of multiple segments separated by gaps. For example, we group the *KRAS* exons by transcript using a *GRangesList*. A note on performance: although the user interface presents each element of a *GRangesList* as a *GRanges* , internally there is only a single *GRanges*, along with an assoicated partitioning that forms the list elements.

**Figure 4:**
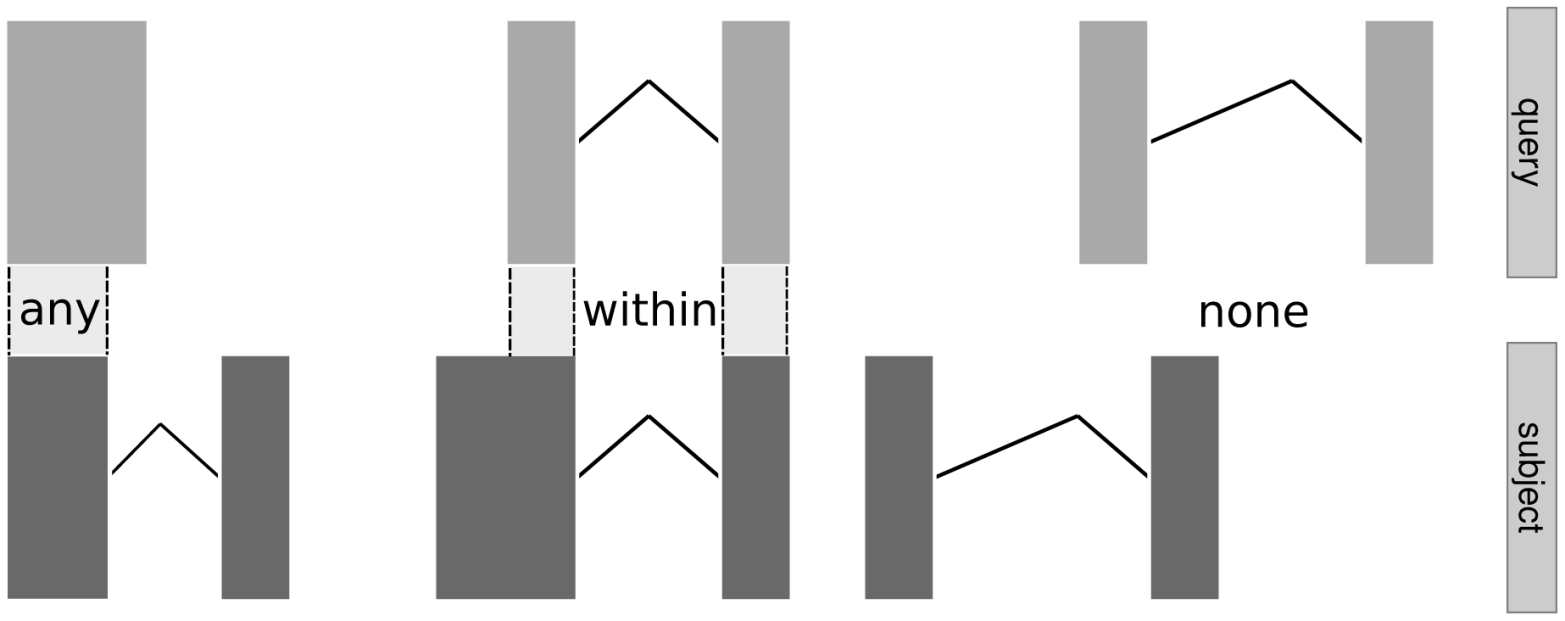
Illustration of overlap computations between two *GRangesList* objects. Each set of rectangles linked by solid lines represents a compound range, i.e., an element of the list. Ranges in the query (top) are being matched against ranges in the subject (bottom). The labels between them indicate the type of overlap (any, within, none).

For a *GRangesList*, overlap detection reports a hit at the element level, i.e., when any range within an element overlaps a query range. See Figure 4 for an illustration. This semantic is convenient, for example, when counting the total number of RNA-seq read pairs overlapping the exonic regions of each transcript. In that case, both the reads and the transcripts are *GRangesList* objects.

### Accessing Gene Models

Recalling our *KRAS* gene model example, there are multiple models for representing transcript structures, and the applicability of each depends on the use case. To support the analyst in asking a broad range of questions, there is a need for a mechanism that draws from a data source of gene annotations and returns them in a variety of different data models. The *GenomicFeatures* package fills that role by distilling multiple data sources into a single database schema and wrapping that database in an API that returns, for example, the exons grouped by gene, or the bounds for every transcript. The databases are implemented in SQLite and are thus accessible from environments outside of R. For reproducibility, a database may be encapsulated in a redistributable R package.

The database is represented by the *TranscriptDb* class and stores the range of each exon, the coding range, the transcript ID, the gene ID, and metadata about the source of the transcript information. The *GenomicFeatures* package provides an automated mechanism for constructing a *TranscriptDb* object from tracks defined in the UCSC genome browser, Biomart, or GTF/GFF files. Bioconductor provides pre-built packages for the most widely adopted gene models, like the UCSC *known gene* annotations on hg19. These packages follow a standard naming convention, e.g., *TxDb.Hsapiens.UCSC.hg19.knownGene*.

There are functions for performing common queries that return the exons, coding regions, and transcript boundaries as a *GRanges* object. Transcript and gene-level groupings are preserved by *GRangesList* objects. The ranges in Figure 1 were derived from the *TxDb.Hsapiens.UCSC.hg19.knownGene* package using the following call to the function exons:

> library(“TxDb.Hsapiens.UCSC.hg19.knownGene”)

> library(“org.Hs.eg.db”)

> kras_gene <- org.Hs.egSYMBOL2EG$KRAS

> kras_exons <- exons(TxDb.Hsapiens.UCSC.hg19.knownGene,

+ vals  =  list(gene_id  =  kras_gene),

+ columns  =  c(“tx_id”, “exon_id”))

To retrieve the exons corresponding to a particular transcript, such as transcript 48666 of KRAS, we call exonsBy, which returns a *GRangesList* of exons grouped by transcript, and extract the element corresponding to the desired transcript identifier:

> exonsByTx <- exonsBy(TxDb.Hsapiens.UCSC.hg19.knownGene)

> krasA <- exonsByTx[[“48666”]]

The contents of krasA are shown in Table 2.

**Table 2:** Contents of the krasA object, representing the exons in isoform A of KRAS.

GRanges with 5 ranges and 3 metadata columns:							
	seqnames	ranges	strand	|	exon_id	exon_name	exon_rank
[1]	chr13	[106118565, 106118681]	+	|	174810	<NA>	1
[2]	chr13	[106119356, 106119490]	+	|	174811	<NA>	2
[3]	chr13	[106124887, 106125034]	+	|	174814	<NA>	3
[4]	chr13	[106142141, 106142541]	+	|	174818	<NA>	4
[5]	chr13	[106143261, 106143383]	+	|	174820	<NA>	5

### Associating Annotations with Ranges

Figure 1 demonstrates how genomic data consist of both ranges and uni- or multivariate annotations on those ranges. In that table, the annotations are the exon ID and a variable grouping the exons into transcripts. If we had read alignments from an RNA-seq experiment, we might use countOverlaps to generate a read count for each exon. Other examples of annotation would include the reference and alternate bases for a single nucleotide variant (SNV) or the position weight matrix (PWM) score for a putative transcription factor binding site.

Every multi-element data structure in the *IRanges* suite supports the storage of per-element metadata: data about data. In this case, the metadata are the annotations, and the primary data are the ranges being annotated. This includes all of the data structures for storing ranges, such as *IRanges, GRanges* and *GRangesList*. The metadata are stored in a *DataFrame* with as many rows as there are elements in the object. We introduce a *DataFrame* class that behaves similarly to the base R *data.frame*, but supports storage of complex vector-like objects (e.g., a *DNAStringSet*, representing DNA sequences, or a *GRanges*) in columns.

### Working with Coverage and Similar Vectors

A common method of summarizing a genomic data set is to calculate the coverage, i.e., the number of features in the data set overlapping each position in the genome. This is useful in ChIP-seq analysis, where many peak detection methods operate on the coverage.

For this example, mouse genomic DNA was cross-linked with DNA-binding proteins, fragmented and precipitated with an antibody for CTCF. An antibody for GFP was used for the control. The CTCF and GFP samples were each sequenced in a single lane on an Illumina sequencer, which generated reads 35 nt in length [6]. We excluded the last 11 nt of each read due to insufficient quality, so the effective read length was 24 nt. The reads were aligned to the mm9 build of the mouse genome using MAQ. We parsed the MAQ output using the *ShortRead* package [7], and the alignments for three chromosomes (chr10, chr11 and chr12) were extracted for use as a demonstration data set in the *chipseq* package [8].

> library(“chipseq”)

> data(“cstest”)

> ctcfReads <- cstest$ctcf

The ctcfReads object, listed in Table 3, is a *GRanges* holding the read alignments from the CTCF sample. The *GRanges* stores the chromosome names, ranges, and strand for each alignment, as well as a list of chromosome names and lengths for the mm9 genome. Tracking the chromosome information guards against errors that could arise, for example, from mixing data across genome assemblies.

**Table 3:** Ranges for the first three reads in the ctcfReads object, storing the read alignments for the CTCF sample.

GRanges with 3 ranges and 0 metadata columns:			
	seqnames	ranges	strand
[1]	chr10	[3012936, 3012959]	+
[2]	chr10	[3012941, 3012964]	+
[3]	chr10	[3012944, 3012967]	+

Each read represents only 24 nt from one end of a fragment of DNA. Since it was the fragment, but not necessarily the sequenced region, that was cross-linked to CTCF, we need to consider the entire fragment when predicting binding sites. We assume that the fragment length was approximately 120 nt and call resize to extend our read ranges to fragment-sized ranges:

> ctcfFragments <- resize(ctcfReads, 120)

Note that the strand of the alignment was automatically taken into account.

The coverage function calculates the coverage for a set of ranges. We calculate the coverage on the CTCF fragments from our ChIP-seq data set as follows:

> ctcfCoverage <- coverage(ctcfFragments)

> ctcfCoverage10 <- ctcfCoverage$chr10

The ctcfCoverage object is a list, with one coverage vector per chromosome. For simplicity, we extract the element corresponding to “chr10”. The ctcfCoverage10 object is of class *Rle*.

Vectors along the genome tend to have many repeated values. For the sake of compactness, we compress the data using a run-length encoding compression scheme. Through the R class system, we abstract this efficient implementation behind an API that supports the features of ordinary R vectors; the complexity is hidden from the user. The *Rle* class represents a run-length encoded vector and provides features beyond those of ordinary vectors. For example, one can use ranges to extract values from an *Rle*. This integrates range-based datasets with data in chromosome-length vectors. To demonstrate, we find the position of the maximum coverage on chr10, and, in order to display the coverage in context, we extract a 5000 nt region centered around that position:

> maxPos <- which.max(ctcfCoverage10)

> roi <- resize(IRanges(maxPos, width = 1), 5000, “center”)

> roiCoverage <- ctcfCoverage$chr10[roi]

**Figure 5:**
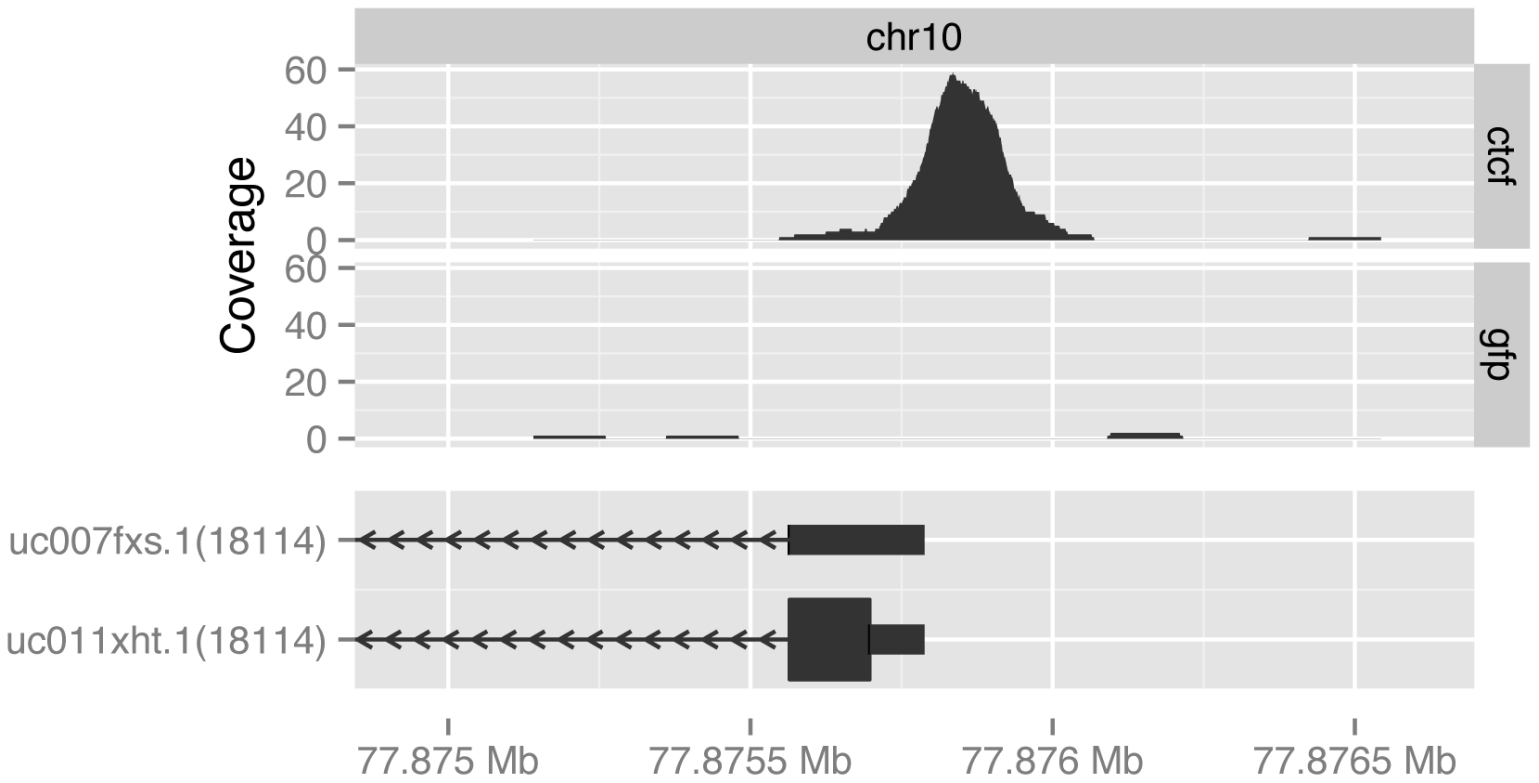
Visualization of the coverage of bases by GFP- and CTCF-bound fragments (top) in the context of part of the gene model for Rrp1, Entrez gene 18114 (bottom).

The roiCoverage vector is plotted in Figure 5 and shows how the coverage relates to the gene context.

Next, we wish to find peaks in the coverage vector by slicing it at a fixed threshold. We call the slice function and pass it a cutoff of 8:

> ctcfPeaks <- slice(ctcfCoverage10, lower  =  8)

The resulting coverage slices are ranges and and we rely on our data structures for managing and manipulating them. In particular, the ctcfPeaks object is a *Views* object, which combines the peak ranges with the original *Rle* coverage vector (another example of integrating ranges with vectors). *Views* has several utilities for summarizing the vector values within each range. We use these to summarize the coverage values within each peak:

> ctcfMaxs <- viewMaxs(ctcfPeaks)

> ctcfSums <- viewSums(ctcfPeaks)

### Accessing Read Alignments

Read alignments may be loaded from a BAM file as a *GappedAlignments* or *GappedAlignmentPairs* object, depending on whether the reads should be treated as paired. Both data structures store short read alignment results in terms of the position, chromosome, strand, CIGAR string (a compact representation of the gaps) and other information. Both also support some of the range operations and can be coerced to *GRanges* and *GRangesList*. The *GRanges* representation holds the ungapped extents of the read alignments, whereas the *GRangesList* represents the alignments as ranges with gaps, including the inter-read gap of a pair and the skipped regions in the reference (e.g., introns). The choice of data structure depends on, for example, whether one wants to count the overlapping pairs, reads, or aligned segments separated by gaps.

A frequent goal of RNA-seq experiments is to estimate the levels of gene expression. For this demonstration, we will simply count the number of read alignments overlapping the exonic regions of each transcript. We begin by loading a BAM file of read alignments from an RNA-seq experiment in yeast [9]. There are four samples, two of which are wildtype and two of which are RLP mutants. The *leeBamViews* package provides the alignments on chromosome XIII from positions 800000 to 900000.

> bams <- getBamsFromLeeBamViews()

> ga <- readGappedAlignments(bams [1] )

> reads <- grglist(ga)

The readGappedAlignments function loads the BAM file as a *GappedAlignments* object, which is then coerced to a *GRangesList* , where each read consists of one or more ranges, separated by intronic gaps.

In the next step, we obtain the transcript annotations for yeast. Bioconductor provides a *TranscriptDb* object for the UCSC sacCer2 assembly, and we extract from it a *GRangesList* representing the transcripts, by calling exonsBy. In order to detect overlaps, the alignments and gene annotations need to have the same chromosome names; we correct for that with the calls to keepSeqlevels and renameSeqlevels.

> library(“TxDb.Scerevisiae.UCSC.sacCer2.sgdGene”)

> tx <- exonsBy(TxDb.Scerevisiae.UCSC.sacCer2.sgdGene)

> tx <- renameSeqlevels(keepSeqlevels(tx, “chrXIII”),

+ c(chrXIII  =  “Scchr13”))

Now that the data and annotations have been loaded, we count the number of read alignments in each genomic feature:

> counts <- countOverlaps(tx, reads, ignore.strand  =  TRUE)

More complex counting algorithms are available via the summarizeOverlaps function, which counts over multiple samples and returns the results as a *SummarizedExperiment* object. Unlike the call to countOverlaps above, reads that map to multiple features are discarded.

### Summarized Experiments

The typical workflow in a genomic data analysis is to reduce a complex raw data set, such as a set of RNA-seq read alignments, to a set of summaries, such as the number of reads aligned over each transcript or exon. Analogously, an exome-seq experiment yields variant calls at particular genomic ranges. It is often appropriate and convenient to store these summaries as a matrix, where the rows correspond to genes or some other genomic feature and the columns to samples. The *SummarizedExperiment* class is designed to hold such values, along with annotations on the genomic regions, the samples and the experiment as a whole. Its design follows the same pattern as the *ExpressionSet* in the Bioconductor microarray infrastructure; the primary difference is that *SummarizedExperiment* is based on *IRanges* data structures. A *SummarizedExperiment* may be constructed directly or generated by a function that executes a high-level workflow. For example, for a list of BAM files, the summarizeOverlaps function counts the overlaps between the read alignments and a database of transcripts.

## Results

In this section we describe range-based integrative computations related to the genetics of protein-DNA binding, and conclude with a topically organized list of Bioconductor packages that make essential use of the ranges infrastructure.

### Exploring Genetics of CTCF Binding

In the murine ChIP-seq example discussed previously, the ranges infrastructure was used to compute and display variation in read coverage over the mouse genome. In this example, we use tools based on the ranges infrastructure to examine both coverage and content of reads from a larger ChIP-seq experiment on human cell lines. Our basic intent is to show how the infrastructure can be used to evaluate the roles of genotype and genetic diversity in the genomic sequences where CTCF is reported to bind, with an understanding that ultimate inferences on protein binding locations and on base-call distributions over heterozygous loci will need to directly incorporate risks of base calling and read mapping errors, and will need to be followed up with wet-lab validation.

For an investigation of the prevalence of allele-specific protein-DNA binding [10], BAM files on 22 ChIP-seq experiments addressing CTCF binding to DNA from immortalized B-cells were collected from the ENCODE project portal. Exclusion of files with aberrant quality score distributions left 16 BAM files corresponding to 12 distinct individuals; two technical replicates were available for each of four individuals. Furthermore, different base-call quality score scales were used for two batches of samples; by subtracting 31 from the reported mean quality scores for one set of samples, approximately identical medians and interquartile ranges were established for mean quality scores for all retained samples. Base calls for reads bound at all genomic locations with positive coverage were tabulated using the *VariantTools* tallyVariants function, and reduced to locations exhibiting statistical evidence of allele-dependent CTCF binding using the callVariants function. Each of these variant assessment tools makes use of infrastructure derived from GSNAP [11] with key results materialized as *GRanges* instances. Table 4 depicts an excerpt from a *callVariants* output.

**Table 4:** Partial output of countVariants applied to a BAM file from an ENCODE CTCF ChIP-seq experiment.

GRanges with 8 ranges and 5 metadata columns:									
seqnames		ranges strand		|	ref	alt	ncycles	count	count.ref
NA06990_2	chr1	[11391, 11391]	+	|	T	A	7	19	5
NA06990_2	chr1	[793522, 793522]	+	|	T	A	1	4	10
NA06990_2	chr1	[825860, 825860]	+	|	G	A	1	4	5
NA06990_2	chr1	[968600, 968600]	+	|	A	C	2	5	6
NA06990_2	chr1	[1057713, 1057713]	+	|	A	C	3	4	19
NA06990_2	chr1	[1376423, 1376423]	+	|	G	C	5	5	53
NA06990_2	chr1	[1376430, 1376430]	+	|	T	C	4	4	51
NA06990_2	chr1	[1610542, 1610542]	+	|	A	C	4	4	28

The GRanges instance includes location-specific information on 24 attributes of each call, including information on sequencer cycle, base call quality distribution, and other features of BAM-based variant calling as performed by GSNAP [11].

The genome-wide searches for allele-dependent CTCF binding events employed default settings for variant calling by *VariantTools* callVariants, which include criteria on minimum coverage, minimum diversity of read cycles at which base is found, and limitation of risk of strand bias. This process yielded a total of 19655 locations with evidence of allele-dependent CTCF binding, corresponding to 50750 events over the 12 individuals. We obtained GRanges representations of dbSNP build 137 with the scanVcf function of the *VariantAnnotation*. This facilitated distributed computation for partitioning allele-dependent CTCF binding events into 12691 coincident with known polymorphisms and 6964 at locations where no SNP has been reported in dbSNP.

**Figure 6:**
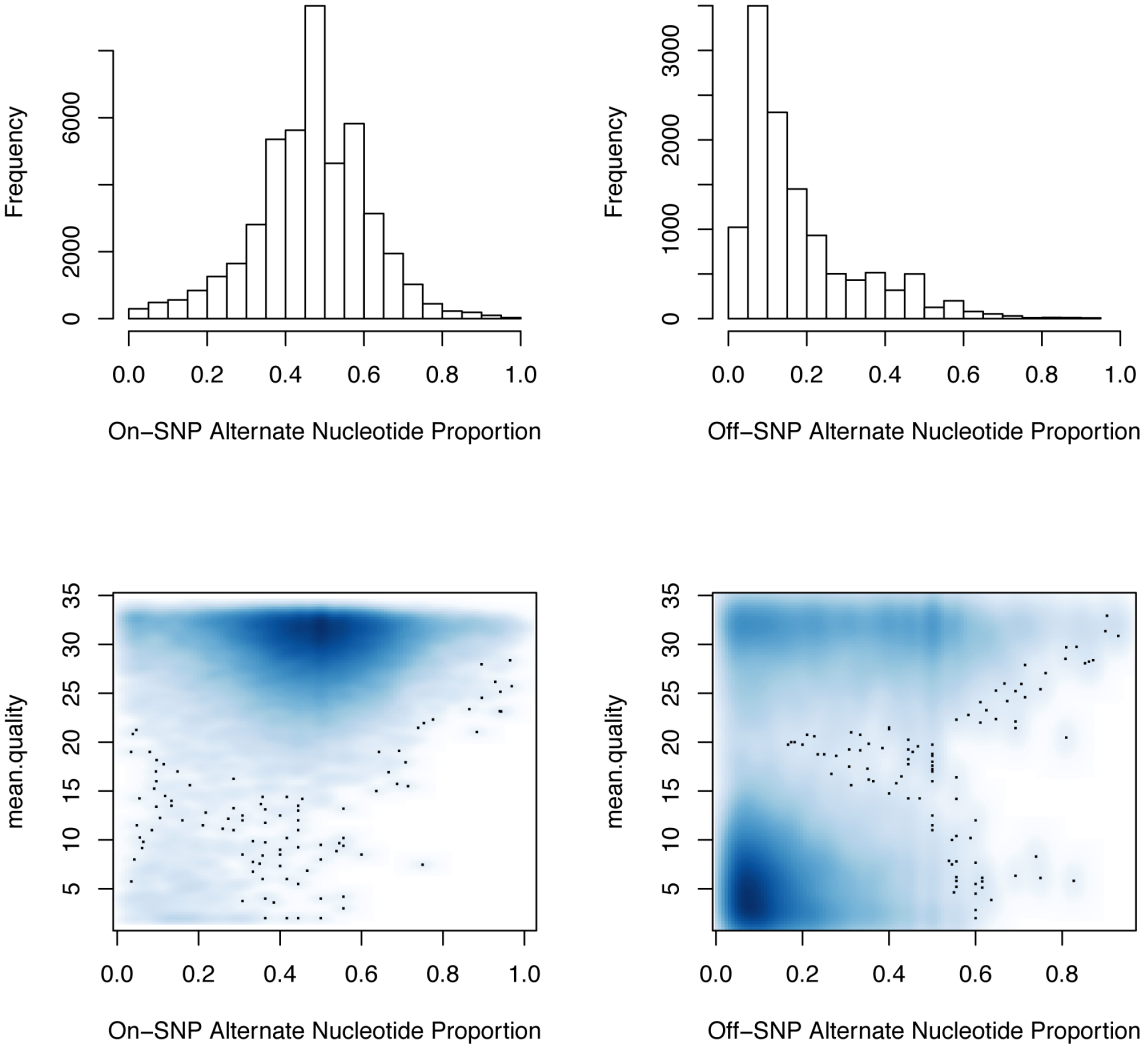
Top panels: distributions of alternate nucleotide proportions for on- and off-SNP allele-dependent CTCF binding events. Bottom panels: relationships between average call quality values and alternate nucleotide proportions are depicted using a 2D density estimate (darker regions correspond to higher density.).

Allelic imbalance in CTCF binding corresponds to departure of the alternate nucleotide proportion (ANP) at a CTCF binding site from 50%. The upper panels of Figure 6 show the distributions of ANP stratified by coincidence of allele-dependent CTCF binding locations with locations of known SNP. The lower panels show identically stratified associations between ANP and mean base-call qualities for pileups over the allele-dependent binding locations. While the off-SNP locations show a proponderance of ANP below 20%, there is also an indication that base-call quality for such binding events is relatively low, implying that these findings would be unlikely to replicate. For example, among on-SNP allele-dependent binding calls with ANP below 20%, 5% had mean quality less than 5; the corresponding frequency for low-quality on-SNP allele-dependent binding locations calls was 41%.

Figure 6 was constructed using the packages and features described above with very little programming effort needed to specialize the computations to this example. Most computational biologists and other interested investigators could easily carry out these steps. We can conclude that allelic imbalance in CTCF binding events is frequently detectable but, for the data considered, the apparent imbalances observed are likely a mix of real biology and technical artifacts originating from, e.g., sequencing and read mapping errors. Careful analysis of metadata collected in the variant-calling process may help to disentangle the key factors contributing to allele-dependent CTCF binding.

### Software Based on the Infrastructure

There is a growing ecosystem of packages based on this infrastructure. By current count, more than 80 packages depend directly on the packages presented here. This includes packages for input and output of ranges ( *rtracklayer* , *Rsamtools* ) [12], [13], quality assessment ( *ShortRead* ) [7], sequence analysis ( *Biostrings* ) [14], variant calling ( *VariantTools* ) [15], and other tasks. To summarize the different use cases addressed by dependent packages, Table 5 tabulates the descriptive labels chosen from a controlled vocabulary by the package authors.

**Table 5:** Selected packages based on the Ranges infrastructure.

Term	Count	Example packages
Genetics	16	NarrowPeaks, nucleR, GenomicFeatures, mosaics
Preprocessing	11	MEDIPS, biovizBase, TSSi, HMMcopy
Infrastructure	9	Genominator, nnotationDbi, ggbio, dInfoBuilder
GeneExpression	8	GGtools, easyRNASeq, Repitools, TransView
Sequencing	5	girafe, triform, seqbias, rSFFreader
Microarray	4	methyAnalysis, Gviz, MinimumDistance, charm
Clustering	4	chroGPS, methVisual, DirichletMultinomial, PICS
GenomicSequence	3	rGADEM, MotifDb, MotIV
QualityControl	3	ShortRead, R453Plus1Toolbox, htSeqTools
Statistics	2	oneChannelGUI, PING
OneChannel	2	xmapcore, annmap
DataRepresentation	2	genoset, FunciSNP
GeneticVariability	2	VanillaICE, SNPchip
Bioinformatics	2	DiffBind, segmentSeq
ChIPseq	2	chipseq, BayesPeak
Other	10	ChromHeatMap, gwascat, ChIPpeakAnno, OTUbase

Categories are biocViews terms. Up to 4 packages were randomly sampled from Bioconductor packages that explicitly declare a dependence on *IRanges*, *GenomicRanges*, or *GenomicFeatures* packages.

## Availability and Future Directions

All of the packages described, including *IRanges*, *GenomicRanges* and *GenomicFeatures*, form the core infrastructure for sequence analysis in Bioconductor and are available from the project website: http://bioconductor.org (see also Software S1-S3). We aim to continue to support scientists in their drive to further science by asking increasingly complex and integrative questions about increasingly complex and heterogeneous data. For example, we are working towards better support for detecting alternative and novel splicing, measuring isoform-specific expression, annotating sequence variants, mapping between genome, transcript and protein coordinate spaces, and integrating transcript annotations with gene-level metadata. There is also an unmet need in the visualization of genomic ranges. In particular, we need better visualizations for relating RNA-seq coverage and junction counts to transcript structures, and for diagnosing read alignments in the context of variant calling. Finally, as datasets continue to expand in size, we continue to seek more efficient algorithms and data structures, and we are vigilant for opportunities to leverage parallel computing.

## Supporting Information

Software S1 **The IRanges package.** The *IRanges* package provides efficient low-level and reusable S4 classes for storing and manipulating ranges of integers and compressed, genome-length vectors.
(GZ) Click here for additional data file.

Software S2 **The GenomicRanges package.** The *GenomicRanges* package defines general purpose containers for storing genomic ranges as well as more specialized containers for storing alignments against a reference genome.
(GZ) Click here for additional data file.

Software S3 **The GenomicFeatures package.** The *GenomicFeatures* package is a set of tools and methods for making and manipulating transcript-centric annotations.
(GZ) Click here for additional data file.
