# Supplementary material for: Software for Computing and Annotating Genomic Ranges
Source: PLoS Comput Biol. 2013 Aug 8;9(8):e1003118. doi: 10.1371/journal.pcbi.1003118 (PMC3738458; doi:10.1371/journal.pcbi.1003118)
Supplement: Software S1 — The IRanges package. The IRanges package provides efficient low-level and reusable S4 classes for storing and manipulating ranges of integers and compressed, genome-length vectors. (GZ) [file pcbi.1003118.s001.gz › IRanges/inst/doc/IRangesOverview.pdf]

An Introduction to *IRanges*

Patrick Aboyoun, Michael Lawrence, Hervé Pagès

May 10, 2013

1 Introduction

The *IRanges* package is designed to represent sequences, ranges representing indices along those sequences, and data related to those ranges. In this vignette, we will rely on simple, illustrative example datasets, rather than large, real-world data, so that each data structure and algorithm can be explained in an intuitive, graphical manner. We expect that packages that apply *IRanges* to a particular problem domain will provide vignettes with relevant, realistic examples.

The *IRanges* package is available at bioconductor.org and can be downloaded via `biocLite`:

```
> source("http://bioconductor.org/biocLite.R")
> biocLite("IRanges")
```

```
> library(IRanges)
```

2 Vector objects

In the context of the *IRanges* package, a sequence is an ordered finite collection of elements. The *IRanges* packages represents two types of objects as sequences: (1) atomic sequences and (2) lists (or non-atomic sequences). The following subsections describe each in turn. All *IRanges*-derived sequences inherit from the *Vector* virtual class.

2.1 Atomic Vectors

In R, atomic sequences are typically stored in atomic vectors. The *IRanges* package includes two additional atomic sequence object types: *Rle*, which compresses an atomic sequence through run-length encoding, and *XVector*, which refers to its data through an external pointer. *XVector* and its derivatives are considered low-level infrastructure and, as such, will not be covered by this vignette.

We begin our discussion of atomic sequences using two R *integer* vectors.

```
> set.seed(0)
> lambda <- c(rep(0.001, 4500), seq(0.001, 10, length = 500),
+             seq(10, 0.001, length = 500))
> xVector <- rpois(1e7, lambda)
> yVector <- rpois(1e7, lambda[c(251:length(lambda), 1:250)])
```

All atomic sequences in R have three main properties: (1) a notion of length or number of elements, (2) the ability to extract elements to create new atomic sequences, and (3) the ability to be combined with one or more atomic sequences to form larger atomic sequences. The main functions for these three operations are `length`, `[`, and `c`.

```
> length(xVector)
```

```
[1] 10000000
```

```
> xVector[1]
```

```
[1] 0
```

```
> zVector <- c(xVector, yVector)
```

While these three methods may seem trivial, they provide a great deal of power and many atomic sequence manipulations can be constructed using them.

2.1.1 Vector Subsetting

As with ordinary R atomic vectors, it is often necessary to subset one sequence from another. When this subsetting does not duplicate or reorder the elements being extracted, the result is called a *subsequence*. In general, the `[` function can be used to construct a new sequence or extract a subsequence, but its interface is often inconvenient and not amenable to optimization. To compensate for this, the *IRanges* package supports seven additional functions for sequence extraction:

1. `window` - Produces a subsequence over a specified region with or without regular interval subsampling.
2. `seqselect` - Concatenates multiple consecutive subsequences into a new sequence. The result may or may not be a subsequence.
3. `subset` - Extracts the subsequence specified by a logical vector.
4. `head` - Extracts a consecutive subsequence containing the first n elements.
5. `tail` - Extracts a consecutive subsequence containing the last n elements.
6. `rev` - Creates a new sequence with the elements in the reverse order.
7. `rep` - Creates a new sequence by repeating sequence elements.

The following code illustrates how these functions are used on an ordinary R *integer* vector:

```
> xSnippet <- window(xVector, start = 4751, end = 4760)
> xSnippet
```

```
 [1] 4 6 5 4 6 2 6 7 5 5
```

```
> head(xSnippet)
```

```
[1] 4 6 5 4 6 2
```

```
> tail(xSnippet)
```

```
[1] 6 2 6 7 5 5
```

```
> rev(xSnippet)
```

```
 [1] 5 5 7 6 2 6 4 5 6 4
```

```
> rep(xSnippet, 2)
```

```
 [1] 4 6 5 4 6 2 6 7 5 5 4 6 5 4 6 2 6 7 5 5
```

```
> window(xSnippet, delta = 2)
```

```
[1] 4 5 6 6 5
```

```
> seqselect(xSnippet, start = c(6,1), end = c(10, 5))
```

```
 [1] 2 6 7 5 5 4 6 5 4 6
```

```
> subset(xSnippet, xSnippet >= 5L)
```

```
[1] 6 5 6 6 7 5 5
```

2.1.2 Combining Vectors

The *IRanges* package uses two generic functions, `c` and `append`, for combining two *Vector* objects. The methods for *Vector* objects follow the definition that these two functions are given the the *base* package.

```
> c(xSnippet, rev(xSnippet))
```

```
 [1] 4 6 5 4 6 2 6 7 5 5 5 5 7 6 2 6 4 5 6 4
```

```
> append(xSnippet, xSnippet, after = 3)
```

```
 [1] 4 6 5 4 6 5 4 6 2 6 7 5 5 4 6 2 6 7 5 5
```

2.1.3 Looping over Vectors and Vector subsets

In R, `for` looping can be an expensive operation. To compensate for this, *IRanges* uses three generics, `endoapply`, `lapply`, and `sapply`, for looping over sequences and two generics, `aggregate` and `shiftApply`, to perform calculations over subsequences. The `lapply` and `sapply` functions are familiar to many R users since they are the standard functions for looping over the elements of an R *list* object. The `endoapply` function performs an endomorphism equivalent to `lapply`, i.e. returns a *Vector* object of the same class as the input rather than a *list* object. More will be given on these three functions in the Lists subsection.

The `aggregate` function combines sequence extraction functionality of the `window` function with looping capabilities of the `sapply` function. For example, here is some code to compute medians across a moving window of width 3 using the function `aggregate`:

```
> xSnippet
```

```
 [1] 4 6 5 4 6 2 6 7 5 5
```

```
> aggregate(xSnippet, start = 1:8, width = 3, FUN = median)
```

```
[1] 5 5 5 4 6 6 6 5
```

The `shiftApply` function is a looping operation involving two sequences whose elements are lined up via a positional shift operation. For example, the elements of `xVector` and `yVector` were simulated from Poisson distributions with the mean of element i from `yVector` being equivalent to the mean of element i + 250 from `xVector`. If we did not know the size of the shift, we could estimate it by finding the shift that maximizes the correlation between `xVector` and `yVector`.

```
> cor(xVector, yVector)
```

```
[1] 0.5739224
```

```
> shifts <- seq(235, 265, by=3)
> corrs  <- shiftApply(shifts, yVector, xVector, FUN = cor)
```

```
> plot(shifts, corrs)
```

The result is shown in Fig. 1.

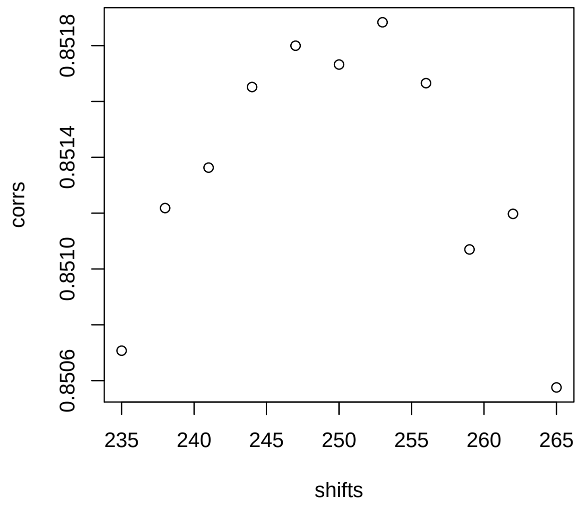

Figure 1: Correlation between `xVector` and `yVector` for various shifts.

2.1.4 Run Length Encoding

Up until this point we have used R atomic vectors to represent atomic sequences, but there are times when these object become too large to manage in memory. When there are lots of consecutive repeats in the sequence, the data can be compressed and managed in memory through a run-length encoding where a data value is paired with a run length. For example, the sequence {1, 1, 1, 2, 3, 3} can be represented as values = {1, 2, 3}, run lengths = {3, 1, 2}.

The *Rle* class in *IRanges* is used to represent a run-length encoded (compressed) sequence of *logical*, *integer*, *numeric*, *complex*, *character*, or *raw* values. One way to construct an *Rle* object is through the *Rle* constructor function:

```
> xRle <- Rle(xVector)
> yRle <- Rle(yVector)
> xRle

integer-Rle of length 10000000 with 1510219 runs
  Lengths:  780    1  208    1 1599    1 ...    5    1   91    1  927
  Values :    0    1    0    1    0    1 ...    0    1    0    1    0

> yRle

integer-Rle of length 10000000 with 1511351 runs
  Lengths: 1003    1  413    1  896    1 ...    3    1  845    1  419
  Values :    0    1    0    1    0    1 ...    0    1    0    1    0
```

When there are lots of consecutive repeats, the memory savings through an RLE can be quite dramatic. For example, the `xRle` object occupies less than one quarter of the space of the original `xVector` object, while storing the same information:

```
> as.vector(object.size(xRle) / object.size(xVector))
```

```
[1] 0.3020711
```

```
> identical(as.vector(xRle), xVector)
```

```
[1] TRUE
```

The functions `runValue` and `runLength` extract the run values and run lengths from an *Rle* object respectively:

```
> head(runValue(xRle))
```

```
[1] 0 1 0 1 0 1
```

```
> head(runLength(xRle))
```

```
[1]  780    1  208    1 1599    1
```

The *Rle* class supports many of the basic methods associated with R atomic vectors including the Ops, Math, Math2, Summary, and Complex group generics. Here is a example of manipulating *Rle* objects using methods from the Ops group:

```
> xRle > 0
```

```
logical-Rle of length 10000000 with 197127 runs
  Lengths:   780     1   208     1  1599 ...     1    91     1   927
  Values : FALSE  TRUE FALSE  TRUE FALSE ...  TRUE FALSE  TRUE FALSE
```

```
> xRle + yRle
```

```
integer-Rle of length 10000000 with 1957707 runs
  Lengths: 780   1 208   1  13   1 413 ...   5   1  91   1 507   1 419
  Values :   0   1   0   1   0   1   0 ...   0   1   0   1   0   1   0
```

```
> xRle > 0 | yRle > 0
```

```
logical-Rle of length 10000000 with 210711 runs
  Lengths:   780     1   208     1    13 ...     1   507     1   419
  Values : FALSE  TRUE FALSE  TRUE FALSE ...  TRUE FALSE  TRUE FALSE
```

Here are some from the Summary group:

```
> range(xRle)
```

```
[1]  0 26
```

```
> sum(xRle > 0 | yRle > 0)
```

```
[1] 2105185
```

And here is one from the Math group:

```
> log1p(xRle)
```

```
numeric-Rle of length 10000000 with 1510219 runs
  Lengths:               780                 1 ...               927
  Values :                 0 0.693147180559945 ...                 0
```

As with the atomic vectors, the `cor` and `shiftApply` functions operate on *Rle* objects:

```
> cor(xRle, yRle)
```

```
[1] 0.5739224
```

```
> shiftApply(249:251, yRle, xRle, FUN = function(x, y) var(x, y) / (sd(x) * sd(y)))
```

```
[1] 0.8519138 0.8517324 0.8517725
```

For more information on the methods supported by the *Rle* class, consult the `Rle` man page.

2.2 Lists

In many data analysis situation there is a desire to organize and manipulate multiple objects simultaneously. Typically this is done in R through the usage of a list. While a list serves as a generic container, it does not confer any information about the specific class of its elements, provides no infrastructure to ensure type safety, and the S3 and S4 method dispatch mechanisms do not support method selection for lists with homogeneous object types. The *List* virtual class defined in the *IRanges* package addresses these issues. *List* is a direct extension of *Vector*.

2.2.1 Lists of Atomic Vectors

The first type of lists we consider are those containing atomic sequences such as *integer* vectors or *Rle* objects. We may wish to define a method that retrieves the length of each atomic sequence element, without special type checking. To enable this, we define collection classes such as *IntegerList* and *RleList*, which inherit from the *List* virtual class, for representing lists of *integer* vectors and *Rle* objects respectively.

```
> getClassDef("RleList")
```

```
Virtual Class "RleList" [package "IRanges"]

Slots:

Name:      elementType elementMetadata        metadata
Class:       character DataTableORNULL            list

Extends:
Class "AtomicList", directly
Class "List", by class "AtomicList", distance 2
Class "Vector", by class "AtomicList", distance 3
Class "Annotated", by class "AtomicList", distance 4

Known Subclasses: "RleViews", "CompressedRleList", "SimpleRleList"
```

As the class definition above shows, the *RleList* class is virtual with subclasses *SimpleRleList* and *CompressedRleList*. A *SimpleRleList* class uses a regular R list to store the underlying elements and the *CompressedRleList* class stores the elements in an unlisted form and keeps track of where the element breaks are. The former "simple list" class is useful when the Rle elements are long and the latter "compressed list" class is useful when the list is long and/or sparse (i.e. a number of the list elements have length 0).

In fact, all of the atomic vector types (raw, logical, integer, numeric, complex, and character) have similar list classes that derive from the *List* virtual class. For example, there is an *IntegerList* virtual class with subclasses *SimpleIntegerList* and *CompressedIntegerList*.

Each of the list classes for atomic sequences, be they stored as vectors or *Rle* objects, have a constructor function with a name of the appropriate list virtual class, such as *IntegerList*, and an optional argument `compress` that takes an argument to specify whether or not to create the simple list object type or the compressed list object type. The default is to create the compressed list object type.

```
> args(IntegerList)

function (..., compress = TRUE)
NULL

> cIntList1 <- IntegerList(x = xVector, y = yVector)
> cIntList1

IntegerList of length 2
[["x"]] 0 0 0 0 0 0 0 0 0 0 0 0 0 0 0 ... 0 0 0 0 0 0 0 0 0 0 0 0 0 0 0
[["y"]] 0 0 0 0 0 0 0 0 0 0 0 0 0 0 0 ... 0 0 0 0 0 0 0 0 0 0 0 0 0 0 0

> sIntList2 <- IntegerList(x = xVector, y = yVector, compress = FALSE)
> sIntList2

IntegerList of length 2
[["x"]] 0 0 0 0 0 0 0 0 0 0 0 0 0 0 0 ... 0 0 0 0 0 0 0 0 0 0 0 0 0 0 0
[["y"]] 0 0 0 0 0 0 0 0 0 0 0 0 0 0 0 ... 0 0 0 0 0 0 0 0 0 0 0 0 0 0 0

> ## sparse integer list
> xExploded <- lapply(xVector[1:5000], function(x) seq_len(x))
> cIntList2 <- IntegerList(xExploded)
> sIntList2 <- IntegerList(xExploded, compress = FALSE)
> object.size(cIntList2)

33080 bytes

> object.size(sIntList2)

253952 bytes
```

The `length` function returns the number of elements in a *Vector*-derived object and, for a *List*-derived object like “simple list” or “compressed list”, the `elementLengths` function returns an integer vector containing the lengths of each of the elements:

```
> length(cIntList2)

[1] 5000

> Rle(elementLengths(cIntList2))

integer-Rle of length 5000 with 427 runs
  Lengths:  780    1  208    1 1599    1 ...    1    1    1    1    1
  Values :    0    1    0    1    0    1 ...   10    9    6    9   12
```

Just as with ordinary R *list* objects, *List*-derived object support the `[[` for element extraction, `c` for combining, and `lapply`/`sapply` for looping. When looping over sparse lists, the "compressed list" classes can be much faster during computations since only the non-empty elements are looped over during the `lapply`/`sapply` computation and all the empty elements are assigned the appropriate value based on their status.

```
> system.time(sapply(xExploded, mean))

   user  system elapsed
  0.268   0.000   0.266

> system.time(sapply(sIntList2, mean))

   user  system elapsed
  0.300   0.000   0.301

> system.time(sapply(cIntList2, mean))

   user  system elapsed
  0.276   0.000   0.279

> identical(sapply(xExploded, mean), sapply(sIntList2, mean))

[1] TRUE

> identical(sapply(xExploded, mean), sapply(cIntList2, mean))

[1] TRUE
```

Unlist ordinary R *list* objects, *AtomicList* objects support the `Ops` (e.g. `+`, `==`, `&`), `Math` (e.g. `log`, `sqrt`), `Math2` (e.g. `round`, `signif`), `Summary` (e.g. `min`, `max`, `sum`), and `Complex` (e.g. `Re`, `Im`) group generics.

```
> xRleList <- RleList(xRle, 2L * rev(xRle))
> yRleList <- RleList(yRle, 2L * rev(yRle))
> xRleList > 0

RleList of length 2
[[1]]
logical-Rle of length 10000000 with 197127 runs
  Lengths:   780     1   208     1  1599 ...     1    91     1   927
  Values : FALSE  TRUE FALSE  TRUE FALSE ...  TRUE FALSE  TRUE FALSE

[[2]]
logical-Rle of length 10000000 with 197127 runs
  Lengths:   927     1    91     1     5 ...     1   208     1   780
  Values : FALSE  TRUE FALSE  TRUE FALSE ...  TRUE FALSE  TRUE FALSE

> xRleList + yRleList

RleList of length 2
[[1]]
integer-Rle of length 10000000 with 1957707 runs
  Lengths: 780   1 208   1  13   1 413 ...   5   1  91   1 507   1 419
  Values :   0   1   0   1   0   1   0 ...   0   1   0   1   0   1   0
```

```
[[2]]
integer-Rle of length 10000000 with 1957707 runs
  Lengths: 419   1 507   1  91   1   5 ... 413   1  13   1 208   1 780
  Values :   0   2   0   2   0   2   0 ...   0   2   0   2   0   2   0
```

```
> sum(xRleList > 0 | yRleList > 0)
```

```
[1] 2105185 2105185
```

Since these atomic lists inherit from *List*, they can also use the looping function `endoapply` to perform endomorphisms.

```
> safe.max <- function(x) { if(length(x)) max(x) else integer(0) }
> endoapply(sIntList2, safe.max)
```

```
IntegerList of length 5000
[[1]] integer(0)
[[2]] integer(0)
[[3]] integer(0)
[[4]] integer(0)
[[5]] integer(0)
[[6]] integer(0)
[[7]] integer(0)
[[8]] integer(0)
[[9]] integer(0)
[[10]] integer(0)
...
<4990 more elements>
```

```
> endoapply(cIntList2, safe.max)
```

```
IntegerList of length 5000
[[1]] integer(0)
[[2]] integer(0)
[[3]] integer(0)
[[4]] integer(0)
[[5]] integer(0)
[[6]] integer(0)
[[7]] integer(0)
[[8]] integer(0)
[[9]] integer(0)
[[10]] integer(0)
...
<4990 more elements>
```

```
> endoapply(sIntList2, safe.max)[[1]]
```

```
integer(0)
```

3 Data Tables

To Do: *DataTable*, *DataFrame*, *DataFrameList*, *SplitDataFrameList*

4 Vector Annotations

Often when one has a collection of objects, there is a need to attach metadata that describes the collection in some way. Two kinds of metadata can be attached to a *Vector* object:

1. Metadata about the object as a whole: this metadata is accessed via the `metadata` accessor and is represented as an ordinary *list*;

2. Metadata about the individual elements of the object: this metadata is accessed via the `mcols` accessor (`mcols` stands for *metadata columns*) and is represented as a *DataTable* object (i.e. as an instance of a concrete subclass of *DataTable*, e.g. a *DataFrame* object). This *DataTable* object can be thought of as the result of binding together one or several vector-like objects (the metadata columns) of the same length as the *Vector* object. Each row of the *DataTable* object annotates the corresponding element of the *Vector* object.

5 Vector Ranges

When analyzing sequences, we are often interested in particular consecutive subsequences. For example, the a vector could be considered a sequence of lower-case letters, in alphabetical order. We would call the first five letters (a to e) a consecutive subsequence, while the subsequence containing only the vowels would not be consecutive. It is not uncommon for an analysis task to focus only on the geometry of the regions, while ignoring the underlying sequence values. A list of indices would be a simple way to select a subsequence. However, a sparser representation for consecutive subsequences would be a range, a pairing of a start position and a width, as used when extracting sequences with `window` and `seqselect`, above.

When analyzing subsequences in *IRanges*, each range is treated as an observation. The virtual *Ranges* class represents lists of ranges, or, equivalently and as a derivative *IntegerList*, sequences of consecutive integers. The most commonly used implementation of *Ranges* is *IRanges*, which stores the starts and widths as ordinary integer vectors. To construct an *IRanges* instance, we call the `IRanges` constructor. Ranges are normally specified by passing two out of the three parameters: start, end and width (see `help(IRanges)` for more information).

```
> ir1 <- IRanges(start = 1:10, width = 10:1)
> ir2 <- IRanges(start = 1:10, end = 11)
> ir3 <- IRanges(end = 11, width = 10:1)
> identical(ir1, ir2) & identical(ir2, ir3)
```

```
[1] FALSE
```

```
> ir <- IRanges(c(1, 8, 14, 15, 19, 34, 40),
+   width = c(12, 6, 6, 15, 6, 2, 7))
```

All of the above calls construct an *IRanges* instance with the same ranges, using different combinations of the `start`, `end` and `width` parameters.

Accessing the starts, widths and ends is supported by every *Ranges* implementation.

```
> start(ir)
```

```
[1]  1  8 14 15 19 34 40
```

```
> end(ir)
```

```
[1] 12 13 19 29 24 35 46
```

```
> width(ir)
```

```
[1] 12  6  6 15  6  2  7
```

For *IRanges* and some other *Ranges* derivatives, subsetting is also supported, by numeric and logical indices.

```
> ir[1:4]

IRanges of length 4
    start end width
[1]     1  12    12
[2]     8  13     6
[3]    14  19     6
[4]    15  29    15

> ir[start(ir) <= 15]

IRanges of length 4
    start end width
[1]     1  12    12
[2]     8  13     6
[3]    14  19     6
[4]    15  29    15
```

One may think of each range as a sequence of integer ranges, and *Ranges* is, in fact, derived from *IntegerList*.

```
> ir[[1]]

 [1]  1  2  3  4  5  6  7  8  9 10 11 12
```

In order to illustrate range operations, we'll create a function to plot ranges.

```
> plotRanges <- function(x, xlim = x, main = deparse(substitute(x)),
+                         col = "black", sep = 0.5, ...)
+ {
+   height <- 1
+   if (is(xlim, "Ranges"))
+     xlim <- c(min(start(xlim)), max(end(xlim)))
+   bins <- disjointBins(IRanges(start(x), end(x) + 1))
+   plot.new()
+   plot.window(xlim, c(0, max(bins)*(height + sep)))
+   ybottom <- bins * (sep + height) - height
+   rect(start(x)-0.5, ybottom, end(x)+0.5, ybottom + height, col = col, ...)
+   title(main)
+   axis(1)
+ }

> plotRanges(ir)
```

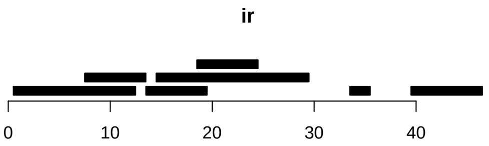

Figure 2: Plot of original ranges.

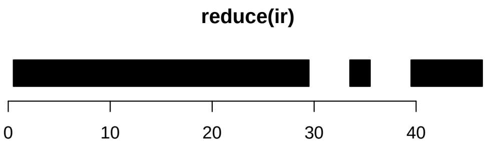

Figure 3: Plot of reduced ranges.

5.1 Normality

Sometimes, it is necessary to formally represent a subsequence, where no elements are repeated and order is preserved. Also, it is occasionally useful to think of a *Ranges* object as a set, where no elements are repeated and order does not matter. While every *Ranges* object, as a *Vector* derivative, has an implicit ordering, one can enforce the same ordering for all such objects, so that ordering becomes inconsequential within that context.

The *NormalIRanges* class formally represents either a subsequence encoding or a set of integers. By definition a Ranges object is said to be *normal* when its ranges are: (a) not empty (i.e. they have a non-null width); (b) not overlapping; (c) ordered from left to right; (d) not even adjacent (i.e. there must be a non empty gap between 2 consecutive ranges).

There are three main advantages of using a *normal Ranges* object: (1) it guarantees a subsequence encoding or set of integers, (2) it is compact in terms of the number of ranges, and (3) it uniquely identifies its information, which simplifies comparisons.

The `reduce` function reduces any *Ranges* object to a *NormalIRanges* by merging redundant ranges.

```
> reduce(ir)
```

```
IRanges of length 3
    start end width
[1]     1  29    29
[2]    34  35     2
[3]    40  46     7
```

```
> plotRanges(reduce(ir))
```

5.2 Lists of *Ranges* objects

It is common to manipulate collections of *Ranges* objects during an analysis. Thus, the *IRanges* package defines some specific classes for working with multiple *Ranges* objects.

The *RangesList* class asserts that each element is a *Ranges* object and provides convenience methods, such as `start`, `end` and `width` accessors that return *IntegerList* objects, aligning with the *RangesList* object. To explicitly construct a *RangesList*, use the `RangesList` function.

```
> rl <- RangesList(ir, rev(ir))
```

```
> start(rl)
```

```
IntegerList of length 2
[[1]] 1 8 14 15 19 34 40
[[2]] 40 34 19 15 14 8 1
```

5.3 Vector Extraction

As the elements of *Ranges* objects encode consecutive subsequences, they may be used directly in sequence extraction. Note that when a *normal Ranges* is given as the index, the result a subsequence, as no elements are repeated or reordered.

```
> irextract <- IRanges(start = c(4501, 4901) , width = 100)
> seqselect(xRle, irextract)
```

```
integer-Rle of length 200 with 159 runs
  Lengths: 12  1  1  1  2  1  1  1  1  2 ...  1  1  1  1  1  1  1  1  1
  Values :  0  1  0  2  0  1  0  1  0  1 ...  9 12  6  5 10  9  6  9 12
```

If the sequence is a *Vector* subclass (i.e. not an ordinary *vector*), the canonical `[` function also accepts a *Ranges* instance.

```
> xRle[irextract]
```

```
integer-Rle of length 200 with 159 runs
  Lengths: 12  1  1  1  2  1  1  1  1  2 ...  1  1  1  1  1  1  1  1  1
  Values :  0  1  0  2  0  1  0  1  0  1 ...  9 12  6  5 10  9  6  9 12
```

5.4 Finding Overlapping Ranges

The function `findOverlaps` detects overlaps between two *Ranges* objects.

```
> ol <- findOverlaps(ir, reduce(ir))
> as.matrix(ol)
```

```
     queryHits subjectHits
[1,]         1           1
[2,]         2           1
[3,]         3           1
[4,]         4           1
[5,]         5           1
[6,]         6           2
[7,]         7           3
```

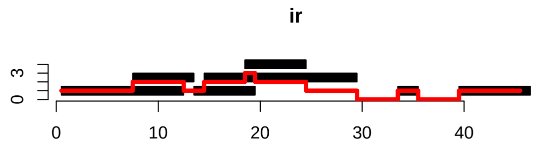

Figure 4: Plot of ranges with accumulated coverage.

5.5 Counting Overlapping Ranges

The function `coverage` counts the number of ranges over each position.

```
> cov <- coverage(ir)
> plotRanges(ir)
> cov <- as.vector(cov)
> mat <- cbind(seq_along(cov)-0.5, cov)
> d <- diff(cov) != 0
> mat <- rbind(cbind(mat[d,1]+1, mat[d,2]), mat)
> mat <- mat[order(mat[,1]),]
> lines(mat, col="red", lwd=4)
> axis(2)
```

5.6 Finding Neighboring Ranges

The `nearest` function finds the nearest neighbor ranges (overlapping is zero distance). The `precede` and `follow` functions find the non-overlapping nearest neighbors on a specific side.

5.7 Transforming Ranges

Utilities are available for transforming a *Ranges* object in a variety of ways. Some transformations, like `reduce` introduced above, can be dramatic, while others are simple per-range adjustments of the starts, ends or widths.

5.7.1 Adjusting starts, ends and widths

Perhaps the simplest transformation is to adjust the start values by a scalar offset, as performed by the `shift` function. Below, we shift all ranges forward 10 positions.

```
> shift(ir, 10)
```

```
IRanges of length 7
    start end width
[1]    11  22    12
[2]    18  23     6
[3]    24  29     6
[4]    25  39    15
[5]    29  34     6
[6]    44  45     2
[7]    50  56     7
```

The arithmetic functions +, - and * change both the start and the end/width by symmetrically expanding or contracting each range. Adding or subtracting a numeric (integer) vector to a *Ranges* causes each range to be expanded or contracted on each side by the corresponding value in the numeric vector.

```
> ir + seq_len(length(ir))

IRanges of length 7
    start end width
[1]     0  13    14
[2]     6  15    10
[3]    11  22    12
[4]    11  33    23
[5]    14  29    16
[6]    28  41    14
[7]    33  53    21
```

The * operator symmetrically magnifies a *Ranges* object by a factor, where positive contracts (zooms in) and negative expands (zooms out).

```
> ir * -2 # half the width

IRanges of length 7
    start end width
[1]    -5  18    24
[2]     5  16    12
[3]    11  22    12
[4]     7  36    30
[5]    16  27    12
[6]    33  36     4
[7]    36  49    14
```

There are several other ways to form subranges, besides symmetric contraction. These include `narrow`, `threebands` and `restrict`. `narrow` supports the adjustment of start, end and width values, which should be relative to each range. Unlike `shift`, these adjustments are vectorized over the ranges. As its name suggests, the ranges can only be narrowed.

```
> narrow(ir, start=1:5, width=2)

IRanges of length 7
    start end width
[1]     1   2     2
[2]     9  10     2
[3]    16  17     2
[4]    18  19     2
[5]    23  24     2
[6]    34  35     2
[7]    41  42     2
```

The `threebands` function extends `narrow` so that the remaining left and right regions adjacent to the narrowed region are also returned in separate *Ranges* objects.

```
> threebands(ir, start=1:5, width=2)
```

```
$left
IRanges of length 7
    start end width
[1]     1   0     0
[2]     8   8     1
[3]    14  15     2
[4]    15  17     3
[5]    19  22     4
[6]    34  33     0
[7]    40  40     1

$middle
IRanges of length 7
    start end width
[1]     1   2     2
[2]     9  10     2
[3]    16  17     2
[4]    18  19     2
[5]    23  24     2
[6]    34  35     2
[7]    41  42     2

$right
IRanges of length 7
    start end width
[1]     3  12    10
[2]    11  13     3
[3]    18  19     2
[4]    20  29    10
[5]    25  24     0
[6]    36  35     0
[7]    43  46     4
```

The `restrict` function ensures every range falls within a set of bounds. Ranges are contracted as necessary, and the ranges that fall completely outside of but not adjacent to the bounds are dropped, by default.

```
> restrict(ir, start=2, end=3)

IRanges of length 1
    start end width
[1]     2   3     2
```

5.7.2 Making ranges disjoint

A more complex type of operation is making a set of ranges disjoint, *i.e.* non-overlapping. For example, `threebands` returns a disjoint set of three ranges for each input range.

The `disjoin` function makes a *Ranges* object disjoint by fragmenting it into the widest ranges where the set of overlapping ranges is the same.

```
> disjoin(ir)
```

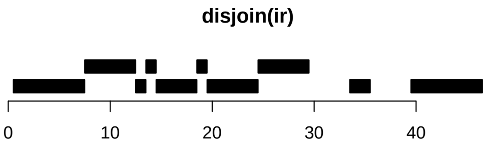

Figure 5: Plot of disjoined ranges.

```
IRanges of length 10
     start end width
[1]      1   7     7
[2]      8  12     5
[3]     13  13     1
[4]     14  14     1
[5]     15  18     4
[6]     19  19     1
[7]     20  24     5
[8]     25  29     5
[9]     34  35     2
[10]    40  46     7
```

```
> plotRanges(disjoin(ir))
```

A variant of `disjoin` is `disjointBins`, which divides the ranges into bins, such that the ranges in each bin are disjoint. The return value is an integer vector of the bins.

```
> disjointBins(ir)
```

```
[1] 1 2 1 2 3 1 1
```

5.7.3 Other transformations

Other transformations include `reflect` and `flank`. The former "flips" each range within a set of common reference bounds.

```
> reflect(ir, IRanges(start(ir), width=width(ir)*2))
```

```
IRanges of length 7
    start end width
[1]    13  24    12
[2]    14  19     6
[3]    20  25     6
[4]    30  44    15
[5]    25  30     6
[6]    36  37     2
[7]    47  53     7
```

The `flank` returns ranges of a specified width that flank, to the left (default) or right, each input range. One use case of this is forming promoter regions for a set of genes.

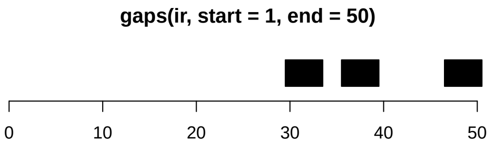

Figure 6: Plot of gaps from ranges.

```
> flank(ir, width = seq_len(length(ir)))

IRanges of length 7
    start end width
[1]     0   0     1
[2]     6   7     2
[3]    11  13     3
[4]    11  14     4
[5]    14  18     5
[6]    28  33     6
[7]    33  39     7
```

5.8 Set Operations

Sometimes, it is useful to consider a *Ranges* object as a set of integers, although there is always an implicit ordering. This is formalized by *NormalIRanges*, above, and we now present versions of the traditional mathematical set operations *complement*, *union*, *intersect*, and *difference* for *Ranges* objects. There are two variants for each operation. The first treats each *Ranges* object as a set and returns a *normal* value, while the other has a "parallel" semantic like `pmin`/`pmax` and performs the operation for each range pairing separately.

The *complement* operation is implemented by the `gaps` and `pgap` functions. By default, `gaps` will return the ranges that fall between the ranges in the (normalized) input. It is possible to specify a set of bounds, so that flanking ranges are included.

```
> gaps(ir, start=1, end=50)

IRanges of length 3
    start end width
[1]    30  33     4
[2]    36  39     4
[3]    47  50     4

> plotRanges(gaps(ir, start=1, end=50), c(1,50))
```

`pgap` considers each parallel pairing between two *Ranges* objects and finds the range, if any, between them. Note that the function name is singular, suggesting that only one range is returned per range in the input.

The remaining operations, *union*, *intersect* and *difference* are implemented by the `[p]union`, `[p]intersect` and `[p]setdiff` functions, respectively. These are relatively self-explanatory.

6 Vector Views

When we extract a sequence with `seqselect`, we can pass multiple ranges, each selecting a single consecutive subsequence. Those subsequences are extracted and concatenated into a single sequence. There are many cases where the user wishes to avoid the concatenation step and instead treat each consecutive subsequence as a separate element in a list.

While one could simply store each extracted sequence as an element in a list object like a *SimpleList*, this is undesirable for a couple of reasons. First, the user often wants to preserve the original sequence and declare a set of interesting regions as an overlay. This allows retrieving sequence values even after the ranges have been adjusted. Another benefit of an overlay approach is performance: the sequence values need not be copied.

For representing such an overlay, the *IRanges* package provides the virtual *Views* class, which derives from *IRanges* but also stores a sequence. Each range is said to represent a *view* onto the sequence.

Here, we will demonstrate the *RleViews* class, where the sequence is of class *Rle*. Other *Views* implementations exist, such as *XStringViews* in the *Biostrings* package.

6.1 Creating Views

There are two basic constructors for creating views: the `Views` function based on indicators and the `slice` based on numeric boundaries.

```
> xViews <- Views(xRle, xRle >= 1)
> xViews <- slice(xRle, 1)
> xViewsList <- slice(xRleList, 1)
```

6.2 Aggregating Views

While `sapply` can be used to loop over each window, the native functions `viewMaxs`, `viewMins`, `viewSums`, and `viewMeans` provide fast looping to calculate their respective statistical summaries.

```
> head(viewSums(xViews))

[1] 1 1 1 1 1 2

> viewSums(xViewsList)

IntegerList of length 2
[[1]] 1 1 1 1 1 2 1 1 2 3 1 6 1 3 4 ... 12 6 37 10 8 11 6 4 5 1 1 5 1 1
[[2]] 2 2 10 2 2 10 8 12 22 16 20 74 12 ... 2 12 2 6 4 2 2 4 2 2 2 2 2

> head(viewMaxs(xViews))

[1] 1 1 1 1 1 2

> viewMaxs(xViewsList)

IntegerList of length 2
[[1]] 1 1 1 1 1 2 1 1 1 2 1 2 1 2 3 1 ... 3 5 2 5 6 2 8 3 2 2 1 1 2 1 1
[[2]] 2 2 4 2 2 4 4 6 16 4 12 10 4 10 6 ... 4 2 4 2 4 2 2 2 4 2 2 2 2 2
```

7 Data on Ranges

When analyzing ranges, there are often additional variables of interest, besides the geometry (starts, ends and widths). To formally represent a dataset where the ranges are the observations, *IRanges* defines the *RangedData* class.

To create a *RangedData* instance, one needs to provide a *Ranges* object and, optionally, any number of variables on those ranges. The variable objects need not be vectors, but they must satisfy the contract of *DataFrame*.

```
> values <- rnorm(length(ir))
> rd <- RangedData(ir, name = letters[seq_len(length(ir))], values)
> rd
```

```
RangedData with 7 rows and 2 value columns across 1 space
     space    ranges |        name    values
  <factor> <IRanges> | <character> <numeric>
1        1  [ 1, 12] |           a 0.4359675
2        1  [ 8, 13] |           b 0.7279741
3        1  [14, 19] |           c 0.2617505
4        1  [15, 29] |           d 0.8157306
5        1  [19, 24] |           e 1.2115267
6        1  [34, 35] |           f 1.3045325
7        1  [40, 46] |           g -1.2268231
```

One might notice the term "sequence" in the above output. This refers to an important feature of *RangedData*: the ability to segregate ranges by their sequence (or space). For example, when analyzing genomic data, one is often working with ranges on different chromosomes. In many cases, such as when calculating overlap, one needs to separately treat ranges from different spaces, and *RangedData* aims to facilitate this mode of operation. The segregation may be performed at construction time.

```
> rd <- RangedData(ir, name = letters[seq_len(length(ir))], values,
+                  space = rep(c("chr1", "chr2"), c(3, length(ir) - 3)))
> rd
```

```
RangedData with 7 rows and 2 value columns across 2 spaces
     space    ranges |        name    values
  <factor> <IRanges> | <character> <numeric>
1     chr1  [ 1, 12] |           a 0.4359675
2     chr1  [ 8, 13] |           b 0.7279741
3     chr1  [14, 19] |           c 0.2617505
4     chr2  [15, 29] |           d 0.8157306
5     chr2  [19, 24] |           e 1.2115267
6     chr2  [34, 35] |           f 1.3045325
7     chr2  [40, 46] |           g -1.2268231
```

With the knowledge that the data is split into spaces, it should not be surprising that the `ranges` accessor returns a *RangesList* and `values` returns a *SplitDataFrameList*.

```
> ranges(rd)
```

```
IRangesList of length 2
$chr1
IRanges of length 3
```

```
    start end width
[1]     1  12    12
[2]     8  13     6
[3]    14  19     6

$chr2
IRanges of length 4
    start end width
[1]    15  29    15
[2]    19  24     6
[3]    34  35     2
[4]    40  46     7
```

```
> values(rd)
```

```
SplitDataFrameList of length 2
$chr1
DataFrame with 3 rows and 2 columns
         name    values
  <character> <numeric>
1           a 0.4359675
2           b 0.7279741
3           c 0.2617505

$chr2
DataFrame with 4 rows and 2 columns
         name     values
  <character>  <numeric>
1           d  0.8157306
2           e  1.2115267
3           f  1.3045325
4           g -1.2268231
```

To obtain a *RangedData* for a specific set of spaces, one should use the `[` function, which accepts logical, numeric and character indices.

```
> rd["chr1"]
```

```
RangedData with 3 rows and 2 value columns across 1 space
     space    ranges |        name    values
  <factor> <IRanges> | <character> <numeric>
1     chr1  [ 1, 12] |           a 0.4359675
2     chr1  [ 8, 13] |           b 0.7279741
3     chr1  [14, 19] |           c 0.2617505
```

```
> all(identical(rd["chr1"], rd[1]),
+     identical(rd[1], rd[c(TRUE, FALSE)]))
```

```
[1] TRUE
```

The `names` and `length` functions return the names and number of spaces, respectively.

```
> names(rd)
```

```
[1] "chr1" "chr2"
```

```
> length(rd)
```

```
[1] 2
```

The lapply function operates over the spaces. The object passed to the user function is a subset *RangedData*.

```
> lapply(rd, names)
```

```
$chr1
[1] "chr1"

$chr2
[1] "chr2"
```

The above would suggest that *RangedData* is a sequence of spaces. However, *RangedData* also inherits from *DataTable*, so it in some ways behaves like a sequence of columns. For example, one can extract a column via $ or [[.

```
> rd[[2]]
```

```
[1]  0.4359675  0.7279741  0.2617505  0.8157306  1.2115267  1.3045325
[7] -1.2268231
```

```
> rd$values
```

```
[1]  0.4359675  0.7279741  0.2617505  0.8157306  1.2115267  1.3045325
[7] -1.2268231
```

Note that the extracted columns are "unlisted" over the spaces, which is usually much more convenient than obtaining them as lists. It is important to note that the elements have been sorted by the space factor and thus may not have the same order as the objects passed to the constructor.

The two dimensional matrix-style subsetting is also supported. The rows are indexed globally, independent of space.

```
> rd[1:3, "name"]
```

```
RangedData with 3 rows and 1 value column across 2 spaces
     space    ranges |        name
  <factor> <IRanges> | <character>
1     chr1  [ 1, 12] |           a
2     chr1  [ 8, 13] |           b
3     chr1  [14, 19] |           c
```

8 IRanges in Biological Sequence Analysis

The *IRanges* packages was primarily designed with biological sequence analysis in mind and Table 1 shows how some biological sequence analysis concepts are represented in the *IRanges* class system.

Biological Entity	*Vector* Subclass
Genome browser track(s)	*RangedData*/*RangedDataList*
Coverage across chromosomes/contigs	*RleList*
Mapped ranges to genome	*CompressedIRangesList*
Data (sans ranges) across chroms/contigs	*SplitDataFrameList*

Table 1: *Vector* subclasses for Biological Sequence Analysis

```
> toLatex(sessionInfo())
```

- R version 3.0.0 (2013-04-03), `x86_64-unknown-linux-gnu`
- Locale: `LC_CTYPE=en_US.UTF-8`, `LC_NUMERIC=C`, `LC_TIME=en_US.UTF-8`, `LC_COLLATE=C`, `LC_MONETARY=en_US.UTF-8`, `LC_MESSAGES=en_US.UTF-8`, `LC_PAPER=C`, `LC_NAME=C`, `LC_ADDRESS=C`, `LC_TELEPHONE=C`, `LC_MEASUREMENT=en_US.UTF-8`, `LC_IDENTIFICATION=C`
- Base packages: base, datasets, grDevices, graphics, methods, parallel, stats, utils
- Other packages: BiocGenerics 0.6.0, IRanges 1.18.1
- Loaded via a namespace (and not attached): stats4 3.0.0, tools 3.0.0

Table 2: The output of `sessionInfo` on the build system after running this vignette.

9 Session Information
