# Supplementary material for: Software for Computing and Annotating Genomic Ranges
Source: PLoS Comput Biol. 2013 Aug 8;9(8):e1003118. doi: 10.1371/journal.pcbi.1003118 (PMC3738458; doi:10.1371/journal.pcbi.1003118)
Supplement: Software S1 — The IRanges package. The IRanges package provides efficient low-level and reusable S4 classes for storing and manipulating ranges of integers and compressed, genome-length vectors. (GZ) [file pcbi.1003118.s001.gz › IRanges/inst/doc/RleTricks.pdf]

Rle Tips and Tricks

Patrick Aboyoun

May 10, 2013

```
> rollmeanRle <- function (x, k)
+ {
+   n <- length(x)
+   cumsum(c(Rle(sum(window(x, 1, k))), window(x, k + 1, n) - window(x, 1, n - k))) / k
+ }

> rollvarRle <- function(x, k)
+ {
+   n <- length(x)
+   means <- rollmeanRle(x, k)
+   nextMean <- window(means, 2, n - k + 1)
+   cumsum(c(Rle(sum((window(x, 1, k) - means[1])^2)),
+   k * diff(means)^2 -
+   (window(x, 1, n - k) - nextMean)^2 +
+   (window(x, k + 1, n) - nextMean)^2)) / (k - 1)
+ }

> rollcovRle <- function(x, y, k)
+ {
+   n <- length(x)
+   meanX <- rollmeanRle(x, k)
+   meanY <- rollmeanRle(y, k)
+   nextMeanX <- window(meanX, 2, n - k + 1)
+   nextMeanY <- window(meanY, 2, n - k + 1)
+   cumsum(c(Rle(sum((window(x, 1, k) - meanX[1]) * (window(y, 1, k) - meanY[1]))),
+   k * diff(meanX) * diff(meanY) -
+   (window(x, 1, n - k) - nextMeanX) * (window(y, 1, n - k) - nextMeanY) +
+   (window(x, k + 1, n) - nextMeanX) * (window(y, k + 1, n) - nextMeanY))) / (k - 1)
+ }

> rollsdRle <- function(x, k)
+ {
+   sqrt(rollvarRle(x, k))
+ }

> rollcorRle <- function(x, y, k)
+ {
+   rollcovRle(x, y, k) / (rollsdRle(x, k) * rollsdRle(y, k))
+ }
```
