# Supplementary material for: Software for Computing and Annotating Genomic Ranges
Source: PLoS Comput Biol. 2013 Aug 8;9(8):e1003118. doi: 10.1371/journal.pcbi.1003118 (PMC3738458; doi:10.1371/journal.pcbi.1003118)
Supplement: Software S2 — The GenomicRanges package. The GenomicRanges package defines general purpose containers for storing genomic ranges as well as more specialized containers for storing alignments against a reference genome. (GZ) [file pcbi.1003118.s002.gz › GenomicRanges/inst/doc/GenomicRangesIntroduction.pdf]

An Introduction to Genomic Ranges Classes

Marc Carlson Patrick Aboyoun Hervé Pagès

April 16, 2013

Contents

1 Introduction **1**

2 *GRanges*: Single Interval Range Features **2**
2.1 Splitting and combining *GRanges* objects 4
2.2 Subsetting *GRanges* objects 5
2.3 Basic interval operations for *GRanges* objects 8
2.4 Interval set operations for *GRanges* objects 11

3 *GRangesList*: Multiple Interval Range Features **13**
3.1 Basic *GRangesList* accessors 13
3.2 Combining *GRangesList* objects 15
3.3 Basic interval operations for *GRangesList* objects 15
3.4 Subsetting *GRangesList* objects 16
3.5 Looping over *GRangesList* objects 19

4 Interval overlaps involving *GRanges* and *GRangesList* objects **22**

5 Gapped Alignments **23**
5.1 Load a 'BAM' file into a *GappedAlignments* object 23
5.2 Simple accessor methods 24
5.3 More accessor methods 25

1 Introduction

The *GenomicRanges* package serves as the foundation for representing genomic locations within the Bioconductor project. In the Bioconductor package hierarchy, it builds upon the *IRanges* (infrastructure) package and provides support for the *BSgenome* (infrastructure), *Rsamtools* (I/O), *ShortRead* (I/O & QA), *rtracklayer* (I/O), and *GenomicFeatures* (infrastructure) packages.

This package lays a foundation for genomic analysis by introducing three classes (*GRanges*, *GRangesList*, and *GappedAlignments*), which are used to represent single interval range features, multiple interval range features, and gapped alignments respectively. This vignette focuses on these classes and their associated methods.

The *GenomicRanges* package is available at bioconductor.org and can be downloaded via `biocLite`:

```
> source("http://bioconductor.org/biocLite.R")
> biocLite("GenomicRanges")
```

```
> library(GenomicRanges)
```

2 *GRanges*: Single Interval Range Features

The *GRanges* class represents a collection of genomic features that each have a single start and end location on the genome. This includes features such as contiguous binding sites, transcripts, and exons. These objects can be created by using the `GRanges` constructor function. For example,

```
> gr <-
+   GRanges(seqnames =
+           Rle(c("chr1", "chr2", "chr1", "chr3"), c(1, 3, 2, 4)),
+           ranges =
+           IRanges(1:10, end = 7:16, names = head(letters, 10)),
+           strand =
+           Rle(strand(c("-", "+", "*", "+", "-")),
+               c(1, 2, 2, 3, 2)),
+           score = 1:10,
+           GC = seq(1, 0, length=10))
> gr
```

```
GRanges with 10 ranges and 2 metadata columns:
    seqnames    ranges strand |     score                GC
       <Rle> <IRanges>  <Rle> | <integer>         <numeric>
  a     chr1  [ 1,  7]      - |         1                 1
  b     chr2  [ 2,  8]      + |         2 0.888888888888889
  c     chr2  [ 3,  9]      + |         3 0.777777777777778
  d     chr2  [ 4, 10]      * |         4 0.666666666666667
  e     chr1  [ 5, 11]      * |         5 0.555555555555556
  f     chr1  [ 6, 12]      + |         6 0.444444444444444
  g     chr3  [ 7, 13]      + |         7 0.333333333333333
  h     chr3  [ 8, 14]      + |         8 0.222222222222222
  i     chr3  [ 9, 15]      - |         9 0.111111111111111
  j     chr3  [10, 16]      - |        10                 0
  ---
  seqlengths:
   chr1 chr2 chr3
     NA   NA   NA
```

creates a *GRanges* object with 10 single interval features. The output of the *GRanges* `show` method separates the information into a left and right hand region that are separated by `|` symbols. The genomic coordinates (seqnames, ranges, and strand) are located on the left-hand side and the metadata columns (annotation) are located on the right. For this example, the metadata is comprised of `score` and `GC` information, but almost anything can be stored in the metadata portion of a *GRanges* object.

The components of the genomic coordinates within a *GRanges* object can be extracted using the `seqnames`, `ranges`, and `strand` accessor functions.

```
> seqnames(gr)
```

```
factor-Rle of length 10 with 4 runs
  Lengths:    1    3    2    4
  Values : chr1 chr2 chr1 chr3
Levels(3): chr1 chr2 chr3
```

```
> ranges(gr)
```

```
IRanges of length 10
     start end width names
[1]      1   7     7     a
[2]      2   8     7     b
[3]      3   9     7     c
[4]      4  10     7     d
[5]      5  11     7     e
[6]      6  12     7     f
[7]      7  13     7     g
[8]      8  14     7     h
[9]      9  15     7     i
[10]    10  16     7     j
```

```
> strand(gr)
```

```
factor-Rle of length 10 with 5 runs
  Lengths: 1 2 2 3 2
  Values : - + * + -
Levels(3): + - *
```

Stored annotations for these coordinates can be extracted as a *DataFrame* object using the `mcols` accessor.

```
> mcols(gr)
```

```
DataFrame with 10 rows and 2 columns
       score        GC
   <integer> <numeric>
1          1 1.0000000
2          2 0.8888889
3          3 0.7777778
4          4 0.6666667
5          5 0.5555556
6          6 0.4444444
7          7 0.3333333
8          8 0.2222222
9          9 0.1111111
10        10 0.0000000
```

```
> mcols(gr)$score
```

```
 [1]  1  2  3  4  5  6  7  8  9 10
```

Finally, the total lengths of the various sequences that the ranges are aligned to can also be stored in the *GRanges* object. So if this is data from *Homo sapiens*, we can set the values as:

```
> seqlengths(gr) <- c(249250621,243199373,198022430)
```

And then retrieves as:

```
> seqlengths(gr)
```

```
     chr1      chr2      chr3
249250621 243199373 198022430
```

Methods for accessing the `length` and `names` have also been defined.

```
> names(gr)

 [1] "a" "b" "c" "d" "e" "f" "g" "h" "i" "j"

> length(gr)

[1] 10
```

2.1 Splitting and combining *GRanges* objects

GRanges objects can be devided into groups using the `split` method. This produces a *GRangesList* object, a class that will be discussed in detail in the next section.

```
> sp <- split(gr, rep(1:2, each=5))
> sp

GRangesList of length 2:
$1
GRanges with 5 ranges and 2 metadata columns:
    seqnames    ranges strand |     score                GC
       <Rle> <IRanges>  <Rle> | <integer>         <numeric>
  a     chr1   [1,  7]      - |         1                 1
  b     chr2   [2,  8]      + |         2 0.888888888888889
  c     chr2   [3,  9]      + |         3 0.777777777777778
  d     chr2   [4, 10]      * |         4 0.666666666666667
  e     chr1   [5, 11]      * |         5 0.555555555555556

$2
GRanges with 5 ranges and 2 metadata columns:
    seqnames   ranges strand | score                GC
  f     chr1 [ 6, 12]      + |     6 0.444444444444444
  g     chr3 [ 7, 13]      + |     7 0.333333333333333
  h     chr3 [ 8, 14]      + |     8 0.222222222222222
  i     chr3 [ 9, 15]      - |     9 0.111111111111111
  j     chr3 [10, 16]      - |    10                 0

---
seqlengths:
      chr1      chr2      chr3
 249250621 243199373 198022430
```

If you then grab the components of this list, they can also be merged by using the `c` and `append` methods.

```
> c(sp[[1]], sp[[2]])

GRanges with 10 ranges and 2 metadata columns:
    seqnames    ranges strand |     score                GC
       <Rle> <IRanges>  <Rle> | <integer>         <numeric>
  a     chr1  [ 1,  7]      - |         1                 1
  b     chr2  [ 2,  8]      + |         2 0.888888888888889
  c     chr2  [ 3,  9]      + |         3 0.777777777777778
```

```
  d     chr2  [ 4, 10]      * |         4 0.666666666666667
  e     chr1  [ 5, 11]      * |         5 0.555555555555556
  f     chr1  [ 6, 12]      + |         6 0.444444444444444
  g     chr3  [ 7, 13]      + |         7 0.333333333333333
  h     chr3  [ 8, 14]      + |         8 0.222222222222222
  i     chr3  [ 9, 15]      - |         9 0.111111111111111
  j     chr3  [10, 16]      - |        10                 0
  ---
  seqlengths:
        chr1      chr2      chr3
   249250621 243199373 198022430
```

2.2 Subsetting *GRanges* objects

The expected subsetting operations are also available for *GRanges* objects.

```
> gr[2:3]

GRanges with 2 ranges and 2 metadata columns:
    seqnames    ranges strand |     score                GC
       <Rle> <IRanges>  <Rle> | <integer>         <numeric>
  b     chr2    [2, 8]      + |         2 0.888888888888889
  c     chr2    [3, 9]      + |         3 0.777777777777778
  ---
  seqlengths:
        chr1      chr2      chr3
   249250621 243199373 198022430
```

A second argument to the `[` subset operator can be used to specify which metadata columns to extract from the *GRanges* object. For example,

```
> gr[2:3, "GC"]

GRanges with 2 ranges and 1 metadata column:
    seqnames    ranges strand |                GC
       <Rle> <IRanges>  <Rle> |         <numeric>
  b     chr2    [2, 8]      + | 0.888888888888889
  c     chr2    [3, 9]      + | 0.777777777777778
  ---
  seqlengths:
        chr1      chr2      chr3
   249250621 243199373 198022430
```

You can also assign into elements of the *GRanges* object. Here is an example where the 2nd row of a *GRanges* object is replaced with the 1st row of `gr`.

```
> singles <- split(gr, names(gr))
> grMod <- gr
> grMod[2] <- singles[[1]]
> head(grMod, n=3)

GRanges with 3 ranges and 2 metadata columns:
    seqnames    ranges strand |     score                GC
```

```
       <Rle> <IRanges>  <Rle> | <integer>         <numeric>
  a     chr1    [1, 7]      - |         1                 1
  b     chr1    [1, 7]      - |         1                 1
  c     chr2    [3, 9]      + |         3 0.777777777777778
  ---
  seqlengths:
        chr1      chr2      chr3
   249250621 243199373 198022430
```

Here is a second example where the metadata for score from the 3rd element is replaced with the score from the 2nd row etc.

```
> grMod[2,1] <- singles[[3]][,1]
> head(grMod, n=3)

GRanges with 3 ranges and 2 metadata columns:
    seqnames    ranges strand |     score                GC
       <Rle> <IRanges>  <Rle> | <integer>         <numeric>
  a     chr1    [1, 7]      - |         1                 1
  b     chr2    [3, 9]      + |         3                 1
  c     chr2    [3, 9]      + |         3 0.777777777777778
  ---
  seqlengths:
        chr1      chr2      chr3
   249250621 243199373 198022430
```

There are also methods to repeat, reverse, or select specific portions of *GRanges* objects.

```
> rep(singles[[2]], times = 3)

GRanges with 3 ranges and 2 metadata columns:
    seqnames    ranges strand |     score                GC
       <Rle> <IRanges>  <Rle> | <integer>         <numeric>
  b     chr2    [2, 8]      + |         2 0.888888888888889
  b     chr2    [2, 8]      + |         2 0.888888888888889
  b     chr2    [2, 8]      + |         2 0.888888888888889
  ---
  seqlengths:
        chr1      chr2      chr3
   249250621 243199373 198022430

> rev(gr)

GRanges with 10 ranges and 2 metadata columns:
    seqnames    ranges strand |     score                GC
       <Rle> <IRanges>  <Rle> | <integer>         <numeric>
  j     chr3  [10, 16]      - |        10                 0
  i     chr3  [ 9, 15]      - |         9 0.111111111111111
  h     chr3  [ 8, 14]      + |         8 0.222222222222222
  g     chr3  [ 7, 13]      + |         7 0.333333333333333
  f     chr1  [ 6, 12]      + |         6 0.444444444444444
  e     chr1  [ 5, 11]      * |         5 0.555555555555556
  d     chr2  [ 4, 10]      * |         4 0.666666666666667
```

```
  c     chr2  [ 3,  9]      + |         3 0.777777777777778
  b     chr2  [ 2,  8]      + |         2 0.888888888888889
  a     chr1  [ 1,  7]      - |         1                 1
  ---
  seqlengths:
        chr1      chr2      chr3
   249250621 243199373 198022430
```

```
> head(gr,n=2)
```

```
GRanges with 2 ranges and 2 metadata columns:
    seqnames    ranges strand |     score                GC
       <Rle> <IRanges>  <Rle> | <integer>         <numeric>
  a     chr1    [1, 7]      - |         1                 1
  b     chr2    [2, 8]      + |         2 0.888888888888889
  ---
  seqlengths:
        chr1      chr2      chr3
   249250621 243199373 198022430
```

```
> tail(gr,n=2)
```

```
GRanges with 2 ranges and 2 metadata columns:
    seqnames    ranges strand |     score                GC
       <Rle> <IRanges>  <Rle> | <integer>         <numeric>
  i     chr3  [ 9, 15]      - |         9 0.111111111111111
  j     chr3  [10, 16]      - |        10                 0
  ---
  seqlengths:
        chr1      chr2      chr3
   249250621 243199373 198022430
```

```
> window(gr, start=2,end=4)
```

```
GRanges with 3 ranges and 2 metadata columns:
    seqnames    ranges strand |     score                GC
       <Rle> <IRanges>  <Rle> | <integer>         <numeric>
  b     chr2   [2,  8]      + |         2 0.888888888888889
  c     chr2   [3,  9]      + |         3 0.777777777777778
  d     chr2   [4, 10]      * |         4 0.666666666666667
  ---
  seqlengths:
        chr1      chr2      chr3
   249250621 243199373 198022430
```

```
> seqselect(gr, start=c(2,7), end=c(3,9))
```

```
GRanges with 5 ranges and 2 metadata columns:
    seqnames    ranges strand |     score                GC
       <Rle> <IRanges>  <Rle> | <integer>         <numeric>
  b     chr2   [2,  8]      + |         2 0.888888888888889
  c     chr2   [3,  9]      + |         3 0.777777777777778
  g     chr3   [7, 13]      + |         7 0.333333333333333
```

```
  h     chr3   [8, 14]      + |         8 0.222222222222222
  i     chr3   [9, 15]      - |         9 0.111111111111111
  ---
  seqlengths:
        chr1      chr2      chr3
   249250621 243199373 198022430
```

2.3 Basic interval operations for *GRanges* objects

Basic interval characteristics of *GRanges* objects can be extracted using the `start`, `end`, `width`, and `range` methods.

```
> g <- gr[1:3]
> g <- append(g, singles[[10]])
> start(g)

[1]  1  2  3 10

> end(g)

[1]  7  8  9 16

> width(g)

[1] 7 7 7 7

> range(g)

GRanges with 3 ranges and 0 metadata columns:
      seqnames    ranges strand
         <Rle> <IRanges>  <Rle>
  [1]     chr1  [ 1,  7]      -
  [2]     chr2  [ 2,  9]      +
  [3]     chr3  [10, 16]      -
  ---
  seqlengths:
        chr1      chr2      chr3
   249250621 243199373 198022430
```

The *GRanges* class also has many methods for manipulating the intervals. For example, the `flank` method can be used to recover regions flanking the set of ranges represented by the *GRanges* object. So to get a *GRanges* object containing the ranges that include the 10 bases upstream of the ranges:

```
> flank(g, 10)

GRanges with 4 ranges and 2 metadata columns:
    seqnames    ranges strand |     score                GC
       <Rle> <IRanges>  <Rle> | <integer>         <numeric>
  a     chr1  [ 8, 17]      - |         1                 1
  b     chr2  [ 1,  1]      + |         2 0.888888888888889
  c     chr2  [ 1,  2]      + |         3 0.777777777777778
  j     chr3  [17, 26]      - |        10                 0
  ---
  seqlengths:
        chr1      chr2      chr3
   249250621 243199373 198022430
```

And to include the downstream bases:

```
> flank(g, 10, start=FALSE)

GRanges with 4 ranges and 2 metadata columns:
    seqnames    ranges strand |     score                GC
       <Rle> <IRanges>  <Rle> | <integer>         <numeric>
  a     chr1  [ 1,  0]      - |         1                 1
  b     chr2  [ 9, 18]      + |         2 0.888888888888889
  c     chr2  [10, 19]      + |         3 0.777777777777778
  j     chr3  [ 1,  9]      - |        10                 0
  ---
  seqlengths:
        chr1      chr2      chr3
   249250621 243199373 198022430
```

Similar to `flank`, there are also operations to `resize` and `shift` our *GRanges* object. The `shift` method will move the ranges by a specific number of base pairs, and the `resize` method will extend the ranges by a specified width.

```
> shift(g, 5)

GRanges with 4 ranges and 2 metadata columns:
    seqnames    ranges strand |     score                GC
       <Rle> <IRanges>  <Rle> | <integer>         <numeric>
  a     chr1  [ 6, 12]      - |         1                 1
  b     chr2  [ 7, 13]      + |         2 0.888888888888889
  c     chr2  [ 8, 14]      + |         3 0.777777777777778
  j     chr3  [15, 21]      - |        10                 0
  ---
  seqlengths:
        chr1      chr2      chr3
   249250621 243199373 198022430

> resize(g, 30)

GRanges with 4 ranges and 2 metadata columns:
    seqnames    ranges strand |     score                GC
       <Rle> <IRanges>  <Rle> | <integer>         <numeric>
  a     chr1   [1,  7]      - |         1                 1
  b     chr2   [2, 31]      + |         2 0.888888888888889
  c     chr2   [3, 32]      + |         3 0.777777777777778
  j     chr3   [1, 16]      - |        10                 0
  ---
  seqlengths:
        chr1      chr2      chr3
   249250621 243199373 198022430
```

The `reduce` will align the ranges and merge overlapping ranges to produce a simplified set.

```
> reduce(g)
```

```
GRanges with 3 ranges and 0 metadata columns:
      seqnames    ranges strand
         <Rle> <IRanges>  <Rle>
  [1]     chr1  [ 1,  7]      -
  [2]     chr2  [ 2,  9]      +
  [3]     chr3  [10, 16]      -
  ---
  seqlengths:
        chr1      chr2      chr3
   249250621 243199373 198022430
```

Sometimes you may be interested in the spaces or the qualities of the spaces between the ranges represented by your *GRanges* object. The `gaps` method will help you calculate the spaces between a reduced version of your ranges:

```
> gaps(g)

GRanges with 11 ranges and 0 metadata columns:
       seqnames          ranges strand
          <Rle>       <IRanges>  <Rle>
   [1]     chr1 [ 1, 249250621]      +
   [2]     chr1 [ 8, 249250621]      -
   [3]     chr1 [ 1, 249250621]      *
   [4]     chr2 [ 1,         1]      +
   [5]     chr2 [10, 243199373]      +
   ...      ...             ...    ...
   [7]     chr2 [ 1, 243199373]      *
   [8]     chr3 [ 1, 198022430]      +
   [9]     chr3 [ 1,         9]      -
  [10]     chr3 [17, 198022430]      -
  [11]     chr3 [ 1, 198022430]      *
  ---
  seqlengths:
        chr1      chr2      chr3
   249250621 243199373 198022430
```

And sometimes you also may want to know how many quantitatively unique fragments your ranges could possibly represent. For this task there is the `disjoin` method.

```
> disjoin(g)

GRanges with 5 ranges and 0 metadata columns:
      seqnames    ranges strand
         <Rle> <IRanges>  <Rle>
  [1]     chr1  [ 1,  7]      -
  [2]     chr2  [ 2,  2]      +
  [3]     chr2  [ 3,  8]      +
  [4]     chr2  [ 9,  9]      +
  [5]     chr3  [10, 16]      -
  ---
  seqlengths:
        chr1      chr2      chr3
   249250621 243199373 198022430
```

One of the most powerful methods for looking at *GRanges* objects is the `coverage` method. The `coverage` method quantifies the degree of overlap for all the ranges in a *GRanges* object.

```
> coverage(g)

RleList of length 3
$chr1
integer-Rle of length 249250621 with 2 runs
  Lengths:         7 249250614
  Values :         1         0

$chr2
integer-Rle of length 243199373 with 5 runs
  Lengths:         1         1         6         1 243199364
  Values :         0         1         2         1         0

$chr3
integer-Rle of length 198022430 with 3 runs
  Lengths:         9         7 198022414
  Values :         0         1         0
```

2.4 Interval set operations for *GRanges* objects

There are also operations for calculating relationships between different *GRanges* objects. Here are a some examples for how you can calculate the `union`, the `intersect` and the asymmetric difference (using `setdiff`).

```
> g2 <- head(gr, n=2)
> union(g, g2)

GRanges with 3 ranges and 0 metadata columns:
      seqnames    ranges strand
         <Rle> <IRanges>  <Rle>
  [1]     chr1  [ 1,  7]      -
  [2]     chr2  [ 2,  9]      +
  [3]     chr3  [10, 16]      -
  ---
  seqlengths:
        chr1      chr2      chr3
   249250621 243199373 198022430

> intersect(g, g2)

GRanges with 2 ranges and 0 metadata columns:
      seqnames    ranges strand
         <Rle> <IRanges>  <Rle>
  [1]     chr1    [1, 7]      -
  [2]     chr2    [2, 8]      +
  ---
  seqlengths:
        chr1      chr2      chr3
   249250621 243199373 198022430

> setdiff(g, g2)
```

```
GRanges with 2 ranges and 0 metadata columns:
      seqnames    ranges strand
         <Rle> <IRanges>  <Rle>
  [1]     chr2  [ 9,  9]      +
  [2]     chr3  [10, 16]      -
  ---
  seqlengths:
        chr1      chr2      chr3
   249250621 243199373 198022430
```

In addition, there is similar set of operations that act at the level of the individual ranges within each *GRanges*. These operations all begin with a "p", which is short for parallel. A requirement for this set of operations is that the number of elements in each *GRanges* object has to be the same, and that both of the objects have to have the same seqnames and strand assignments throughout.

```
> g3 <- g[1:2]
> ranges(g3[1]) <- IRanges(start=5, end=12)
> punion(g2, g3)
```

```
GRanges with 2 ranges and 0 metadata columns:
    seqnames    ranges strand
       <Rle> <IRanges>  <Rle>
  a     chr1   [1, 12]      -
  b     chr2   [2,  8]      +
  ---
  seqlengths:
        chr1      chr2      chr3
   249250621 243199373 198022430
```

```
> pintersect(g2, g3)
```

```
GRanges with 2 ranges and 0 metadata columns:
    seqnames    ranges strand
       <Rle> <IRanges>  <Rle>
  a     chr1    [5, 7]      -
  b     chr2    [2, 8]      +
  ---
  seqlengths:
        chr1      chr2      chr3
   249250621 243199373 198022430
```

```
> psetdiff(g2, g3)
```

```
GRanges with 2 ranges and 0 metadata columns:
    seqnames    ranges strand
       <Rle> <IRanges>  <Rle>
  a     chr1    [1, 4]      -
  b     chr2    [2, 1]      +
  ---
  seqlengths:
        chr1      chr2      chr3
   249250621 243199373 198022430
```

For even more information on the `GRanges` classes be sure to consult the manual page.

```
> ?GRanges
```

3 *GRangesList*: Multiple Interval Range Features

Some important genomic features, such as spliced transcripts that are are comprised of exons, are inherently compound structures. Such a feature makes much more sense when expressed as a compound object such as a *GRangesList*. Whenever genomic features consist of multiple ranges that are grouped by a parent feature, they can be represented as *GRangesList* object. Consider the simple example of the two transcript `GRangesList` below created using the `GRangesList` constructor.

```
> gr1 <-
+   GRanges(seqnames = "chr2", ranges = IRanges(3, 6),
+           strand = "+", score = 5L, GC = 0.45)
> gr2 <-
+   GRanges(seqnames = c("chr1", "chr1"),
+           ranges = IRanges(c(7,13), width = 3),
+           strand = c("+", "-"), score = 3:4, GC = c(0.3, 0.5))
> grl <- GRangesList("txA" = gr1, "txB" = gr2)
> grl
```

```
GRangesList of length 2:
$txA
GRanges with 1 range and 2 metadata columns:
      seqnames    ranges strand |     score        GC
         <Rle> <IRanges>  <Rle> | <integer> <numeric>
  [1]     chr2    [3, 6]      + |         5      0.45

$txB
GRanges with 2 ranges and 2 metadata columns:
      seqnames   ranges strand | score  GC
  [1]     chr1 [ 7,  9]      + |     3 0.3
  [2]     chr1 [13, 15]      - |     4 0.5

---
seqlengths:
 chr2 chr1
   NA   NA
```

The `show` method for a *GRangesList* object displays it as a named list of *GRanges* objects, where the names of this list are considered to be the names of the grouping feature. In the example above, the groups of individual exon ranges are represented as separate *GRanges* objects which are further organized into a list structure where each element name is a transcript name. Many other combinations of grouped and labeled *GRanges* objects are possible of course, but this example is expected to be a common arrangement.

3.1 Basic *GRangesList* accessors

Just as with *GRanges* object, the components of the genomic coordinates within a *GRangesList* object can be extracted using simple accessor methods. Not surprisingly, the *GRangesList* objects have many of the same accessors as *GRanges* objects. The difference is that many of these methods return a list since the input is now essentially a list of *GRanges* objects. Here are a few examples:

```
> seqnames(grl)
```

```
RleList of length 2
$txA
```

```
factor-Rle of length 1 with 1 run
  Lengths:    1
  Values : chr2
Levels(2): chr2 chr1

$txB
factor-Rle of length 2 with 1 run
  Lengths:    2
  Values : chr1
Levels(2): chr2 chr1
```

```
> ranges(grl)
```

```
IRangesList of length 2
$txA
IRanges of length 1
    start end width
[1]     3   6     4

$txB
IRanges of length 2
    start end width
[1]     7   9     3
[2]    13  15     3
```

```
> strand(grl)
```

```
RleList of length 2
$txA
factor-Rle of length 1 with 1 run
  Lengths: 1
  Values : +
Levels(3): + - *

$txB
factor-Rle of length 2 with 2 runs
  Lengths: 1 1
  Values : + -
Levels(3): + - *
```

The `length` and `names` methods will return the length or names of the list and the `seqlengths` method will return the set of sequence lengths.

```
> length(grl)
```

```
[1] 2
```

```
> names(grl)
```

```
[1] "txA" "txB"
```

```
> seqlengths(grl)
```

```
chr2 chr1
  NA   NA
```

The `elementLengths` method returns a list of integers corresponding to the result of calling `length` on each individual *GRanges* object contained by the *GRangesList*. This is a faster alternative to calling `lapply` on the *GRangesList*.

```
> elementLengths(grl)

txA txB
  1   2
```

You can also use `isEmpty` to test if a *GRangesList* object contains anything.

```
> isEmpty(grl)

[1] FALSE
```

Finally, in the context of a *GRangesList* object, the `mcols` method performs a similar operation to what it does on a *GRanges* object. However, this metadata now refers to information at the list level instead of the level of the individual *GRanges* objects.

```
> mcols(grl) <- c("Transcript A","Transcript B")
> mcols(grl)

DataFrame with 2 rows and 1 column
         value
   <character>
1 Transcript A
2 Transcript B
```

3.2 Combining *GRangesList* objects

GRangesList objects can be unlisted to combine the separate *GRanges* objects that they contain as an expanded *GRanges*.

```
> ul <- unlist(grl)
```

You can also append values together useing `append` or `c`.

3.3 Basic interval operations for *GRangesList* objects

For interval operations, many of the same methods exist for *GRangesList* objects that exist for *GRanges* objects.

```
> start(grl)

IntegerList of length 2
[["txA"]] 3
[["txB"]] 7 13
```

```
> end(grl)

IntegerList of length 2
[["txA"]] 6
[["txB"]] 9 15
```

```
> width(grl)

IntegerList of length 2
[["txA"]] 4
[["txB"]] 3 3
```

And as with *GRanges* objects, you can also shift all the *GRanges* objects in a *GRangesList* object, or calculate the coverage. Both of these operations are also carried out across each *GRanges* list member.

```
> shift(grl, 20)

GRangesList of length 2:
$txA
GRanges with 1 range and 2 metadata columns:
      seqnames    ranges strand |     score        GC
         <Rle> <IRanges>  <Rle> | <integer> <numeric>
  [1]     chr2  [23, 26]      + |         5      0.45

$txB
GRanges with 2 ranges and 2 metadata columns:
      seqnames   ranges strand | score  GC
  [1]     chr1 [27, 29]      + |     3 0.3
  [2]     chr1 [33, 35]      - |     4 0.5

---
seqlengths:
 chr2 chr1
   NA   NA

> coverage(grl)

RleList of length 2
$chr2
integer-Rle of length 6 with 2 runs
  Lengths: 2 4
  Values : 0 1

$chr1
integer-Rle of length 15 with 4 runs
  Lengths: 6 3 3 3
  Values : 0 1 0 1
```

3.4 Subsetting *GRangesList* objects

As you might guess, the subsetting of a *GRangesList* object is quite different from subsetting on a *GRanges* object in that it acts as if you are subseting a list. If you try out the following you will notice that the standard conventions have been followed.

```
> grl[1]
> grl[[1]]
> grl["txA"]
> grl$txB
```

But in addition to this, when subsetting a *GRangesList*, you can also pass in a second parameter (as with a *GRanges* object) to again specify which of the metadata columns you wish to select.

```
> grl[1, "score"]

GRangesList of length 1:
$txA
GRanges with 1 range and 1 metadata column:
      seqnames    ranges strand |     score
         <Rle> <IRanges>  <Rle> | <integer>
  [1]     chr2    [3, 6]      + |         5

---
seqlengths:
 chr2 chr1
   NA   NA

> grl["txB", "GC"]

GRangesList of length 1:
$txB
GRanges with 2 ranges and 1 metadata column:
      seqnames    ranges strand |        GC
         <Rle> <IRanges>  <Rle> | <numeric>
  [1]     chr1  [ 7,  9]      + |       0.3
  [2]     chr1  [13, 15]      - |       0.5

---
seqlengths:
 chr2 chr1
   NA   NA
```

The `head`, `tail`, `rep`, `rev`, `window` and `seqselect` methods all behave as you would expect them to for a list object. For example, the elements referred to by `window` or `seqselect` are now list elements instead of *GRanges* elements.

```
> rep(grl[[1]], times = 3)

GRanges with 3 ranges and 2 metadata columns:
      seqnames    ranges strand |     score        GC
         <Rle> <IRanges>  <Rle> | <integer> <numeric>
  [1]     chr2    [3, 6]      + |         5      0.45
  [2]     chr2    [3, 6]      + |         5      0.45
  [3]     chr2    [3, 6]      + |         5      0.45
  ---
  seqlengths:
   chr2 chr1
     NA   NA

> rev(grl)

GRangesList of length 2:
$txB
```

```
GRanges with 2 ranges and 2 metadata columns:
      seqnames    ranges strand |     score        GC
         <Rle> <IRanges>  <Rle> | <integer> <numeric>
  [1]     chr1  [ 7,  9]      + |         3       0.3
  [2]     chr1  [13, 15]      - |         4       0.5

$txA
GRanges with 1 range and 2 metadata columns:
      seqnames ranges strand | score   GC
  [1]     chr2 [3, 6]      + |     5 0.45

---
seqlengths:
 chr2 chr1
   NA   NA
```

```
> head(grl, n=1)
```

```
GRangesList of length 1:
$txA
GRanges with 1 range and 2 metadata columns:
      seqnames    ranges strand |     score        GC
         <Rle> <IRanges>  <Rle> | <integer> <numeric>
  [1]     chr2    [3, 6]      + |         5      0.45

---
seqlengths:
 chr2 chr1
   NA   NA
```

```
> tail(grl, n=1)
```

```
GRangesList of length 1:
$txB
GRanges with 2 ranges and 2 metadata columns:
      seqnames    ranges strand |     score        GC
         <Rle> <IRanges>  <Rle> | <integer> <numeric>
  [1]     chr1  [ 7,  9]      + |         3       0.3
  [2]     chr1  [13, 15]      - |         4       0.5

---
seqlengths:
 chr2 chr1
   NA   NA
```

```
> window(grl, start=1, end=1)
```

```
GRangesList of length 1:
$txA
GRanges with 1 range and 2 metadata columns:
      seqnames    ranges strand |     score        GC
         <Rle> <IRanges>  <Rle> | <integer> <numeric>
```

```
  [1]     chr2    [3, 6]      + |         5      0.45

---
seqlengths:
 chr2 chr1
   NA   NA

> seqselect(grl, start=2, end=2)

GRangesList of length 1:
$txB
GRanges with 2 ranges and 2 metadata columns:
      seqnames    ranges strand |     score        GC
         <Rle> <IRanges>  <Rle> | <integer> <numeric>
  [1]     chr1  [ 7,  9]      + |         3       0.3
  [2]     chr1  [13, 15]      - |         4       0.5

---
seqlengths:
 chr2 chr1
   NA   NA
```

3.5 Looping over *GRangesList* objects

For *GRangesList* objects there is also a family of `apply` methods. These include `lapply`, `sapply`, `mapply`, `endoapply`, `mendoapply`, `Map`, and `Reduce`.

The different looping methods defined for *GRangesList* objects are useful for returning different kinds of results. The standard `lapply` and `sapply` behave according to convention, with the `lapply` method returning a list and `sapply` returning a more simplified output.

```
> lapply(grl, length)

$txA
[1] 1

$txB
[1] 2

> sapply(grl, length)

txA txB
  1   2
```

As with *IRanges* objects, there is also a multivariate version of `sapply`, called `mapply`, defined for *GRangesList* objects. And, if you don't want the results simplified, you can call the `Map` method, which does the same things as `mapply` but without simplifying the output.

```
> grl2 <- shift(grl, 10)
> names(grl2) <- c("shiftTxA", "shiftTxB")
> mapply(c, grl, grl2)

$txA
GRanges with 2 ranges and 2 metadata columns:
```

```
      seqnames    ranges strand |     score        GC
         <Rle> <IRanges>  <Rle> | <integer> <numeric>
  [1]     chr2  [ 3,  6]      + |         5      0.45
  [2]     chr2  [13, 16]      + |         5      0.45
  ---
  seqlengths:
   chr2 chr1
     NA   NA

$txB
GRanges with 4 ranges and 2 metadata columns:
      seqnames    ranges strand |     score        GC
         <Rle> <IRanges>  <Rle> | <integer> <numeric>
  [1]     chr1  [ 7,  9]      + |         3       0.3
  [2]     chr1  [13, 15]      - |         4       0.5
  [3]     chr1  [17, 19]      + |         3       0.3
  [4]     chr1  [23, 25]      - |         4       0.5
  ---
  seqlengths:
   chr2 chr1
     NA   NA
```

```
> Map(c, grl, grl2)
```

```
$txA
GRanges with 2 ranges and 2 metadata columns:
      seqnames    ranges strand |     score        GC
         <Rle> <IRanges>  <Rle> | <integer> <numeric>
  [1]     chr2  [ 3,  6]      + |         5      0.45
  [2]     chr2  [13, 16]      + |         5      0.45
  ---
  seqlengths:
   chr2 chr1
     NA   NA

$txB
GRanges with 4 ranges and 2 metadata columns:
      seqnames    ranges strand |     score        GC
         <Rle> <IRanges>  <Rle> | <integer> <numeric>
  [1]     chr1  [ 7,  9]      + |         3       0.3
  [2]     chr1  [13, 15]      - |         4       0.5
  [3]     chr1  [17, 19]      + |         3       0.3
  [4]     chr1  [23, 25]      - |         4       0.5
  ---
  seqlengths:
   chr2 chr1
     NA   NA
```

Sometimes, you may not want to get back a simplified output or a list. Sometimes you will want to get back a modified version of the *GRangesList* that you originally passed in. This is conceptually similar to the mathematical notion of an endomorphism. This is achieved using the `endoapply` method, which will return the results as a *GRangesList* object.

```
> endoapply(grl,rev)

GRangesList of length 2:
$txA
GRanges with 1 range and 2 metadata columns:
      seqnames    ranges strand |     score        GC
         <Rle> <IRanges>  <Rle> | <integer> <numeric>
  [1]     chr2    [3, 6]      + |         5      0.45

$txB
GRanges with 2 ranges and 2 metadata columns:
      seqnames   ranges strand | score  GC
  [1]     chr1 [13, 15]      - |     4 0.5
  [2]     chr1 [ 7,  9]      + |     3 0.3

---
seqlengths:
 chr2 chr1
   NA   NA
```

And, there is also a multivariate version of the `endoapply` method in the form of the `mendoapply` method.

```
> mendoapply(c,grl,grl2)

GRangesList of length 2:
$txA
GRanges with 2 ranges and 2 metadata columns:
      seqnames    ranges strand |     score        GC
         <Rle> <IRanges>  <Rle> | <integer> <numeric>
  [1]     chr2  [ 3,  6]      + |         5      0.45
  [2]     chr2  [13, 16]      + |         5      0.45

$txB
GRanges with 4 ranges and 2 metadata columns:
      seqnames   ranges strand | score  GC
  [1]     chr1 [ 7,  9]      + |     3 0.3
  [2]     chr1 [13, 15]      - |     4 0.5
  [3]     chr1 [17, 19]      + |     3 0.3
  [4]     chr1 [23, 25]      - |     4 0.5

---
seqlengths:
 chr2 chr1
   NA   NA
```

Finally, the `Reduce` method will allow the *GRanges* objects to be collapsed across the whole of the *GRangesList* object.

```
> Reduce(c,grl)

GRanges with 3 ranges and 2 metadata columns:
      seqnames    ranges strand |     score        GC
         <Rle> <IRanges>  <Rle> | <integer> <numeric>
```

```
  [1]     chr2  [ 3,  6]      + |         5      0.45
  [2]     chr1  [ 7,  9]      + |         3       0.3
  [3]     chr1  [13, 15]      - |         4       0.5
  ---
  seqlengths:
   chr2 chr1
     NA   NA
```

For even more information on the `GRangesList` classes be sure to consult the manual page.

```
> ?GRangesList
```

4 Interval overlaps involving *GRanges* and *GRangesList* objects

Interval overlapping is the process of comparing the ranges in two objects to determine if and when they overlap. As such, it is perhaps the most common operation performed on *GRanges* and *GRangesList* objects. To this end, the *GenomicRanges* package provides a family of interval overlap functions. The most general of these functions is `findOverlaps`, which takes a query and a subject as inputs and returns a *Hits* object containing the index pairings for the overlapping elements.

```
> mtch <- findOverlaps(gr, grl)
> as.matrix(mtch)

     queryHits subjectHits
[1,]         2           1
[2,]         3           1
[3,]         4           1
[4,]         5           2
[5,]         6           2
```

As suggested in the sections discussing the nature of the *GRanges* and *GRangesList* classes, the index in the above matrix of hits for a *GRanges* object is a single range while for a *GRangesList* object it is the set of ranges that define a "feature".

Another function in the overlaps family is `countOverlaps`, which tabulates the number of overlaps for each element in the query.

```
> countOverlaps(gr, grl)

a b c d e f g h i j
0 1 1 1 1 1 0 0 0 0
```

A third function in this family is `subsetByOverlaps`, which extracts the elements in the query that overlap at least one element in the subject.

```
> subsetByOverlaps(gr,grl)

GRanges with 5 ranges and 2 metadata columns:
    seqnames    ranges strand |     score                GC
       <Rle> <IRanges>  <Rle> | <integer>         <numeric>
  b     chr2   [2,  8]      + |         2 0.888888888888889
  c     chr2   [3,  9]      + |         3 0.777777777777778
  d     chr2   [4, 10]      * |         4 0.666666666666667
  e     chr1   [5, 11]      * |         5 0.555555555555556
```

```
  f     chr1   [6, 12]      + |         6 0.444444444444444
  ---
  seqlengths:
        chr1      chr2      chr3
   249250621 243199373 198022430
```

Finally, you can use the select argument to get the index of the first overlapping element in the subject for each element in the query.

```
> findOverlaps(gr, grl, select="first")

 [1] NA  1  1  1  2  2 NA NA NA NA

> findOverlaps(grl, gr, select="first")

[1] 2 5
```

5 Gapped Alignments

In addition to *GRanges* and *GRangesList* classes, the *GenomicRanges* package defines the *GappedAlignments* class, which is a more specialized container for storing a set of alignments. The class is intended to support alignments in general, not only those coming from a 'Binary Alignment Map' or 'BAM' files. Also alignments with gaps in the reference sequence (a.k.a. *gapped alignments*) are supported which, for example, makes the class suited for storing junction reads from an RNA-seq experiment.

More precisely, a *GappedAlignments* object is a vector-like object where each element describes an *alignment*, that is, how a given sequence (called *query* or *read*, typically short) aligns to a reference sequence (typically long).

As shown later in this document, a *GappedAlignments* object can be created from a 'BAM' file. In that case, each element in the resulting object will correspond to a record in the file. One important thing to note though is that not all the information present in the BAM/SAM records is stored in the object. In particular, for now, we discard the query sequences (SEQ field), the query ids (QNAME field), the query qualities (QUAL), the mapping qualities (MAPQ) and any other information that is not needed in order to support the basic set of operations described in this document. This also means that multi-reads (i.e. reads with multiple hits in the reference) don't receive any special treatment i.e. the various SAM/BAM records corresponding to a multi-read will show up in the GappedAlignments object as if they were coming from different/unrelated queries. Also paired-end reads will be treated as single-end reads and the pairing information will be lost. This might change in the future.

5.1 Load a 'BAM' file into a *GappedAlignments* object

First we use the readGappedAlignments function to load a toy 'BAM' file into a *GappedAlignments* object:

```
> library(Rsamtools)
> aln1_file <- system.file("extdata", "ex1.bam", package="Rsamtools")
> aln1 <- readGappedAlignments(aln1_file)
> aln1

GappedAlignments with 3271 alignments and 0 metadata columns:
         seqnames strand       cigar    qwidth     start       end
            <Rle>  <Rle> <character> <integer> <integer> <integer>
     [1]     seq1      +         36M        36         1        36
     [2]     seq1      +         35M        35         3        37
```

```
    [3]     seq1      +         35M         35          5         39
    [4]     seq1      +         36M         36          6         41
    [5]     seq1      +         35M         35          9         43
    ...      ...    ...         ...        ...        ...        ...
 [3267]     seq2      +         35M         35       1524       1558
 [3268]     seq2      +         35M         35       1524       1558
 [3269]     seq2      -         35M         35       1528       1562
 [3270]     seq2      -         35M         35       1532       1566
 [3271]     seq2      -         35M         35       1533       1567
            width      ngap
        <integer> <integer>
    [1]        36         0
    [2]        35         0
    [3]        35         0
    [4]        36         0
    [5]        35         0
    ...       ...       ...
 [3267]        35         0
 [3268]        35         0
 [3269]        35         0
 [3270]        35         0
 [3271]        35         0
 ---
 seqlengths:
  seq1 seq2
  1575 1584
```

```
> length(aln1)
```

```
[1] 3271
```

3271 ‘BAM’ records were loaded into the object.

Note that `readGappedAlignments` would have discarded any ‘BAM’ record describing an unaligned query (see description of the <flag> field in the SAM Format Specification [1] for more information). The reader interested in tracking down these events can always use the `scanBam` function but this goes beyond the scope of this document.

5.2 Simple accessor methods

There is one accessor per field displayed by the `show` method and it has the same name as the field. All of them return a vector or factor of the same length as the object:

```
> head(seqnames(aln1))
```

```
factor-Rle of length 6 with 1 run
  Lengths:    6
  Values : seq1
Levels(2): seq1 seq2
```

```
> seqlevels(aln1)
```

```
[1] "seq1" "seq2"
```

[1] http://samtools.sourceforge.net/SAM1.pdf

```
> head(strand(aln1))

factor-Rle of length 6 with 1 run
  Lengths: 6
  Values : +
Levels(3): + - *

> head(cigar(aln1))

[1] "36M" "35M" "35M" "36M" "35M" "35M"

> head(qwidth(aln1))

[1] 36 35 35 36 35 35

> head(start(aln1))

[1]  1  3  5  6  9 13

> head(end(aln1))

[1] 36 37 39 41 43 47

> head(width(aln1))

[1] 36 35 35 36 35 35

> head(ngap(aln1))

[1] 0 0 0 0 0 0
```

5.3 More accessor methods
