# Supplementary material for: Software for Computing and Annotating Genomic Ranges
Source: PLoS Comput Biol. 2013 Aug 8;9(8):e1003118. doi: 10.1371/journal.pcbi.1003118 (PMC3738458; doi:10.1371/journal.pcbi.1003118)
Supplement: Software S2 — The GenomicRanges package. The GenomicRanges package defines general purpose containers for storing genomic ranges as well as more specialized containers for storing alignments against a reference genome. (GZ) [file pcbi.1003118.s002.gz › GenomicRanges/inst/doc/GenomicRangesUseCases.pdf]

GenomicRanges Use Cases

Marc Carlson   Patrick Aboyoun

April 16, 2013

1 Introduction

This vignette focuses on common use cases involving the *GenomicRanges* package.

```
> library(GenomicRanges)
```

2 Chip-seq analysis

This section presents an example workflow using a subset of the ChIP-seq data for origin recognition complex (ORC) binding sites in *Saccharomyces cerevisiae* from the paper *Conserved nucleosome positioning defines replication origins*, Eaton et. al. (PMID 20351051). The subset consists of all the MAQ alignments to chromosome XIV for two replicates of ORC ChIP-seq data and is contained in the Bioconductor package *EatonEtAlChIPseq*. For illustration purposes we will focus on the first replicate.

```
> library(EatonEtAlChIPseq)
> data(orcAlignsRep1)
> orcAlignsRep1

class: AlignedRead
length: 478774 reads; width: 39 cycles
chromosome: chrXIV chrXIV ... chrXIV chrXIV
position: 2 4 ... 784295 784295
strand: + - ... + +
alignQuality: IntegerQuality
alignData varLabels: nMismatchBestHit mismatchQuality nExactMatch24 nOneMismatch24
```

2.1 Filtering alignments

In this subsection we will demonstrate how to perform three filtering operations on alignments produced by the MAQ software through the following restrictions:

- Number of mismatches in alignment must be ≤ 3 (guideline specified in paper)
- No duplicates of {chromosome, strand, position} combinations (PCR bias correction)
- An alignment on one strand must have a plausible alignment on the complementary strand ("pseudo" paired-read restriction)

The first two restrictions can be implemented using functionality from the *ShortRead* package, while the last one can be performed using operations within the *GenomicRanges* package.

In the previous section we loaded the `orcAlignsRep1` object, an instance of the *ShortRead* class *AlignedRead*. This object contains information on the characteristics of the read as well as its alignment to a reference genome, including information on the number of mismatches for the best alignment. Using functionality from the *ShortRead* package we can perform the first two filtering operations, which result in a subset that is roughly 18% of the size of the original MAQ alignment file.

```
> subsetRep1 <-
+   orcAlignsRep1[alignData(orcAlignsRep1)[["nMismatchBestHit"]] <= 3L]
> length(subsetRep1) / length(orcAlignsRep1)

[1] 0.96655

> subsetRep1 <- subsetRep1[occurrenceFilter(withSread=FALSE)(subsetRep1)]
> length(subsetRep1) / length(orcAlignsRep1)

[1] 0.180722

> subsetRep1

class: AlignedRead
length: 86525 reads; width: 39 cycles
chromosome: chrXIV chrXIV ... chrXIV chrXIV
position: 2 5 ... 784294 784295
strand: + + ... - +
alignQuality: IntegerQuality
alignData varLabels: nMismatchBestHit mismatchQuality nExactMatch24 nOneMismatch24
```

The last filtering criterion, a "pseudo" paired-read filter, requires an understanding of the interval spans for the alignments rather than just the "leftmost" alignment location, i.e. start location on the positive strand or end location on the negative strand, represented in the *AlignedRead* class. As such we will coerce the alignment subset contained in `subsetRep1` to a *GRanges* object using a `coerce` method from the *ShortRead* package.

```
> rangesRep1 <- as(subsetRep1, "GRanges")
> head(rangesRep1, 3)

GRanges with 3 ranges and 5 metadata columns:
      seqnames    ranges strand |                     id
         <Rle> <IRanges>  <Rle> |            <BStringSet>
  [1]   chrXIV   [2, 40]      + | X8193_200:5:175:690:668
  [2]   chrXIV   [5, 43]      + | X8193_200:5:206:446:786
  [3]   chrXIV   [6, 44]      + |  X8193_200:5:12:950:859
      nMismatchBestHit mismatchQuality nExactMatch24 nOneMismatch24
             <integer>       <integer>     <integer>      <integer>
  [1]                0               0             5              0
  [2]                0               0             5              0
  [3]                1               4             6              0
  ---
  seqlengths:
   chrXIV
       NA
```

AlignedRead objects lack information on chromosome length, so we will add it to the new `rangesRep1` object.

```
> seqlengths(rangesRep1) <- 784333
```

For our "pseudo" paired-read filter, we will use the authors' estimate of the mean fragment length (around 150 base pairs). In particular, we will construct a filter where each alignment on the plus strand must have a corresponding alignment somewhere within [100, 200] bp downstream on the minus strand and vice versa with those alignments on the minus strand.

This filtering process can be achieved through an interval overlap operation between the starts of the alignments on the minus strand

```
> negRangesRep1 <- rangesRep1[strand(rangesRep1) == "-"]
> negStartsRep1 <- resize(negRangesRep1, 1)
```

and the projected end of the alignments on the plus strand. Note: As of GenomicRanges >= 1.11.7, shift() no longer trims out of bound ranges. Because of this, we have a little more work to do for the positive strand ends.

```
> posRangesRep1 <- rangesRep1[strand(rangesRep1) == "+"]
> posEndsRep1 <- shift(posRangesRep1, 99)
> posEndsRep1 <- trim(posRangesRep1)
> posEndsRep1 <- resize(posEndsRep1, 100)
> strand(posEndsRep1) <- "-"
```

The results of the interval overlap are shown below. This filter flagged roughly 4.5% of the remaining alignments for removal, resulting in keeping 17.3% of the original set of alignments when including the results of the first two filtering steps.

```
> strandMatching <- findOverlaps(negStartsRep1, posEndsRep1)
> posKeep <- unique(subjectHits(strandMatching))
> negKeep <- unique(queryHits(strandMatching))
> length(posKeep) / length(posEndsRep1)

[1] 0.9607007

> length(negKeep) / length(negStartsRep1)

[1] 0.9586694

> (length(posKeep) + length(negKeep)) / length(orcAlignsRep1)

[1] 0.1734367

> posFilteredRangesRep1 <- posRangesRep1[posKeep]
> negFilteredRangesRep1 <- negRangesRep1[negKeep]
```

2.2 Finding peaks

Once the alignments have been filtered, they can be aggregated into coverage vectors. Assuming the size of the reads are uniformly distributed within [100, 200] base pairs, we will generate a coverage vector by giving full weight to alignments within 100 bp upstream and then linearly decreasing weights for the next 100 bp upstream. For the `runwtsum` function from *IRanges*, these weights for the positive and negative strands are expressed by the vectors:

```
> posWeights <- c(seq(0.01, 1, length = 100), rep(c(1, 0), c(101, 200)))
> negWeights <- rev(posWeights)
> plot(-200:200, posWeights, xlab = "Relative Position",
+      ylab = "Coverage Weight", type = "l")
```

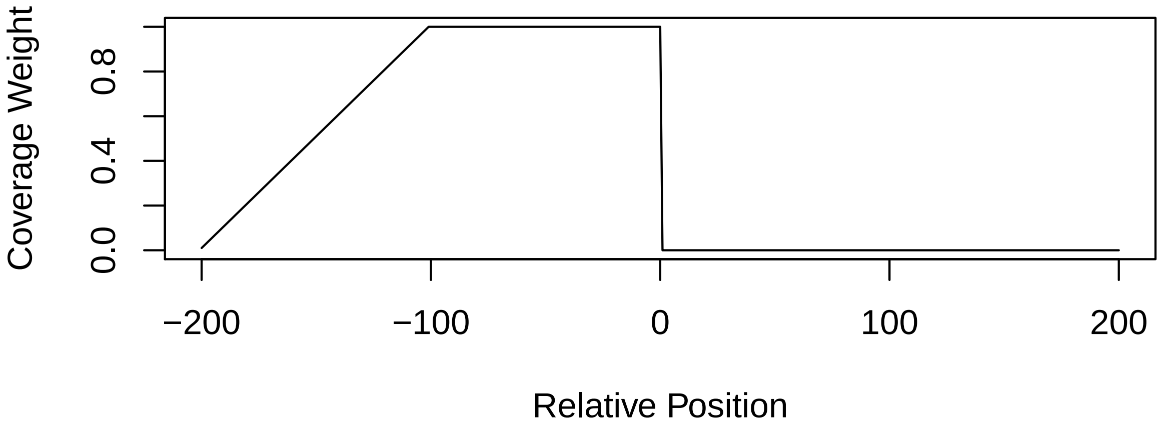

Figure 1: Coverage weights for positive strand weighted sums.

The first step in constructing this coverage vector is to tabulate the alignments by their start positions on both the positive and negative strand. We will use the `coverage` function on these start values, which will produce *RleList* representations of the coverage vectors.

```
> posStartsCoverRep1 <- coverage(resize(posFilteredRangesRep1, 1))
> negStartsCoverRep1 <- coverage(resize(negFilteredRangesRep1, 1))
```

The second step in the process is aggregating upstream alignments using the `posWeights` and `negWeights` objects defined above.

```
> posExtCoverRep1 <-
+   round(runwtsum(posStartsCoverRep1, k = 401, wt = posWeights,
+                  endrule = "constant"))
> negExtCoverRep1 <-
+   round(runwtsum(negStartsCoverRep1, k = 401, wt = negWeights,
+                  endrule = "constant"))
```

Before we proceeded any further, we will define two plot functions for visualizing coverage vectors: `plotCoverage` for displaying a single coverage vector and `plotStrandedCoverage` for displaying back-to-back coverage vectors for dual-stranded data.

```
> plotCoverage <-
+ function(x, xlab = "Position", ylab = "Coverage",...)
+ {
+     plot(c(start(x), length(x)), c(runValue(x), tail(runValue(x), 1)),
+          type = "s", col = "blue", xlab = xlab, ylab = ylab, ...)
+ }
> plotStrandedCoverage <-
```

```
+ function(positive, negative, xlab = "Position", ylab = "Coverage",...)
+ {
+     ylim <- min(max(positive), max(negative)) * c(-1, 1)
+     plotCoverage(positive, ylim = ylim, ...)
+     lines(c(start(negative), length(negative)),
+           - c(runValue(negative), tail(runValue(negative), 1)),
+           type = "s", col = "red")
+     abline(h = 0, col = "dimgray")
+ }
```

The coverage across chromosome XIV of the filtered alignements is shown in Figure 2. In general this plot shows a near mirror image of coverage vectors between the positive and negative strand.

```
> plotStrandedCoverage(posExtCoverRep1[[1]], negExtCoverRep1[[1]])
```

To reduce these measures to a single dimension, we will be conservative and choose the smallest value between the positive and negative strand coverage vectors using the `pmin` method for *RleList* objects.

```
> combExtCoverRep1 <- pmin(posExtCoverRep1, negExtCoverRep1)
> quantile(combExtCoverRep1, c(0.5, 0.9, 0.95))

    chrXIV
50%      4
90%     10
95%     14
```

We now can call peaks off the combined coverage object `combExtCoverRep1`. Since the median height for the combined coverage on chromosome XIV is 4, we can limit our attention to areas on the chromosome with coverage ≥ 5 using the `slice` function. From there we can derive a heuristic for a significant peaks as those achieving a maximum height ≥ 28, which selects 22 peaks.

```
> peaksRep1 <- slice(combExtCoverRep1, lower = 5)
> peakMaxsRep1 <- viewMaxs(peaksRep1)
> tail(sort(peakMaxsRep1[[1]]), 30)

 [1]  22  22  22  23  23  23  24  25  28  29  29  32  41  51  51  58  61
[18]  64  66  69  73  73  74  75  83  89  90 114 116 137

> peaksRep1 <- peaksRep1[peakMaxsRep1 >= 28]
> peakRangesRep1 <-
+   GRanges("chrXIV", as(peaksRep1[[1]], "IRanges"),
+           seqlengths = seqlengths(rangesRep1))
> length(peakRangesRep1)

[1] 22
```

We can now compare the significant peaks we selected with those selected by the authors. Using interval comparison tools, we see there is general agreement between our peaks and those of the authors.

```
> data(orcPeaksRep1)
> countOverlaps(orcPeaksRep1, peakRangesRep1)

 [1] 1 1 1 1 1 1 1 1 1 1 1 1 1 1 1 1 1 1 1 1

> countOverlaps(peakRangesRep1, orcPeaksRep1)

 [1] 0 2 0 1 1 1 1 1 1 1 1 1 2 1 1 0 0 1 1 1 1 1
```

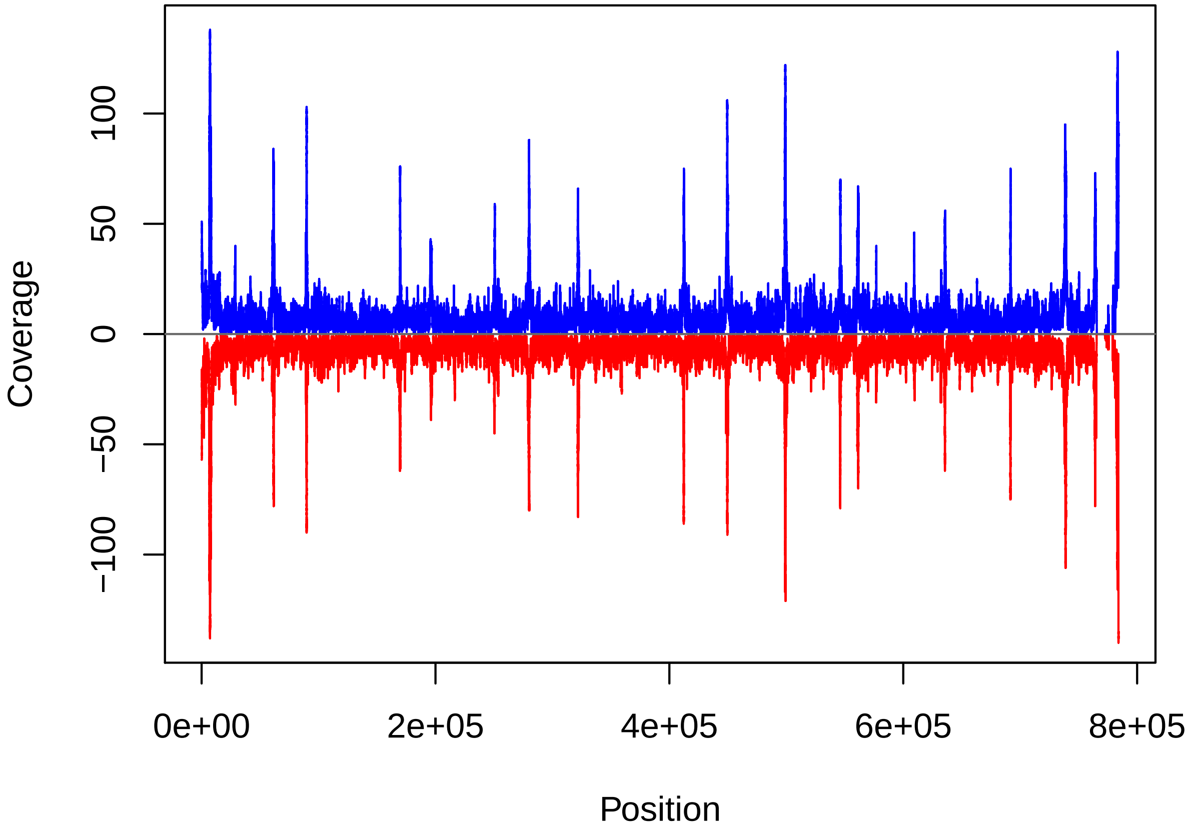

Figure 2: Plot of coverage across chromosome XIV.

3 Simple RNA-seq Analysis

For the first example we will focus on RNA-seq analysis, but these tools can also be applied to other kinds of short read data analysis. It is also necessary to consider limitations imposed by BAM files and the way that they are read in. BAM files can contain things that align to multiple places (multi-reads) and this makes things more complex since we don't know which things were aligned in this way. Therefore, if these things are not filtered out beforehand by the aligner, we will be counting some reads multiple times. Also, because alignments consider the per base qualities of a sequence, it is also possible for the same called sequence to be aligned onto two different places. When we scan in the BAM file, these sequences will be read in based on their predicted positions from the aligner. Thus, they can count as different sequences even though they appear to be the same sequence (based on their base pairs).

Let's begin by using the *Rsamtools* to retrieve some data from a yeast experiment and load it into a *GappedAlignments* object. Here we will load in a single file that contains RNA-seq data aligned from a wild type yeast experiment. This particular file only has data from chromosome 13. While more data can be

loaded, we will limit our analysis to this subset for expediency purposes.

```
> library(leeBamViews)
> testFile <- system.file("bam", "isowt5_13e.bam", package="leeBamViews")
> library(Rsamtools)
> aligns <- readBamGappedAlignments(testFile)
```

At this point, we also have to consider carefully which build we have so that we can know which annotation we need. This data is from Lee et al (PMID 19096707), which is available in the *leeBamViews* data package. This data originates from Gavin Sherlock's group at Stanford and so naturally they designed their experiments with the SGD genomes in mind. Therefore, it is probably reasonable to use the SGD genome assembly data for *Saccharomyces cerevisiae* when looking at their data. This data is available from UCSC and is therefore also accessible through the *rtracklayer*, *BSGenome* and *GenomicFeatures* packages.

3.1 Getting relevant genomic annotations

Now that we have chosen a data source to match the data that the genes were aligned to, we can simply get relevant annotation data to go with it. The following code will load the *GenomicFeatures* package, and then create an annotation database based on material from the sgdGene track.

```
> library(GenomicFeatures)
> txdb <- makeTranscriptDbFromUCSC(genome="sacCer2", tablename="sgdGene")
```

Then we simply have to retrieve data from the database using the `exonsBy` method so that only exonic locations for each transcript will be retrieved by setting the `by` argument to `"tx"`.

```
> exonRanges <- exonsBy(txdb, "tx")
> length(exonRanges)

[1] 6717

> exonRanges[1]

GRangesList of length 1:
$1
GRanges with 1 range and 3 metadata columns:
      seqnames     ranges strand |   exon_id   exon_name exon_rank
         <Rle>  <IRanges>  <Rle> | <integer> <character> <integer>
  [1]     chrI [335, 649]      + |         1        <NA>         1

---
seqlengths:
    chrI   chrII  chrIII    chrIV ...   chrXV  chrXVI    chrM 2micron
  230208  813178  316617  1531919 ... 1091289  948062   85779    6318
```

At this point, in spite of using the matching annotations for the data, there is still a discrepancy in how the chromosomes were named from one source to the next. In our data these all start with "Sc", whereas in the annotations they do not. Also, numbers of the chromosomes for the annotation are given in classic Roman numerals (as is traditional for yeast). So let's recode the alignments to conform with the exon annotations.

```
> seqlevels(exonRanges)
```

```
 [1] "chrI"    "chrII"   "chrIII"  "chrIV"   "chrV"    "chrVI"
 [7] "chrVII"  "chrVIII" "chrIX"   "chrX"    "chrXI"   "chrXII"
[13] "chrXIII" "chrXIV"  "chrXV"   "chrXVI"  "chrM"    "2micron"

> newlvls <- c(paste("chr", as.roman(1:16), sep=""), "chrM", "2micron")
> newlvls

 [1] "chrI"    "chrII"   "chrIII"  "chrIV"   "chrV"    "chrVI"
 [7] "chrVII"  "chrVIII" "chrIX"   "chrX"    "chrXI"   "chrXII"
[13] "chrXIII" "chrXIV"  "chrXV"   "chrXVI"  "chrM"    "2micron"

> seqlevels(exonRanges) <- newlvls  # reorder the levels
> names(newlvls) <- seqlevels(aligns)
> newlvls

  Scchr01   Scchr02   Scchr03   Scchr04   Scchr05   Scchr06   Scchr07
   "chrI"   "chrII"  "chrIII"   "chrIV"    "chrV"   "chrVI"  "chrVII"
  Scchr08   Scchr09   Scchr10   Scchr11   Scchr12   Scchr13   Scchr14
"chrVIII"   "chrIX"    "chrX"   "chrXI"  "chrXII" "chrXIII"  "chrXIV"
  Scchr15   Scchr16    Scmito      <NA>
  "chrXV"  "chrXVI"    "chrM" "2micron"

> seqlevels(aligns) <- newlvls  # rename the levels
> seqlengths(aligns) <- seqlengths(exonRanges)
```

3.2 Counting reads that overlap the annotations

Now we can count how many reads mapped to each transcript. And, since the `exonRanges` object only has ranges for exons, this use of `countOverlaps` will only consider the exon space when calculating the counts for each.

```
> counts <- countOverlaps(exonRanges, aligns)
```

For simplicities sake, we will now calculate the RPKM. This is not meant as an endorsement of any one measure over any other, it's just being used here as an example that users will probably have encountered before. Using the annotation object, we can also get the number of bases representing each `exonRanges` element. Note that this sum also only includes bases that are represented by the exons from each transcript.

```
> numBases <- sum(width(exonRanges))
> geneLengthsInKB <- numBases / 1000
```

And from this we could (as an example) calculate the sum of the mappable reads and then calculate the RPKMs. To do that we first would need to calculate the total number of reads being considered by the experiment in millions of reads.

```
> millionsMapped <- sum(counts) / 1000000
```

Then we have to calculate the RPM by considering the number of reads for each transcript relative to this value.

```
> # counted reads / total reads in millions
> rpm <- counts / millionsMapped
```

Finally, we calculate the RPKM by adjusting each RPM measurement for the length of the transcript in Kb.

```
> # reads per million per geneLength in Kb
> rpkm <- rpm / geneLengthsInKB
```

Once we have calculated a measure of gene expression, we might find it convenient to put it into a table as is expected by the *DESeq* package. To do this, we have to consider that we will probably want to do just the above steps that collect the raw counts and then place those vectors into a `data.frame` or `matrix` as a column. For the sake of saving space, lets assume you did that and created another vector called `counts2`, then you could create your *DESeq* object like this:

```
> deframe <- data.frame(counts, counts2)
```

This will work in this case for as many other samples as you choose to process because the countOvelaps method is always using the same `exonRanges` object to quantify overlap regions with.

3.3 Identifying and learning more about interesting transcripts

Now that we have calculated the RPKM, we can start to ask basic questions about the results. For example, which of the transcripts had the two highest scores? We can get this easily, because rpkm and exonRanges will both be listed in the same order.

```
> sortedRPKM <- sort(rpkm)
> highScoreGenes <- tail(sortedRPKM)
```

From the above output, notice how the names of the sortedRPKM are the same as the index of exonRanges we need to retrieve? We can therefore see that transcript 6678 and 6676 are also the IDs of the transcript with the highest RPKM. So we can use the `transcripts` accessor learn which gene that transcript is associated with like this:

```
> txs <- transcripts(txdb,
+                    vals=list(tx_id=names(highScoreGenes)),
+                    columns=c("tx_id","gene_id"))
> systNames <- as.vector(unlist(mcols(txs)[["gene_id"]]))
```

Once you know the gene id, you can use gene-centric annotation data sources such as the *org.Sc.sgd.db* package to learn more about it:

```
> library(org.Sc.sgd.db)
> toTable(org.Sc.sgdGENENAME[systNames])

  systematic_name gene_name
1         YMR297W      PRC1
2         YMR305C     SCW10
3         YMR307W      GAS1
```

If you wish, you can also use these systematic names to replace the rownames in any *DESeq* frame objects that you create.

3.4 Identifying reads that do NOT overlap known annotation

At this point you might be satisfied with calculating the amount of known genes in your model, but what if you wanted to know about regions that were transcribed but which were NOT found amongst the annotated transcripts?

Isolate regions not present in the annotation.

```
> noOlap <- aligns[!overlapsAny(aligns, exonRanges)]
```

Perform a `findOverlaps` to confirm no ranges overlap.

```
> findOverlaps(noOlap, exonRanges)

Hits of length 0
queryLength: 17238
subjectLength: 6717
```

It is interesting to note that in spite of having removed all known exons, this object still contains about half of the original set of aligned reads.

```
> length(noOlap)

[1] 17238
```

Create a subset of records that do overlap with the annotation using the `subsetByOverlaps` function.

```
> filtData <- subsetByOverlaps(aligns, exonRanges)
> length(filtData)

[1] 17311
```

If a coarser level of filtering is desired, you could also use the output of `transcriptsBy` as the subject argument for `subsetByOverlaps`. Doing so would look like this:

```
> filtData2 <- subsetByOverlaps(aligns, transcriptsBy(txdb, "gene"))
> length(filtData2)

[1] 17312
```

In this specific case, you can see that not many additional things have been filtered out by the coarser filtering which indicates that not many of these unknown aligned reads are from intronic or untranslated regions. This makes sense, since introns in yeast tend to be rare, infrequent and small. Going forward in this example, we will use the set that has only filtered out the exons just so that we can look at the set of reads that is complementary to the ones we just counted.

The next step in looking for transcripts that are unknown is to see where the reads are located on the genome. To do that we will use a function originally developed for chip-seq analysis. The `coverage` method.

```
> cov <- coverage(filtData)
```

Since we only have data for chromosome 13, lets subset down to that. Notice that in using the coverage method we lose the strand information. This is ok in this case, since the experiment has not preserved the strand information anyhow. If you had data that measured each strand separately, then you would want to call coverage twice at this point to keep the results separated.

```
> cov <- cov[13]
```

And now we can slice that data so that we only consider islands where things are continuously at least one read deep.

```
> islands <- slice(cov, lower = 1)
```

Having identified those, we can then filter out just the islands where the final width is at least 1000 bases long.

```
> transcribedRegions <- islands[width(islands) > 1000]
> txr <- islands[width(islands) > 1000]
```

Finally, we should then be able to go back to the `BSGenome` object and retrieve the actual sequences. In order to recover these sequences, we are going to specify that this data came from chromosome 13, and we will just retrieve the plus strand version of each (since the experiment collapsed that data anyhow).

```
> library(BSgenome.Scerevisiae.UCSC.sacCer2)
> getYeastSequence <- function(data) {
+   chr <- rep("chrXIII",length(start(data[[1]])))
+   starts <- start(data[[1]])
+   ends <- end(data[[1]])
+   strands <- rep("+",length(start(data[[1]])))
+   getSeq(Scerevisiae, names=chr, start=starts, end=ends, strand=strands)
+ }
> DNASet <- DNAStringSet(getYeastSequence(transcribedRegions))
```

4 Session Information

The version number of R and packages loaded for generating the vignette were:

```
R version 3.0.0 (2013-04-03)
Platform: x86_64-unknown-linux-gnu (64-bit)

locale:
 [1] LC_CTYPE=en_US.UTF-8       LC_NUMERIC=C
 [3] LC_TIME=en_US.UTF-8        LC_COLLATE=C
 [5] LC_MONETARY=en_US.UTF-8    LC_MESSAGES=en_US.UTF-8
 [7] LC_PAPER=C                 LC_NAME=C
 [9] LC_ADDRESS=C               LC_TELEPHONE=C
[11] LC_MEASUREMENT=en_US.UTF-8 LC_IDENTIFICATION=C

attached base packages:
[1] parallel  stats     graphics  grDevices utils     datasets
[7] methods   base

other attached packages:
 [1] BSgenome.Scerevisiae.UCSC.sacCer2_1.3.19
 [2] org.Sc.sgd.db_2.9.0
 [3] RSQLite_0.11.3
 [4] DBI_0.2-5
 [5] GenomicFeatures_1.12.0
 [6] AnnotationDbi_1.22.1
 [7] leeBamViews_0.99.22
 [8] BSgenome_1.28.0
 [9] Biobase_2.20.0
[10] EatonEtAlChIPseq_0.0.9
[11] rtracklayer_1.20.1
[12] ShortRead_1.18.0
[13] latticeExtra_0.6-24
[14] RColorBrewer_1.0-5
```

```
[15] lattice_0.20-15
[16] Rsamtools_1.12.1
[17] Biostrings_2.28.0
[18] GenomicRanges_1.12.2
[19] IRanges_1.18.0
[20] BiocGenerics_0.6.0

loaded via a namespace (and not attached):
[1] RCurl_1.95-4.1 XML_3.96-1.1   biomaRt_2.16.0 bitops_1.0-5
[5] grid_3.0.0     hwriter_1.3    stats4_3.0.0   tools_3.0.0
[9] zlibbioc_1.6.0
```
