# Supplementary material for: Software for Computing and Annotating Genomic Ranges
Source: PLoS Comput Biol. 2013 Aug 8;9(8):e1003118. doi: 10.1371/journal.pcbi.1003118 (PMC3738458; doi:10.1371/journal.pcbi.1003118)
Supplement: Software S2 — The GenomicRanges package. The GenomicRanges package defines general purpose containers for storing genomic ranges as well as more specialized containers for storing alignments against a reference genome. (GZ) [file pcbi.1003118.s002.gz › GenomicRanges/inst/doc/OverlapEncodings.pdf]

Overlap encodings

Hervé Pagès

Last modified: August 2012; Compiled: April 16, 2013

Contents

1 Introduction **2**

2 Load reads from a BAM file **2**
2.1 Load single-end reads from a BAM file 2
2.2 Load paired-end reads from a BAM file 3

3 Validate the alignments produced by the aligner **5**
3.1 Validate the single-end alignments 6
3.1.1 Load the *original query sequences* 6
3.1.2 Compute the *reference query sequences* 6
3.1.3 Compare the *original query sequences* with the *reference query sequences* 7
3.2 Validate the paired-end alignments 8
3.2.1 Load the *original query sequences* 8
3.2.2 Compute the *reference query sequences* 9
3.2.3 Compare the *original query sequences* with the *reference query sequences* 9
3.3 Conclusion 10

4 Find all the overlaps between the reads and transcripts **10**
4.1 Load the transcripts from a *TranscriptDb* object 10
4.2 Single-end overlaps 12
4.2.1 Find the single-end overlaps 12
4.2.2 Tabulate the single-end overlaps 12
4.3 Paired-end overlaps 13
4.3.1 Find the paired-end overlaps 13
4.3.2 Tabulate the paired-end overlaps 14

5 Encode the overlaps between the reads and transcripts **15**
5.1 Single-end encodings 15
5.2 Paired-end encodings 16

6 "Compatible" overlaps **17**
6.1 "Compatible" single-end overlaps 17
6.1.1 "Compatible" single-end encodings 17
6.1.2 Tabulate the "compatible" single-end overlaps 18
6.2 "Compatible" paired-end overlaps 19
6.2.1 "Compatible" paired-end encodings 19
6.2.2 Tabulate the "compatible" paired-end overlaps 21

7 Project the alignments on the transcriptome **22**
7.1 Project the single-end alignments on the transcriptome 22
7.2 Project the paired-end alignments on the transcriptome 23

8 Align the reads to the transcriptome **24**
8.1 Align the single-end reads to the transcriptome 24
8.1.1 Find the "hits" 24
8.1.2 Tabulate the "hits" 26
8.1.3 A closer look at the "hits" 26
8.2 Align the paired-end reads to the transcriptome 27

9 "Almost compatible" overlaps **27**
9.1 "Almost compatible" single-end overlaps 27
9.1.1 "Almost compatible" single-end encodings 27
9.1.2 Tabulate the "almost compatible" single-end overlaps 28
9.2 "Almost compatible" paired-end overlaps 29
9.2.1 "Almost compatible" paired-end encodings 29
9.2.2 Tabulate the "almost compatible" paired-end overlaps 30

10 Detect novel splice junctions **31**
10.1 By looking at single-end overlaps 31
10.2 By looking at paired-end overlaps 32

11 `sessionInfo()` **32**

1 Introduction

In the context of an RNA-seq experiment, encoding the overlaps between the aligned reads and the transcripts can be used for detecting those overlaps that are "compatible" with the splicing of the transcript.

Various tools are provided in the IRanges and GenomicRanges packages for working with *overlap encodings*. In this vignette, we illustrate the use of these tools on the single-end and paired-end reads of an RNA-seq experiment.

2 Load reads from a BAM file

2.1 Load single-end reads from a BAM file

BAM file `untreated1_chr4.bam` (located in the pasillaBamSubset data package) contains single-end reads from the "Pasilla" experiment and aligned against the dm3 genome (see `?untreated1_chr4` in the pasillaBamSubset package for more information about those reads):

```
> library(pasillaBamSubset)
> untreated1_chr4()
```

```
[1] "/home/biocbuild/bbs-2.12-bioc/R/library/pasillaBamSubset/extdata/untreated1_chr4.bam"
```

We use the `readGappedAlignments` function defined in the GenomicRanges package to load the reads into a *GappedAlignments* object. It's probably a good idea to get rid of the PCR or optical duplicates (flag bit 0x400 in the SAM format, see the SAM Spec [1] for the details), as well as reads not passing quality controls (flag bit 0x200 in the SAM format). We do this by creating a *ScanBamParam* object that we pass to `readGappedAlignments` (see `?ScanBamParam` in the Rsamtools package for the details). Note that we also use `use.names=TRUE` in order to load the *query names* (aka *query template names*, see QNAME field in the SAM Spec) from the BAM file (`readGappedAlignments` will use them to set the names of the returned object):

```
> library(GenomicRanges)
> library(Rsamtools)
> flag0 <- scanBamFlag(isDuplicate=FALSE, isNotPassingQualityControls=FALSE)
> param0 <- ScanBamParam(flag=flag0)
> U1.GAL <- readGappedAlignments(untreated1_chr4(), use.names=TRUE, param=param0)
> head(U1.GAL)
```

[1] `http://samtools.sourceforge.net/`

```
GappedAlignments with 6 alignments and 0 metadata columns:
                    seqnames strand       cigar    qwidth     start       end     width      ngap
                       <Rle>  <Rle> <character> <integer> <integer> <integer> <integer> <integer>
  SRR031729.3941844     chr4      -         75M        75       892       966        75         0
  SRR031728.3674563     chr4      -         75M        75       919       993        75         0
  SRR031729.8532600     chr4      +         75M        75       924       998        75         0
  SRR031729.2779333     chr4      +         75M        75       936      1010        75         0
  SRR031728.2826481     chr4      +         75M        75       949      1023        75         0
  SRR031728.2919098     chr4      -         75M        75       967      1041        75         0
  ---
  seqlengths:
      chr2L    chr2R    chr3L    chr3R     chr4      chrM     chrX  chrYHet
   23011544 21146708 24543557 27905053  1351857     19517 22422827   347038
```

Because the aligner used to align those reads can report more than 1 alignment per *original query* (i.e. per read stored in the input file, typically a FASTQ file), we shouldn't expect the names of `U1.GAL` to be unique:

```
> U1.GAL_names_is_dup <- duplicated(names(U1.GAL))
> table(U1.GAL_names_is_dup)
```

```
U1.GAL_names_is_dup
 FALSE   TRUE
190770  13585
```

Storing the *query names* in a factor will be useful as we will see later in this document:

```
> U1.uqnames <- unique(names(U1.GAL))
> U1.GAL_qnames <- factor(names(U1.GAL), levels=U1.uqnames)
```

Note that we explicitely provide the levels of the factor to enforce their order. Otherwise `factor()` would put them in lexicographic order which is not advisable because it depends on the locale in use.

Another object that will be useful to keep near at hand is the mapping between each *query name* and its first occurence in `U1.GAL_qnames`:

```
> U1.GAL_dup2unq <- match(U1.GAL_qnames, U1.GAL_qnames)
```

Our reads can have up to 2 gaps (a gap corresponds to an N operation in the CIGAR):

```
> head(unique(cigar(U1.GAL)))
```

```
[1] "75M"          "35M6727N40M"  "22M6727N53M"  "13M6727N62M"  "26M292N49M"   "62M21227N13M"
```

```
> table(ngap(U1.GAL))
```

```
     0      1      2
184039  20169    147
```

Also, the following table indicates that indels were not allowed/supported during the alignment process (no I or D CIGAR operations):

```
> colSums(cigarOpTable(cigar(U1.GAL)))
```

```
       M        I        D        N        S        H        P
15326625        0        0 21682582        0        0        0
```

2.2 Load paired-end reads from a BAM file

BAM file `untreated3_chr4.bam` (located in the pasillaBamSubset data package) contains paired-end reads from the "Pasilla" experiment and aligned against the dm3 genome (see `?untreated3_chr4` in the pasillaBamSubset package for more information about those reads). We use the `readGappedAlignmentPairs` function to load them into a *GappedAlignmentPairs* object:

```
> U3.galp <- readGappedAlignmentPairs(untreated3_chr4(), use.names=TRUE, param=param0)
> head(U3.galp)
```

```
GappedAlignmentPairs with 6 alignment pairs and 0 metadata columns:
                  seqnames strand :      ranges --       ranges
                     <Rle>  <Rle> :   <IRanges> --    <IRanges>
SRR031715.1138209     chr4      + : [169,  205] -- [ 326,  362]
 SRR031714.756385     chr4      + : [943,  979] -- [1086, 1122]
SRR031714.2355189     chr4      + : [944,  980] -- [1119, 1155]
SRR031714.5054563     chr4      + : [946,  982] -- [ 986, 1022]
SRR031715.1722593     chr4      + : [966, 1002] -- [1108, 1144]
SRR031715.2202469     chr4      + : [966, 1002] -- [1114, 1150]
---
seqlengths:
    chr2L    chr2R    chr3L    chr3R     chr4     chrM     chrX  chrYHet
 23011544 21146708 24543557 27905053  1351857    19517 22422827   347038
```

The `show` method for *GappedAlignmentPairs* objects displays two `ranges` columns, one for the *first* alignment in the pair (the left column), and one for the *last* alignment in the pair (the right column). The `strand` column corresponds to the strand of the *first* alignment.

```
> head(first(U3.galp))
```

```
GappedAlignments with 6 alignments and 0 metadata columns:
                    seqnames strand       cigar    qwidth     start       end     width      ngap
                       <Rle>  <Rle> <character> <integer> <integer> <integer> <integer> <integer>
  SRR031715.1138209     chr4      +         37M        37       169       205        37         0
   SRR031714.756385     chr4      +         37M        37       943       979        37         0
  SRR031714.2355189     chr4      +         37M        37       944       980        37         0
  SRR031714.5054563     chr4      +         37M        37       946       982        37         0
  SRR031715.1722593     chr4      +         37M        37       966      1002        37         0
  SRR031715.2202469     chr4      +         37M        37       966      1002        37         0
  ---
  seqlengths:
      chr2L    chr2R    chr3L    chr3R     chr4     chrM     chrX  chrYHet
   23011544 21146708 24543557 27905053  1351857    19517 22422827   347038
```

```
> head(last(U3.galp))
```

```
GappedAlignments with 6 alignments and 0 metadata columns:
                    seqnames strand       cigar    qwidth     start       end     width      ngap
                       <Rle>  <Rle> <character> <integer> <integer> <integer> <integer> <integer>
  SRR031715.1138209     chr4      -         37M        37       326       362        37         0
   SRR031714.756385     chr4      -         37M        37      1086      1122        37         0
  SRR031714.2355189     chr4      -         37M        37      1119      1155        37         0
  SRR031714.5054563     chr4      -         37M        37       986      1022        37         0
  SRR031715.1722593     chr4      -         37M        37      1108      1144        37         0
  SRR031715.2202469     chr4      -         37M        37      1114      1150        37         0
  ---
  seqlengths:
      chr2L    chr2R    chr3L    chr3R     chr4     chrM     chrX  chrYHet
   23011544 21146708 24543557 27905053  1351857    19517 22422827   347038
```

According to the SAM format specifications, the aligner is expected to mark each alignment pair as *proper* or not (flag bit 0x2 in the SAM format). The SAM Spec only says that a pair is *proper* if the *first* and *last* alignments in the pair are "properly aligned according to the aligner". So the exact criteria used for setting this flag is left to the aligner.

We use `isProperPair` to extract this flag from the *GappedAlignmentPairs* object:

```
> table(isProperPair(U3.galp))
```

```
FALSE  TRUE
29518 45828
```

Even though we could do *overlap encodings* with the full object, we keep only the *proper* pairs for our downstream analysis:

```
> U3.GALP <- U3.galp[isProperPair(U3.galp)]
```

Because the aligner used to align those reads can report more than 1 alignment per *original query template* (i.e. per pair of sequences stored in the input files, typically 1 FASTQ file for the *first* ends and 1 FASTQ file for the *last* ends), we shouldn't expect the names of `U3.GALP` to be unique:

```
> U3.GALP_names_is_dup <- duplicated(names(U3.GALP))
> table(U3.GALP_names_is_dup)
```

```
U3.GALP_names_is_dup
FALSE  TRUE
43659  2169
```

Storing the *query template names* in a factor will be useful:

```
> U3.uqnames <- unique(names(U3.GALP))
> U3.GALP_qnames <- factor(names(U3.GALP), levels=U3.uqnames)
```

as well as having the mapping between each *query template name* and its first occurence in `U3.GALP_qnames`:

```
> U3.GALP_dup2unq <- match(U3.GALP_qnames, U3.GALP_qnames)
```

Our reads can have up to 1 gap per end:

```
> head(unique(cigar(first(U3.GALP))))
```

```
[1] "37M"        "6M58N31M"   "25M56N12M"  "19M62N18M"  "29M222N8M"  "9M222N28M"
```

```
> head(unique(cigar(last(U3.GALP))))
```

```
[1] "37M"          "12M58N25M"    "19M58N18M"    "27M2339N10M" "29M2339N8M"   "9M222N28M"
```

```
> table(ngap(first(U3.GALP)), ngap(last(U3.GALP)))
```

```
        0     1
  0 44510   596
  1   637    85
```

Like for our single-end reads, the following tables indicate that indels were not allowed/supported during the alignment process:

```
> colSums(cigarOpTable(cigar(first(U3.GALP))))
```

```
      M       I       D       N       S       H       P
1695636       0       0  673919       0       0       0
```

```
> colSums(cigarOpTable(cigar(last(U3.GALP))))
```

```
      M       I       D       N       S       H       P
1695636       0       0  630395       0       0       0
```

3 Validate the alignments produced by the aligner

In this section we show how to validate the alignments produced by the aligner by comparing the *original query sequences* (aka "true" or "real" query sequences, or query sequences **before** alignment) with the *reference query sequences* (i.e. the query sequences **after** alignment).

Note that even though this step is not strictly required for computing the *overlap encodings*, some of the concepts and string-based computations described in this section are slightly related to some ideas introduced later in this document.

3.1 Validate the single-end alignments

3.1.1 Load the *original query sequences*

To load the *original query sequences*, we reload the BAM file but now we explicitely request the SEQ field by using `what="seq"` in our call to `ScanBamParam`. To further validate the alignments produced by the aligner, we also need to load the NM tag which is a predefined tag described in the SAM Spec as the "Edit distance to the reference, including ambiguous bases but excluding clipping" (note that tags are optional fields i.e. not all BAM files have them):

```
> param1 <- ScanBamParam(flag=flag0, what="seq", tag="NM")
> U1.GAL <- readGappedAlignments(untreated1_chr4(), use.names=TRUE, param=param1)
> U1.GAL_qseq <- mcols(U1.GAL)$seq
> names(U1.GAL_qseq) <- names(U1.GAL)
> head(U1.GAL_qseq)

  A DNAStringSet instance of length 6
    width seq                                                  names
[1]    75 CTGTGGTGACCAACACCACAGAATGGTTCGGGC...GGGTTCCCTGCCCCTTTCCTGGCTAGGTTGTCC SRR031729.3941844
[2]    75 TCGGGCCCAATTAGAGGGTTCCCTGCCCCTTTC...TTGTCCGCTAGCTCATTTCCTGGGCTGTTGTTG SRR031728.3674563
[3]    75 CCCAATTAGAGGATTCTCTGCCCCTTTCCTGGC...CGGTAGCTCATTTCCCGGGATGTTGTTGTGTCC SRR031729.8532600
[4]    75 GTTCTCTGCCCCTTTCCTGGCTAGGTTGTCCGC...TCCCGAGATGTTGTTGTGTCCCGGGACCCACCT SRR031729.2779333
[5]    75 TTCCTGGCTAGGTTGTCCGCTAGCTCATTTCCC...TTGTGTCCCGGGACACACCTTATTGTGAGTTTG SRR031728.2826481
[6]    75 GCTAGCTCATTTCCCGGGAGGTTGTTGTGTCCC...CTTATTGTGAGTTTGTTGACAGCTCCAAGTTTG SRR031728.2919098
```

Because the BAM format imposes that the read sequence is "reverse complemented" when the read is aligned to the minus strand, we "reverse complement" it again to get the *original query sequences*:

```
> U1.GAL_oqseq <- U1.GAL_qseq
> U1.GAL_is_on_minus <- as.logical(strand(U1.GAL) == "-")
> U1.GAL_oqseq[U1.GAL_is_on_minus] <- reverseComplement(U1.GAL_oqseq[U1.GAL_is_on_minus])
> head(U1.GAL_oqseq)

  A DNAStringSet instance of length 6
    width seq                                                  names
[1]    75 GGACAACCTAGCCAGGAAAGGGGCAGGGAACCC...GCCCGAACCATTCTGTGGTGTTGGTCACCACAG SRR031729.3941844
[2]    75 CAACAACAGCCCAGGAAATGAGCTAGCGGACAA...GAAAGGGGCAGGGAACCCTCTAATTGGGCCCGA SRR031728.3674563
[3]    75 CCCAATTAGAGGATTCTCTGCCCCTTTCCTGGC...CGGTAGCTCATTTCCCGGGATGTTGTTGTGTCC SRR031729.8532600
[4]    75 GTTCTCTGCCCCTTTCCTGGCTAGGTTGTCCGC...TCCCGAGATGTTGTTGTGTCCCGGGACCCACCT SRR031729.2779333
[5]    75 TTCCTGGCTAGGTTGTCCGCTAGCTCATTTCCC...TTGTGTCCCGGGACACACCTTATTGTGAGTTTG SRR031728.2826481
[6]    75 CAAACTTGGAGCTGTCAACAAACTCACAATAAG...GGGACACAACAACCTCCCGGGAAATGAGCTAGC SRR031728.2919098
```

Note that sequences with the same *query name* correspond to the same *original query* and therefore must be the same. Let's do a quick sanity check:

```
> stopifnot(all(U1.GAL_oqseq == U1.GAL_oqseq[U1.GAL_dup2unq]))
```

Finally, let's reduce `U1.GAL_oqseq` to one *original query sequence* per unique *query name*:

```
> U1.oqseq <- U1.GAL_oqseq[!U1.GAL_names_is_dup]
```

If we had access to the FASTQ file used as input to the aligner, `U1.oqseq` would be the subset of this file made of the sequences with at least 1 alignment reported in BAM file `untreated1_chr4.bam`.

3.1.2 Compute the *reference query sequences*

The *reference query sequences* can easily be computed by extracting the nucleotides mapped to each read from the reference genome. This of course requires that we have access to the reference genome used by the aligner. In Bioconductor, the full genome sequence for the dm3 assembly is stored in the BSgenome.Dmelanogaster.UCSC.dm3 data package [2]:

[2] See `http://bioconductor.org/packages/release/data/annotation/` for the full list of annotation packages available in the current release of Bioconductor.

```
> library(BSgenome.Dmelanogaster.UCSC.dm3)
> Dmelanogaster

Fly genome
|
| organism: Drosophila melanogaster (Fly)
| provider: UCSC
| provider version: dm3
| release date: Apr. 2006
| release name: BDGP Release 5
|
| single sequences (see '?seqnames'):
|   chr2L      chr2R      chr3L      chr3R      chr4       chrX       chrU       chrM
|   chr2LHet   chr2RHet   chr3LHet   chr3RHet   chrXHet    chrYHet    chrUextra
|
| multiple sequences (see '?mseqnames'):
|   upstream1000  upstream2000  upstream5000
|
| (use the '$' or '[[' operator to access a given sequence)
```

Let's start by converting our *GappedAlignments* object `U1.GAL` into a *GRangesList* object:

```
> U1.grl <- grglist(U1.GAL, order.as.in.query=TRUE)
```

To extract the portions of the reference genome corresponding to the ranges in `U1.grl`, we can use the `extractTranscriptsFromGenome` function defined in the GenomicFeatures package:

```
> library(GenomicFeatures)
> U1.GAL_rqseq <- extractTranscriptsFromGenome(Dmelanogaster, U1.grl)
> head(U1.GAL_rqseq)

  A DNAStringSet instance of length 6
    width seq                                                    names
[1]    75 GGACAACCTAGCCAGGAAAGGGGCAGAGAACCC...GCCCGAACCATTCTGTGGTGTTGGTCACCACAG SRR031729.3941844
[2]    75 CAACAACATCCCGGGAAATGAGCTAGCGGACAA...GAAAGGGGCAGAGAACCCTCTAATTGGGCCCGA SRR031728.3674563
[3]    75 CCCAATTAGAGGGTTCTCTGCCCCTTTCCTGGC...CGCTAGCTCATTTCCCGGGATGTTGTTGTGTCC SRR031729.8532600
[4]    75 GTTCTCTGCCCCTTTCCTGGCTAGGTTGTCCGC...TCCCGGGATGTTGTTGTGTCCCGGGACCCACCT SRR031729.2779333
[5]    75 TTCCTGGCTAGGTTGTCCGCTAGCTCATTTCCC...TTGTGTCCCGGGACCCACCTTATTGTGAGTTTG SRR031728.2826481
[6]    75 CAAACTTGGAGCTGTCAACAAACTCACAATAAG...GGGACACAACAACATCCCGGGAAATGAGCTAGC SRR031728.2919098
```

3.1.3 Compare the *original query sequences* with the *reference query sequences*

We can use the `neditAt` function defined in the Biostrings package to compute the edit distance between 2 strings. Because the aligned reads have no indels, we should only see mismatches (typically a small number) during that comparison so we don't need to call `neditAt` with `with.indels=TRUE`. And because calling `neditAt` in a loop is inefficient, we only do this comparison for the first 500 sequences in `U1.GAL_oqseq`:

```
> U1.GAL_nedit500 <- sapply(1:500, function(i) neditAt(U1.GAL_oqseq[[i]], U1.GAL_rqseq[[i]]))
> table(U1.GAL_nedit500)

U1.GAL_nedit500
  0   1   2   3   4   5 
273 101  97  22   6   1 
```

Yes, the first 500 sequences in `U1.GAL_oqseq` are "close" to the first 500 sequences in `U1.GAL_rqseq`.

Now let's compare the edit distance reported by `neditAt` with the edit distance reported by the aligner (NM tag). Because the latter excludes the N CIGAR operations, it should actually be the same as the former. We confirm this for the 500 edit distances computed in `U1.GAL_nedit500`:

```
> U1.GAL_NM <- mcols(U1.GAL)$NM
> stopifnot(all(U1.GAL_NM[1:500] == U1.GAL_nedit500))
```

Note that the maximum observed number of mismatches tells us how many mismatches per read were tolerated by the aligner:

```
> table(U1.GAL_NM)

U1.GAL_NM
     0      1      2      3      4      5      6 
135911  44033  15684   5172   2233    948    374 
```

3.2 Validate the paired-end alignments

3.2.1 Load the *original query sequences*

To load the *original query sequences*, we reload the BAM file and request the SEQ field (and also the NM tag). Since we've removed the improper pairs from our current U3.GALP object, we need to do this again but now we do it at load time which is equivalent to doing it afterward (i.e. not only do we have the guarantee to end up with the same elements in U3.GALP, but also to have them in the same order):

```
> flag2 <- scanBamFlag(isDuplicate=FALSE,
+                      isNotPassingQualityControls=FALSE,
+                      isProperPair=TRUE)
> param2 <- ScanBamParam(flag=flag2, what="seq", tag="NM")
> U3.GALP <- readGappedAlignmentPairs(untreated3_chr4(), use.names=TRUE, param=param2)
```

Let's extract the *first* and *last* sequences from U3.GALP:

```
> U3.GALP_qseq1 <- mcols(first(U3.GALP))$seq
> U3.GALP_qseq2 <- mcols(last(U3.GALP))$seq
> names(U3.GALP_qseq1) <- names(U3.GALP_qseq2) <- names(U3.GALP)
> head(U3.GALP_qseq1)

  A DNAStringSet instance of length 6
    width seq                                               names               
[1]    37 CCGTTTCTGAAGGAGATGGCTCATGGAGTACCTGCCT             SRR031715.1138209
[2]    37 GCCCCTTTCCTGGCTAGGTTGTCCGCTAGCTCATTTC             SRR031714.756385
[3]    37 CCTTTCCTGGCTAGGTTGTCCGCTAGCTCATTTCCCG             SRR031714.5054563
[4]    37 CGCTAGCTCATTTCCCGGGCTGTTGTTGTGTCCCGGG             SRR031715.1722593
[5]    37 CGCTAGCTCATTTCCCGAGATGTTGTTGTGTCCCGGG             SRR031715.2202469
[6]    37 GCTTTGCTGAGCGCCTTTATGGCTGCTTGACTATCAG             SRR031714.3544437

> head(U3.GALP_qseq2)

  A DNAStringSet instance of length 6
    width seq                                               names               
[1]    37 GTCTCCAGCAGAGCAGATGGAGCAACGGCCTATAGAG             SRR031715.1138209
[2]    37 AGCTTTGCTGAGCGCCTTTATGGCTGCTTGACTATCA             SRR031714.756385
[3]    37 TGTTGTTGTGTCCCGGGACCCACCTTATTGTGAGTTT             SRR031714.5054563
[4]    37 GCTGCTTGACTATCAGACAGTATAGCAATGTCCTTGC             SRR031715.1722593
[5]    37 TGACTATCAGACAGTATAGCAATGTCCTTGCCATGAT             SRR031715.2202469
[6]    37 GTCCGCTAGCTCATTTCCCGGGATGTTTTTGTGTCCC             SRR031714.3544437
```

To obtain the *original query sequences* we "reverse complement" the sequences that are aligned to the minus strand:

```
> U3.GALP_oqseq1 <- U3.GALP_qseq1
> U3.GALP_first_is_on_minus <- as.logical(strand(first(U3.GALP)) == "-")
> U3.GALP_oqseq1[U3.GALP_first_is_on_minus] <- reverseComplement(U3.GALP_oqseq1[U3.GALP_first_is_on_minus])
> U3.GALP_oqseq2 <- U3.GALP_qseq2
> U3.GALP_last_is_on_minus <- as.logical(strand(last(U3.GALP)) == "-")
> U3.GALP_oqseq2[U3.GALP_last_is_on_minus] <- reverseComplement(U3.GALP_oqseq2[U3.GALP_last_is_on_minus])
```

Note that sequence pairs with the same *query template name* correspond to the same *original query pairs* and therefore should be the same. Let's do a quick sanity check:

```
> stopifnot(all(U3.GALP_oqseq1 == U3.GALP_oqseq1[U3.GALP_dup2unq]))
> stopifnot(all(U3.GALP_oqseq2 == U3.GALP_oqseq2[U3.GALP_dup2unq]))
```

Finally, let's reduce `U3.GALP_oqseq1` and `U3.GALP_oqseq2` to one *original query sequence* per unique *query template name*:

```
> U3.oqseq1 <- U3.GALP_oqseq1[!U3.GALP_names_is_dup]
> U3.oqseq2 <- U3.GALP_oqseq2[!U3.GALP_names_is_dup]
```

If we had access to the 2 FASTQ files used as input to the aligner, `U3.oqseq1` and `U3.oqseq2` would be the subsets of those files made of the sequence pairs with at least 1 alignment pair reported in BAM file `untreated3_chr4.bam`.

3.2.2 Compute the *reference query sequences*

Because our reads are paired-end, we extract separately the ranges corresponding to their *first* ends (aka *first* segments in BAM jargon) and those corresponding to their *last* ends (aka *last* segments in BAM jargon):

```
> U3.grl_first <- grglist(first(U3.GALP), order.as.in.query=TRUE)
> U3.grl_last <- grglist(last(U3.GALP, invert.strand=TRUE), order.as.in.query=TRUE)
```

Then we extract the portions of the reference genome corresponding to the ranges in *GRangesList* objects `U3.grl_first` and `U3.grl_last`:

```
> U3.GALP_rqseq1 <- extractTranscriptsFromGenome(Dmelanogaster, U3.grl_first)
> U3.GALP_rqseq2 <- extractTranscriptsFromGenome(Dmelanogaster, U3.grl_last)
```

3.2.3 Compare the *original query sequences* with the *reference query sequences*

Because the aligned reads have no indels, we should only see mismatches (typically a small number) during that comparison so we don't need to call `neditAt` with `with.indels=TRUE`. Let's do this comparison for the first 500 sequences in `U3.GALP_oqseq1` and in `reverseComplement(U3.GALP_oqseq2)`:

```
> U3.GALP_first_nedit500 <- sapply(1:500, function(i)
+     neditAt(U3.GALP_oqseq1[[i]], U3.GALP_rqseq1[[i]])
+ )
> table(U3.GALP_first_nedit500)

U3.GALP_first_nedit500
  0   1   2
337 135  28

> U3.GALP_last_nedit500 <- sapply(1:500, function(i)
+     neditAt(reverseComplement(U3.GALP_oqseq2[[i]]), U3.GALP_rqseq2[[i]])
+ )
> table(U3.GALP_last_nedit500)

U3.GALP_last_nedit500
  0   1   2
341 121  38
```

Yes, the first 500 sequences in `U3.GALP_oqseq1` and in `reverseComplement(U3.GALP_oqseq2)` are "close" to the first 500 sequences in `U3.GALP_rqseq1` and in `U3.GALP_rqseq2`, respectively.

Now let's compare the edit distance reported by `neditAt` with the edit distance reported by the aligner (NM tag). Because the latter excludes the N CIGAR operations, it should actually be the same as the former. We confirm this for the 500 edit distances computed in `U3.GALP_first_nedit500` and `U3.GALP_last_nedit500`:

```
> U3.GALP_first_NM <- mcols(first(U3.GALP))$NM
> stopifnot(all(U3.GALP_first_NM[1:500] == U3.GALP_first_nedit500))
> U3.GALP_last_NM <- mcols(last(U3.GALP))$NM
> stopifnot(all(U3.GALP_last_NM[1:500] == U3.GALP_last_nedit500))
```

Note that the following table tells us how many mismatches per read were tolerated by the aligner:

```
> table(U3.GALP_first_NM, U3.GALP_last_NM)

               U3.GALP_last_NM
U3.GALP_first_NM     0     1     2
               0 30140  4648  1292
               1  5023  1522   760
               2  1240   700   503
```

Up to 2 mismatches per end.

3.3 Conclusion

In addition to validate the alignments produced by the aligner, the validation described in this section is also an efficient and accurate way to make sure that the reference genome we've picked up is the same as the reference genome used by the aligner, at least for the regions covered by the reads.

In other words, if it's known that the 2 reference genomes are different, then this validation could still be performed, and, if successful, would indicate that the 2 genomes are probably substitutable for most analysis happening downstream of the BAM file.

4 Find all the overlaps between the reads and transcripts

4.1 Load the transcripts from a *TranscriptDb* object

In order to compute overlaps between reads and transcripts, we need access to the genomic positions of a set of known transcripts and their exons. It is essential that the reference genome of this set of transcripts and exons be **exactly** the same as the reference genome used to align the reads.

We could use the `makeTranscriptDbFromUCSC` function defined in the GenomicFeatures package to make a *TranscriptDb* object containing the dm3 transcripts and their exons retrieved from the UCSC Genome Browser[3]. The Bioconductor project however provides a few annotation packages containing *TranscriptDb* objects for the most commonly studied organisms (those data packages are sometimes called the *TxDb* packages). One of them is the TxDb.Dmelanogaster.UCSC.dm3.ensGene package. It contains a *TranscriptDb* object that was made by pointing the `makeTranscriptDbFromUCSC` function to the dm3 genome and *Ensembl Genes* track [4]. We can use it here:

```
> library(TxDb.Dmelanogaster.UCSC.dm3.ensGene)
> TxDb.Dmelanogaster.UCSC.dm3.ensGene

TranscriptDb object:
| Db type: TranscriptDb
| Supporting package: GenomicFeatures
| Data source: UCSC
| Genome: dm3
| Organism: Drosophila melanogaster
| UCSC Table: ensGene
| Resource URL: http://genome.ucsc.edu/
| Type of Gene ID: Ensembl gene ID
| Full dataset: yes
| miRBase build ID: NA
| transcript_nrow: 23017
| exon_nrow: 69155
| cds_nrow: 59573
| Db created by: GenomicFeatures package from Bioconductor
| Creation time: 2013-03-08 09:48:17 -0800 (Fri, 08 Mar 2013)
| GenomicFeatures version at creation time: 1.11.14
| RSQLite version at creation time: 0.11.2
| DBSCHEMAVERSION: 1.0

> txdb <- TxDb.Dmelanogaster.UCSC.dm3.ensGene
```

[3] http://genome.ucsc.edu/cgi-bin/hgGateway

[4] See http://genome.ucsc.edu/cgi-bin/hgTrackUi?hgsid=276880911&g=ensGene for a description of this track.

We extract the exons grouped by transcript in a *GRangesList* object:

```
> exbytx <- exonsBy(txdb, by="tx", use.names=TRUE)
> length(exbytx)  # nb of transcripts

[1] 23017
```

We check that all the exons in any given transcript belong to the same chromosome and strand. Knowing that our set of transcripts is free of this sort of trans-splicing events typically allows some significant simplifications during the downstream analysis [5]. A quick and easy way to check this is to take advantage of the fact that `seqnames` and `strand` return *RleList* objects. So we can extract the number of Rle runs for each transcript and make sure it's always 1:

```
> table(elementLengths(runLength(seqnames(exbytx))))

    1
23017

> table(elementLengths(runLength(strand(exbytx))))

    1
23017
```

Therefore the strand of any given transcript is unambiguously defined and can be extracted with:

```
> exbytx_strand <- unlist(runValue(strand(exbytx)), use.names=FALSE)
```

We will also need the mapping between the transcripts and their gene. We start by using `transcripts` to extract this information from our *TranscriptDb* object `txdb`, and then we construct a named factor that represents the mapping:

```
> tx <- transcripts(txdb, columns=c("tx_name", "gene_id"))
> head(tx)

GRanges with 6 ranges and 2 metadata columns:
      seqnames         ranges strand |     tx_name         gene_id
         <Rle>      <IRanges>  <Rle> | <character> <CharacterList>
  [1]    chr2L [ 7529,  9484]      + | FBtr0300689     FBgn0031208
  [2]    chr2L [ 7529,  9484]      + | FBtr0300690     FBgn0031208
  [3]    chr2L [67044, 71390]      + | FBtr0078100     FBgn0067779
  [4]    chr2L [72388, 76211]      + | FBtr0078101     FBgn0031213
  [5]    chr2L [73485, 76211]      + | FBtr0302164     FBgn0031213
  [6]    chr2L [74485, 76211]      + | FBtr0301733     FBgn0031213
  ---
  seqlengths:
       chr2L     chr2R     chr3L     chr3R      chr4 ...   chr3RHet    chrXHet    chrYHet chrUextra
    23011544  21146708  24543557  27905053   1351857 ...    2517507     204112     347038  29004656

> df <- mcols(tx)
> exbytx2gene <- as.character(df$gene_id)
> exbytx2gene <- factor(exbytx2gene, levels=unique(exbytx2gene))
> names(exbytx2gene) <- df$tx_name
> exbytx2gene <- exbytx2gene[names(exbytx)]
> head(exbytx2gene)

FBtr0300689 FBtr0300690 FBtr0078100 FBtr0078101 FBtr0302164 FBtr0301733
FBgn0031208 FBgn0031208 FBgn0067779 FBgn0031213 FBgn0031213 FBgn0031213
14869 Levels: FBgn0031208 FBgn0067779 FBgn0031213 FBgn0031214 FBgn0031216 FBgn0031217 ... FBgn0085792

> nlevels(exbytx2gene)  # nb of genes

[1] 14869
```

[5] Dealing with trans-splicing events is not covered in this document.

4.2 Single-end overlaps

4.2.1 Find the single-end overlaps

We are ready to compute the overlaps with the `findOverlaps` function. Note that the strand of the queries produced by the RNA-seq experiment is typically unknown so we use `ignore.strand=TRUE`:

```
> U1.OV00 <- findOverlaps(U1.GAL, exbytx, ignore.strand=TRUE)
```

`U1.OV00` is a *Hits* object that contains 1 element per overlap. Its length gives the number of overlaps:

```
> length(U1.OV00)
```

```
[1] 496485
```

4.2.2 Tabulate the single-end overlaps

We will repeatedly use the 2 following little helper functions to "tabulate" the overlaps in a given *Hits* object (e.g. `U1.OV00`), i.e. to count the number of overlaps for each element in the query or for each element in the subject:

```
> nhitPerQuery <- function(x) tabulate(queryHits(x), nbins=queryLength(x))
> nhitPerSubject <- function(x) tabulate(subjectHits(x), nbins=subjectLength(x))
```

Number of transcripts for each alignment in `U1.GAL`:

```
> U1.GAL_ntx <- nhitPerQuery(U1.OV00)
> mcols(U1.GAL)$ntx <- U1.GAL_ntx
> head(U1.GAL)
```

```
GappedAlignments with 6 alignments and 3 metadata columns:
                   seqnames strand       cigar    qwidth     start       end     width      ngap |
                      <Rle>  <Rle> <character> <integer> <integer> <integer> <integer> <integer> |
  SRR031729.3941844     chr4      -         75M        75       892       966        75         0 |
  SRR031728.3674563     chr4      -         75M        75       919       993        75         0 |
  SRR031729.8532600     chr4      +         75M        75       924       998        75         0 |
  SRR031729.2779333     chr4      +         75M        75       936      1010        75         0 |
  SRR031728.2826481     chr4      +         75M        75       949      1023        75         0 |
  SRR031728.2919098     chr4      -         75M        75       967      1041        75         0 |
                                                                                       seq
                                                                            <DNAStringSet>
  SRR031729.3941844 CTGTGGTGACCAACACCACAGAATGGTTCGGGCCCAATTAGAGGGTTCCCTGCCCCTTTCCTGGCTAGGTTGTCC
  SRR031728.3674563 TCGGGCCCAATTAGAGGGTTCCCTGCCCCTTTCCTGGCTAGGTTGTCCGCTAGCTCATTTCCTGGGCTGTTGTTG
  SRR031729.8532600 CCCAATTAGAGGATTCTCTGCCCCTTTCCTGGCTAGGTTGTCCGGTAGCTCATTTCCCGGGATGTTGTTGTGTCC
  SRR031729.2779333 GTTCTCTGCCCCTTTCCTGGCTAGGTTGTCCGCTAGCTCATTTCCCGAGATGTTGTTGTGTCCCGGGACCCACCT
  SRR031728.2826481 TTCCTGGCTAGGTTGTCCGCTAGCTCATTTCCCGGGCTGTTGTTGTGTCCCGGGACACACCTTATTGTGAGTTTG
  SRR031728.2919098 GCTAGCTCATTTCCCGGGAGGTTGTTGTGTCCCGGGACCCACCTTATTGTGAGTTTGTTGACAGCTCCAAGTTTG
                           NM       ntx
                    <integer> <integer>
  SRR031729.3941844         1         0
  SRR031728.3674563         3         0
  SRR031729.8532600         2         0
  SRR031729.2779333         1         0
  SRR031728.2826481         2         0
  SRR031728.2919098         1         0
  ---
  seqlengths:
       chr2L    chr2R    chr3L    chr3R     chr4     chrM     chrX  chrYHet
    23011544 21146708 24543557 27905053  1351857    19517 22422827   347038
```

```
> table(U1.GAL_ntx)
```

```
U1.GAL_ntx
    0     1     2     3     4     5     6     7     8     9    10 
49079 37954 16036 58831 11042  5683 13877  2897  6914  1890   152 
```

```
> mean(U1.GAL_ntx >= 1)
```

```
[1] 0.7598346
```

76% of the alignments in `U1.GAL` have an overlap with at least 1 transcript in `exbytx`.

Note that `countOverlaps` can be used directly on `U1.GAL` and `exbytx` for computing `U1.GAL_ntx`:

```
> U1.GAL_ntx_again <- countOverlaps(U1.GAL, exbytx, ignore.strand=TRUE)
> stopifnot(identical(unname(U1.GAL_ntx_again), U1.GAL_ntx))
```

Because `U1.GAL` can (and actually does) contain more than 1 alignment per *original query* (aka read), we also count the number of transcripts for each read:

```
> U1.OV10 <- remapHits(U1.OV00, query.map=U1.GAL_qnames)
> U1.uqnames_ntx <- nhitPerQuery(U1.OV10)
> names(U1.uqnames_ntx) <- U1.uqnames
> table(U1.uqnames_ntx)
```

```
U1.uqnames_ntx
    0     1     2     3     4     5     6     7     8     9    10 
41205 30281 16063 59904 11909  5683 13876  2893  6914  1890   152 
```

```
> mean(U1.uqnames_ntx >= 1)
```

```
[1] 0.7840069
```

78.4% of the reads have an overlap with at least 1 transcript in `exbytx`.

Number of reads for each transcript:

```
> U1.exbytx_nOV10 <- nhitPerSubject(U1.OV10)
> names(U1.exbytx_nOV10) <- names(exbytx)
> mean(U1.exbytx_nOV10 >= 50)
```

```
[1] 0.00868923
```

Only 0.869% of the transcripts in `exbytx` have an overlap with at least 50 reads.

Top 10 transcripts:

```
> head(sort(U1.exbytx_nOV10, decreasing=TRUE), n=10)
```

```
FBtr0089175 FBtr0089176 FBtr0089177 FBtr0112904 FBtr0289951 FBtr0089243 FBtr0089186 FBtr0089173 
      40582       40582       40582       11735       11661       11656       10084        6750 
FBtr0089172 FBtr0300498 
       6749        6748 
```

4.3 Paired-end overlaps

4.3.1 Find the paired-end overlaps

Like with our single-end overlaps, we call `findOverlaps` with `ignore.strand=TRUE`:

```
> U3.OV00 <- findOverlaps(U3.GALP, exbytx, ignore.strand=TRUE)
```

Like `U1.OV00`, `U3.OV00` is a *Hits* object. Its length gives the number of paired-end overlaps:

```
> length(U3.OV00)
```

```
[1] 102883
```

4.3.2 Tabulate the paired-end overlaps

Number of transcripts for each alignment pair in U3.GALP:

```
> U3.GALP_ntx <- nhitPerQuery(U3.OV00)
> mcols(U3.GALP)$ntx <- U3.GALP_ntx
> head(U3.GALP)

GappedAlignmentPairs with 6 alignment pairs and 1 metadata column:
                  seqnames strand :       ranges --       ranges |       ntx
                     <Rle>  <Rle> :    <IRanges> --    <IRanges> | <integer>
SRR031715.1138209     chr4      + : [ 169,  205] -- [ 326,  362] |         0
 SRR031714.756385     chr4      + : [ 943,  979] -- [1086, 1122] |         0
SRR031714.5054563     chr4      + : [ 946,  982] -- [ 986, 1022] |         0
SRR031715.1722593     chr4      + : [ 966, 1002] -- [1108, 1144] |         0
SRR031715.2202469     chr4      + : [ 966, 1002] -- [1114, 1150] |         0
SRR031714.3544437     chr4      - : [1087, 1123] -- [ 963,  999] |         0
---
seqlengths:
    chr2L    chr2R    chr3L    chr3R     chr4     chrM     chrX  chrYHet
 23011544 21146708 24543557 27905053  1351857    19517 22422827   347038

> table(U3.GALP_ntx)

U3.GALP_ntx
    0     1     2     3     4     5     6     7     8     9    10    14
13219  8661  3581 11449  2384  1197  2708   423  1905   271    29     1

> mean(U3.GALP_ntx >= 1)

[1] 0.7115519
```

71% of the alignment pairs in U3.GALP have an overlap with at least 1 transcript in exbytx.
Note that countOverlaps can be used directly on U3.GALP and exbytx for computing U3.GALP_ntx:

```
> U3.GALP_ntx_again <- countOverlaps(U3.GALP, exbytx, ignore.strand=TRUE)
> stopifnot(identical(unname(U3.GALP_ntx_again), U3.GALP_ntx))
```

Because U3.GALP can (and actually does) contain more than 1 alignment pair per *original query template*, we also count the number of transcripts for each template:

```
> U3.OV10 <- remapHits(U3.OV00, query.map=U3.GALP_qnames)
> U3.uqnames_ntx <- nhitPerQuery(U3.OV10)
> names(U3.uqnames_ntx) <- U3.uqnames
> table(U3.uqnames_ntx)

U3.uqnames_ntx
    0     1     2     3     4     5     6     7     8     9    10    14
12094  7108  3701 11692  2530  1197  2708   423  1905   271    29     1

> mean(U3.uqnames_ntx >= 1)

[1] 0.7229895
```

72.3% of the templates have an overlap with at least 1 transcript in exbytx.
Number of templates for each transcript:

```
> U3.exbytx_nOV10 <- nhitPerSubject(U3.OV10)
> names(U3.exbytx_nOV10) <- names(exbytx)
> mean(U3.exbytx_nOV10 >= 50)

[1] 0.00755963
```

Only 0.756% of the transcripts in `exbytx` have an overlap with at least 50 templates.

Top 10 transcripts:

```
> head(sort(U3.exbytx_nOV10, decreasing=TRUE), n=10)

FBtr0089175 FBtr0089176 FBtr0089177 FBtr0112904 FBtr0089243 FBtr0289951 FBtr0089186 FBtr0089100
       7573        7572        7556        2750        2732        2732        2260        1691
FBtr0089099 FBtr0089098
       1691        1691
```

5 Encode the overlaps between the reads and transcripts

5.1 Single-end encodings

The *overlap encodings* are strand sensitive so we will compute them twice, once for the "original alignments" (i.e. the alignments of the *original queries*), and once again for the "flipped alignments" (i.e. the alignments of the "flipped *original queries*"). We extract the ranges of the "original" and "flipped" alignments in 2 *GRangesList* objects with:

```
> U1.grlf <- flipQuery(U1.grl)  # flipped
```

and encode their overlaps with the transcripts:

```
> U1.ovencA <- encodeOverlaps(U1.grl, exbytx, hits=U1.OV00)
> U1.ovencB <- encodeOverlaps(U1.grlf, exbytx, hits=U1.OV00)
```

`U1.ovencA` and `U1.ovencB` are 2 *OverlapsEncodings* objects of the same length as *Hits* object `U1.OV00`. For each hit in `U1.OV00`, we have 2 corresponding encodings, one in `U1.ovencA` and one in `U1.ovencB`, but only one of them encodes a hit between alignment ranges and exon ranges that are on the same strand. We use the `selectEncodingWithCompatibleStrand` function to merge them into a single *OverlapsEncodings* of the same length. For each hit in `U1.OV00`, this selects the encoding corresponding to alignment ranges and exon ranges with compatible strand:

```
> U1.grl_strand <- unlist(runValue(strand(U1.grl)), use.names=FALSE)
> U1.ovenc <- selectEncodingWithCompatibleStrand(U1.ovencA, U1.ovencB,
+                                                U1.grl_strand, exbytx_strand,
+                                                hits=U1.OV00)
> U1.ovenc

OverlapEncodings of length 496485
         Loffset Roffset encoding flippedQuery
[1]            0       3     1:i:         TRUE
[2]            6       0     1:i:         TRUE
[3]            6       0     1:i:         TRUE
[4]            6       0     1:i:         TRUE
[5]            6       0     1:i:         TRUE
[6]            6       0     1:i:        FALSE
[7]            6       0     1:i:         TRUE
[8]            6       0     1:i:         TRUE
[9]            6       0     1:i:         TRUE
...          ...     ...      ...          ...
[496477]      23       0     1:i:         TRUE
[496478]      24       0     1:i:         TRUE
[496479]      22       0     1:i:         TRUE
[496480]      23       0     1:i:        FALSE
[496481]      24       0     1:i:        FALSE
[496482]      22       0     1:i:        FALSE
[496483]      23       0     1:i:         TRUE
[496484]      24       0     1:i:         TRUE
[496485]      22       0     1:i:         TRUE
```

As a convenience, the 2 above calls to `encodeOverlaps` + merging step can be replaced by a single call to `encodeOverlaps` on `U1.grl` (or `U1.grlf`) with `flip.query.if.wrong.strand=TRUE`:

```
> U1.ovenc_again <- encodeOverlaps(U1.grl, exbytx, hits=U1.OV00, flip.query.if.wrong.strand=TRUE)
> stopifnot(identical(U1.ovenc_again, U1.ovenc))
```

Unique encodings in `U1.ovenc`:

```
> U1.unique_encodings <- levels(U1.ovenc)
> length(U1.unique_encodings)

[1] 116

> head(U1.unique_encodings)

[1] "1:c:" "1:e:" "1:f:" "1:h:" "1:i:" "1:j:"

> U1.ovenc_table <- table(encoding(U1.ovenc))
> tail(sort(U1.ovenc_table))

    1:j:   1:k:c:     1:k:     1:c: 2:jm:af:     1:i:
    1384     1919    10103    12659    65169   395278
```

Encodings are sort of cryptic but utilities are provided to extract specific meaning from them. Use of these utilities is covered later in this document.

5.2 Paired-end encodings

Let's encode the overlaps in `U3.OV00`:

```
> U3.grl <- grglist(U3.GALP, order.as.in.query=TRUE)
> U3.ovenc <- encodeOverlaps(U3.grl, exbytx, hits=U3.OV00, flip.query.if.wrong.strand=TRUE)
> U3.ovenc

OverlapEncodings of length 102883
         Loffset Roffset          encoding flippedQuery
[1]            6       0        1--1:i--m:        FALSE
[2]            5       1        1--1:i--i:        FALSE
[3]            4       2        1--1:i--i:         TRUE
[4]            4       2        1--1:i--i:         TRUE
[5]            4       2        1--1:i--i:         TRUE
[6]            4       2        1--1:i--i:         TRUE
[7]            4       2        1--1:i--i:        FALSE
[8]            4       2        1--1:i--i:        FALSE
[9]            4       2        1--1:i--i:        FALSE
...          ...     ...               ...          ...
[102875]      22       0 2--1:jm--m:af--i:        FALSE
[102876]      23       0 2--1:jm--m:af--i:        FALSE
[102877]      21       0 2--1:jm--m:af--i:        FALSE
[102878]      23       0        1--1:i--i:        FALSE
[102879]      24       0        1--1:i--i:        FALSE
[102880]      22       0        1--1:i--i:        FALSE
[102881]      23       0        1--1:i--i:         TRUE
[102882]      24       0        1--1:i--i:         TRUE
[102883]      22       0        1--1:i--i:         TRUE
```

Unique encodings in `U3.ovenc`:

```
> U3.unique_encodings <- levels(U3.ovenc)
> length(U3.unique_encodings)

[1] 123

> head(U3.unique_encodings)
```

```
[1] "1--1:a--c:" "1--1:a--e:" "1--1:a--f:" "1--1:a--i:" "1--1:a--j:" "1--1:a--k:"
```

```
> U3.ovenc_table <- table(encoding(U3.ovenc))
> tail(sort(U3.ovenc_table))
```

```
      1--1:i--k:       1--1:i--m:        1--1:c--i: 1--2:i--jm:a--af: 2--1:jm--m:af--i:
            1531             1534              1778              2023              2562
      1--1:i--i:
           87951
```

6 "Compatible" overlaps

We are interested in a particular type of overlap where the read overlaps the transcript in a "compatible" way, that is, in a way compatible with the splicing of the transcript. The `isCompatibleWithSplicing` function can be used on an *OverlapEncodings* object to detect this type of overlap. Note that `isCompatibleWithSplicing` can also be used on a character vector or factor.

6.1 "Compatible" single-end overlaps

6.1.1 "Compatible" single-end encodings

`U1.ovenc` contains 7 unique encodings "compatible" with the splicing of the transcript:

```
> sort(U1.ovenc_table[isCompatibleWithSplicing(U1.unique_encodings)])
```

```
     2:jm:ag:       2:gm:af: 3:jmm:agm:aaf:            1:f:            1:j:       2:jm:af:
           25             54            342            1336            1384          65169
         1:i:
       395278
```

Encodings `"1:i:"` (395278 occurences in `U1.ovenc`), `"2:jm:af:"` (65169 occurences in `U1.ovenc`), and `"3:jmm:agm:aaf:"` (342 occurences in `U1.ovenc`), correspond to the following overlaps:

- `"1:i:"`

```
  - read (no gap):            oooooooo
  - transcript:     ...   >>>>>>>>>>>>>>   ...
```

- `"2:jm:af:"`

```
  - read (1 gap):             ooooo---ooo
  - transcript:     ...   >>>>>>>>>   >>>>>>>>>   ...
```

- `"3:jmm:agm:aaf:"`

```
  - read (2 gaps):              oo---ooooo---o
  - transcript:     ...   >>>>>>>>   >>>>>   >>>>>>>   ...
```

For clarity, only the exons involved in the overlap are represented. The transcript can of course have more upstream and downstream exons, which is denoted by the ... on the left side (5' end) and right side (3' end) of each drawing. Note that the exons represented in the 2nd and 3rd drawings are consecutive in the transcript.

Encodings `"1:f:"` and `"1:j:"` are variations of the situation described by encoding `"1:i:"`. For `"1:f:"`, the first aligned base of the read (or "flipped" read) is aligned with the first base of the exon. For `"1:j:"`, the last aligned base of the read (or "flipped" read) is aligned with the last base of the exon:

- `"1:f:"`

```
  - read (no gap):        oooooooo
  - transcript:     ...   >>>>>>>>>>>>>>   ...
```

• "1:j:"

```
    - read (no gap):              oooooooo
    - transcript:     ...   >>>>>>>>>>>>>>>   ...
```

```
> U1.OV00_is_comp <- isCompatibleWithSplicing(U1.ovenc)
> table(U1.OV00_is_comp)  # 476124 "compatible" overlaps
```

```
U1.OV00_is_comp
 FALSE   TRUE 
 32897 463588 
```

Finally, let's extract the "compatible" overlaps from `U1.OV00`:

```
> U1.compOV00 <- U1.OV00[U1.OV00_is_comp]
```

Note that high-level convenience wrapper `findCompatibleOverlaps` can be used for computing the "compatible" overlaps directly between a *GappedAlignments* object (containing reads) and a *GRangesList* object (containing transcripts):

```
> U1.compOV00_again <- findCompatibleOverlaps(U1.GAL, exbytx)
> stopifnot(identical(U1.compOV00_again, U1.compOV00))
```

6.1.2 Tabulate the "compatible" single-end overlaps

Number of "compatible" transcripts for each alignment in `U1.GAL`:

```
> U1.GAL_ncomptx <- nhitPerQuery(U1.compOV00)
> mcols(U1.GAL)$ncomptx <- U1.GAL_ncomptx
> head(U1.GAL)
```

```
GappedAlignments with 6 alignments and 4 metadata columns:
                    seqnames strand       cigar    qwidth     start       end     width      ngap |
                       <Rle>  <Rle> <character> <integer> <integer> <integer> <integer> <integer> |
  SRR031729.3941844     chr4      -         75M        75       892       966        75         0 |
  SRR031728.3674563     chr4      -         75M        75       919       993        75         0 |
  SRR031729.8532600     chr4      +         75M        75       924       998        75         0 |
  SRR031729.2779333     chr4      +         75M        75       936      1010        75         0 |
  SRR031728.2826481     chr4      +         75M        75       949      1023        75         0 |
  SRR031728.2919098     chr4      -         75M        75       967      1041        75         0 |
                                                                                        seq
                                                                             <DNAStringSet>
  SRR031729.3941844 CTGTGGTGACCAACACCACAGAATGGTTCGGGCCCAATTAGAGGGTTCCCTGCCCCTTTCCTGGCTAGGTTGTCC
  SRR031728.3674563 TCGGGCCCAATTAGAGGGTTCCCTGCCCCTTTCCTGGCTAGGTTGTCCGCTAGCTCATTTCCTGGGCTGTTGTTG
  SRR031729.8532600 CCCAATTAGAGGATTCTCTGCCCCTTTCCTGGCTAGGTTGTCCGGTAGCTCATTTCCCGGGATGTTGTTGTGTCC
  SRR031729.2779333 GTTCTCTGCCCCTTTCCTGGCTAGGTTGTCCGCTAGCTCATTTCCCGAGATGTTGTTGTGTCCCGGGACCCACCT
  SRR031728.2826481 TTCCTGGCTAGGTTGTCCGCTAGCTCATTTCCCGGGCTGTTGTTGTGTCCCGGGACACACCTTATTGTGAGTTTG
  SRR031728.2919098 GCTAGCTCATTTCCCGGGAGGTTGTTGTGTCCCGGGACCCACCTTATTGTGAGTTTGTTGACAGCTCCAAGTTTG
                           NM       ntx   ncomptx
                    <integer> <integer> <integer>
  SRR031729.3941844         1         0         0
  SRR031728.3674563         3         0         0
  SRR031729.8532600         2         0         0
  SRR031729.2779333         1         0         0
  SRR031728.2826481         2         0         0
  SRR031728.2919098         1         0         0
  ---
  seqlengths:
      chr2L    chr2R    chr3L    chr3R     chr4     chrM     chrX  chrYHet
   23011544 21146708 24543557 27905053  1351857    19517 22422827   347038
```

```
> table(U1.GAL_ncomptx)

U1.GAL_ncomptx
    0     1     2     3     4     5     6     7     8     9    10
53514 43731 16616 50092 10949  5404 13088  2502  6688  1723    48

> mean(U1.GAL_ncomptx >= 1)

[1] 0.7381322
```

73.8% of the alignments in U1.GAL are "compatible" with at least 1 transcript in exbytx.

Note that high-level convenience wrapper countCompatibleOverlaps can be used directly on U1.GAL and exbytx for computing U1.GAL_ncomptx:

```
> U1.GAL_ncomptx_again <- countCompatibleOverlaps(U1.GAL, exbytx)
> stopifnot(identical(U1.GAL_ncomptx_again, U1.GAL_ncomptx))
```

Number of "compatible" transcripts for each read:

```
> U1.compOV10 <- remapHits(U1.compOV00, query.map=U1.GAL_qnames)
> U1.uqnames_ncomptx <- nhitPerQuery(U1.compOV10)
> names(U1.uqnames_ncomptx) <- U1.uqnames
> table(U1.uqnames_ncomptx)

U1.uqnames_ncomptx
    0     1     2     3     4     5     6     7     8     9    10
44832 36563 17029 51158 11735  5404 13088  2502  6688  1723    48

> mean(U1.uqnames_ncomptx >= 1)

[1] 0.7649945
```

76.5% of the reads are "compatible" with at least 1 transcript in exbytx.

Number of "compatible" reads for each transcript:

```
> U1.exbytx_ncompOV10 <- nhitPerSubject(U1.compOV10)
> names(U1.exbytx_ncompOV10) <- names(exbytx)
> mean(U1.exbytx_ncompOV10 >= 50)

[1] 0.008602337
```

Only 0.860% of the transcripts in exbytx are "compatible" with at least 50 reads.

Top 10 transcripts:

```
> head(sort(U1.exbytx_ncompOV10, decreasing=TRUE), n=10)

FBtr0089175 FBtr0089177 FBtr0089176 FBtr0089243 FBtr0289951 FBtr0112904 FBtr0089186 FBtr0089173
      40289       33978       33621       11365       11332       11284       10025        6606
FBtr0300497 FBtr0089172
       6601        6599
```

Note that this "top 10" is slightly different from the "top 10" we obtained earlier when we counted **all** the overlaps.

6.2 "Compatible" paired-end overlaps

6.2.1 "Compatible" paired-end encodings

U3.ovenc contains 13 unique paired-end encodings "compatible" with the splicing of the transcript:

```
> sort(U3.ovenc_table[isCompatibleWithSplicing(U3.unique_encodings)])
```

```
         1--2:f--jm:a--af:              1--1:f--j:         2--1:jm--m:af--j:
                         3                      9                        12
         2--1:jm--m:af--f:          1--1:i--m:a--f:          1--1:j--m:a--i:
                        24                     48                        56
2--2:jm--mm:af--jm:aa--af:          1--1:i--m:a--i:               1--1:i--j:
                       122                    353                       361
              1--1:f--i:         1--2:i--jm:a--af:         2--1:jm--m:af--i:
                       544                   2023                      2562
              1--1:i--i:
                     87951
```

Paired-end encodings "1--1:i--i:" (87951 occurences in U3.ovenc), "2--1:jm--m:af--i:" (2562 occurences in U3.ovenc), "1--2:i--jm:a--af:" (2023 occurences in U3.ovenc), "1--1:i--m:a--i:" (353 occurences in U3.ovenc), and "2--2:jm--mm:af--jm:aa--af:" (122 occurences in U3.ovenc), correspond to the following paired-end overlaps:

- "1--1:i--i:"

  ```
  - paired-end read (no gap on the first end, no gap on the
    last end):               oooo   oooo
  - transcript:     ...   >>>>>>>>>>>>>>>>   ...
  ```

- "2--1:jm--m:af--i:"

  ```
  - paired-end read (1 gap on the first end, no gap on the
    last end):                 ooo---o    oooo
  - transcript:     ...   >>>>>>>>   >>>>>>>>>>>   ...
  ```

- "1--2:i--jm:a--af:"

  ```
  - paired-end read (no gap on the first end, 1 gap on the
    last end):             oooo      oo---oo
  - transcript:     ...  >>>>>>>>>>>>>>   >>>>>>>>>  ...
  ```

- "1--1:i--m:a--i:"

  ```
  - paired-end read (no gap on the first end, no gap on the
    last end):               oooo      oooo
  - transcript:     ...   >>>>>>>>>   >>>>>>>   ...
  ```

- "2--2:jm--mm:af--jm:aa--af:"

  ```
  - paired-end read (1 gap on the first end, 1 gap on the
    last end):               ooo---o    oo---oo
  - transcript:     ...   >>>>>>   >>>>>>>   >>>>>   ...
  ```

Note: switch use of "first" and "last" above if the read was "flipped".

```
> U3.OVOO_is_comp <- isCompatibleWithSplicing(U3.ovenc)
> table(U3.OVOO_is_comp)  # 94068 "compatible" paired-end overlaps

U3.OVOO_is_comp
FALSE  TRUE
 8815 94068
```

Finally, let's extract the "compatible" paired-end overlaps from U3.OVOO:

```
> U3.compOVOO <- U3.OVOO[U3.OVOO_is_comp]
```

Note that, like with our single-end reads, high-level convenience wrapper findCompatibleOverlaps can be used for computing the "compatible" paired-end overlaps directly between a *GappedAlignmentPairs* object (containing paired-end reads) and a *GRangesList* object (containing transcripts):

```
> U3.compOVOO_again <- findCompatibleOverlaps(U3.GALP, exbytx)
> stopifnot(identical(U3.compOVOO_again, U3.compOVOO))
```

6.2.2 Tabulate the "compatible" paired-end overlaps

Number of "compatible" transcripts for each alignment pair in `U3.GALP`:

```
> U3.GALP_ncomptx <- nhitPerQuery(U3.compOV00)
> mcols(U3.GALP)$ncomptx <- U3.GALP_ncomptx
> head(U3.GALP)

GappedAlignmentPairs with 6 alignment pairs and 2 metadata columns:
                  seqnames strand :       ranges --       ranges |       ntx   ncomptx
                     <Rle>  <Rle> :    <IRanges> --    <IRanges> | <integer> <integer>
SRR031715.1138209     chr4      + : [ 169,  205] -- [ 326,  362] |         0         0
 SRR031714.756385     chr4      + : [ 943,  979] -- [1086, 1122] |         0         0
SRR031714.5054563     chr4      + : [ 946,  982] -- [ 986, 1022] |         0         0
SRR031715.1722593     chr4      + : [ 966, 1002] -- [1108, 1144] |         0         0
SRR031715.2202469     chr4      + : [ 966, 1002] -- [1114, 1150] |         0         0
SRR031714.3544437     chr4      - : [1087, 1123] -- [ 963,  999] |         0         0
---
seqlengths:
    chr2L    chr2R    chr3L    chr3R     chr4     chrM     chrX  chrYHet
 23011544 21146708 24543557 27905053  1351857    19517 22422827   347038

> table(U3.GALP_ncomptx)

U3.GALP_ncomptx
    0     1     2     3     4     5     6     7     8     9    10
14242 10842  3569  8652  2345  1135  2589   356  1833   263     2

> mean(U3.GALP_ncomptx >= 1)

[1] 0.6892293
```

68.9% of the alignment pairs in `U3.GALP` are "compatible" with at least 1 transcript in `exbytx`.

Note that high-level convenience wrapper `countCompatibleOverlaps` can be used directly on `U3.GALP` and `exbytx` for computing `U3.GALP_ncomptx`:

```
> U3.GALP_ncomptx_again <- countCompatibleOverlaps(U3.GALP, exbytx)
> stopifnot(identical(U3.GALP_ncomptx_again, U3.GALP_ncomptx))
```

Number of "compatible" transcripts for each template:

```
> U3.compOV10 <- remapHits(U3.compOV00, query.map=U3.GALP_qnames)
> U3.uqnames_ncomptx <- nhitPerQuery(U3.compOV10)
> names(U3.uqnames_ncomptx) <- U3.uqnames
> table(U3.uqnames_ncomptx)

U3.uqnames_ncomptx
    0     1     2     3     4     5     6     7     8     9    10
13044  9376  3695  8914  2452  1135  2589   356  1833   263     2

> mean(U3.uqnames_ncomptx >= 1)

[1] 0.70123
```

70.1% of the templates are "compatible" with at least 1 transcript in `exbytx`.

Number of "compatible" templates for each transcript:

```
> U3.exbytx_ncompOV10 <- nhitPerSubject(U3.compOV10)
> names(U3.exbytx_ncompOV10) <- names(exbytx)
> mean(U3.exbytx_ncompOV10 >= 50)

[1] 0.007516184
```

Only 0.752% of the transcripts in exbytx are "compatible" with at least 50 templates.
Top 10 transcripts:

```
> head(sort(U3.exbytx_ncompOV10, decreasing=TRUE), n=10)
```

```
FBtr0089175 FBtr0089176 FBtr0089177 FBtr0289951 FBtr0089243 FBtr0112904 FBtr0089186 FBtr0089096
       7490        5252        5234        2686        2684        2640        2246        1638
FBtr0089100 FBtr0089099
       1632        1632
```

Note that this "top 10" is slightly different from the "top 10" we obtained earlier when we counted **all** the paired-end overlaps.

7 Project the alignments on the transcriptome

7.1 Project the single-end alignments on the transcriptome

The extractQueryStartInTranscript function computes for each overlap the position of the *query start* in the transcript:

```
> U1.OV00_qstart <- extractQueryStartInTranscript(U1.grl, exbytx,
+                                                 hits=U1.OV00, ovenc=U1.ovenc)
> head(subset(U1.OV00_qstart, U1.OV00_is_comp))
```

```
  startInTranscript firstSpannedExonRank startInFirstSpannedExon
1               100                    1                     100
2              3584                    7                      26
3              3579                    7                      21
4              3579                    7                      21
5              3576                    7                      18
6              3574                    7                      16
```

U1.OV00_qstart is a data frame with 1 row per overlap and 3 columns:

1. startInTranscript: the 1-based start position of the read with respect to the transcript. Position 1 always corresponds to the first base on the 5' end of the transcript sequence.
2. firstSpannedExonRank: the rank of the first exon spanned by the read, that is, the rank of the exon found at position startInTranscript in the transcript.
3. startInFirstSpannedExon: the 1-based start position of the read with respect to the first exon spanned by the read.

Having this information allows us for example to compare the read and transcript nucleotide sequences for each "compatible" overlap. If we use the *reference query sequence* instead of the *original query sequence* for this comparison, then it should match **exactly** the sequence found at the *query start* in the transcript.

Let's start by using the extractTranscriptsFromGenome to extract the transcript sequences (aka transcriptome) from the dm3 reference genome:

```
> txseq <- extractTranscriptsFromGenome(Dmelanogaster, exbytx)
```

For each "compatible" overlap, the read sequence in U1.GAL_rqseq must be an *exact* substring of the transcript sequence in exbytx_seq:

```
> U1.OV00_rqseq <- U1.GAL_rqseq[queryHits(U1.OV00)]
> U1.OV00_rqseq[flippedQuery(U1.ovenc)] <- reverseComplement(U1.OV00_rqseq[flippedQuery(U1.ovenc)])
> U1.OV00_txseq <- txseq[subjectHits(U1.OV00)]
> stopifnot(all(
+     U1.OV00_rqseq[U1.OV00_is_comp] ==
+         narrow(U1.OV00_txseq[U1.OV00_is_comp],
+                start=U1.OV00_qstart$startInTranscript[U1.OV00_is_comp],
+                width=width(U1.OV00_rqseq)[U1.OV00_is_comp])
+ ))
```

Because of this relationship between the *reference query sequence* and the transcript sequence of a "compatible" overlap, and because of the relationship between the *original query sequences* and the *reference query sequences*, then the edit distance reported in the NM tag is actually the edit distance between the *original query* and the transcript of a "compatible" overlap.

7.2 Project the paired-end alignments on the transcriptome

For a paired-end read, the *query start* is the start of its "left end".

```
> U3.OV00_Lqstart <- extractQueryStartInTranscript(U3.grl, exbytx,
+                                                   hits=U3.OV00, ovenc=U3.ovenc)
> head(subset(U3.OV00_Lqstart, U3.OV00_is_comp))

  startInTranscript firstSpannedExonRank startInFirstSpannedExon
2              3406                    6                      31
3              3158                    5                      22
4              3156                    5                      20
5              3140                    5                       4
6              3148                    5                      12
7              3174                    5                      38
```

Note that `extractQueryStartInTranscript` can be called with `for.query.right.end=TRUE` if we want this information for the "right ends" of the reads:

```
> U3.OV00_Rqstart <- extractQueryStartInTranscript(U3.grl, exbytx,
+                                                   hits=U3.OV00, ovenc=U3.ovenc,
+                                                   for.query.right.end=TRUE)
> head(subset(U3.OV00_Rqstart, U3.OV00_is_comp))

  startInTranscript firstSpannedExonRank startInFirstSpannedExon
2              3414                    6                      39
3              3315                    5                     179
4              3297                    5                     161
5              3297                    5                     161
6              3291                    5                     155
7              3289                    5                     153
```

Like with single-end reads, having this information allows us for example to compare the read and transcript nucleotide sequences for each "compatible" overlap. If we use the *reference query sequence* instead of the *original query sequence* for this comparison, then it should match **exactly** the sequences of the "left" and "right" ends of the read in the transcript.

Let's assign the "left and right reference query sequences" to each overlap:

```
> U3.OV00_Lrqseq <- U3.GALP_rqseq1[queryHits(U3.OV00)]
> U3.OV00_Rrqseq <- U3.GALP_rqseq2[queryHits(U3.OV00)]
```

For the single-end reads, the sequence associated with a "flipped query" just needed to be "reverse complemented". For paired-end reads, we also need to swap the 2 sequences in the pair:

```
> flip_idx <- which(flippedQuery(U3.ovenc))
> tmp <- U3.OV00_Lrqseq[flip_idx]
> U3.OV00_Lrqseq[flip_idx] <- reverseComplement(U3.OV00_Rrqseq[flip_idx])
> U3.OV00_Rrqseq[flip_idx] <- reverseComplement(tmp)
```

Let's assign the transcript sequence to each overlap:

```
> U3.OV00_txseq <- txseq[subjectHits(U3.OV00)]
```

For each "compatible" overlap, we expect the "left and right reference query sequences" of the read to be *exact* substrings of the transcript sequence. Let's check the "left reference query sequences":

```
> stopifnot(all(
+     U3.OVOO_Lrqseq[U3.OVOO_is_comp] ==
+         narrow(U3.OVOO_txseq[U3.OVOO_is_comp],
+                start=U3.OVOO_Lqstart$startInTranscript[U3.OVOO_is_comp],
+                width=width(U3.OVOO_Lrqseq)[U3.OVOO_is_comp])
+ ))
```

and the “right reference query sequences”:

```
> stopifnot(all(
+     U3.OVOO_Rrqseq[U3.OVOO_is_comp] ==
+         narrow(U3.OVOO_txseq[U3.OVOO_is_comp],
+                start=U3.OVOO_Rqstart$startInTranscript[U3.OVOO_is_comp],
+                width=width(U3.OVOO_Rrqseq)[U3.OVOO_is_comp])
+ ))
```

8 Align the reads to the transcriptome

Aligning the reads to the reference genome is not the most efficient nor accurate way to count the number of “compatible” overlaps per *original query*. Supporting junction reads (i.e. reads that align with at least 1 gap) introduces a significant computational cost during the alignment process. Then, as we’ve seen in the previous sections, each alignment produced by the aligner needs to be broken into a set of ranges (based on its CIGAR) and those ranges compared to the ranges of the exons grouped by transcript.

A more straightforward and accurate approach is to align the reads directly to the transcriptome, and without allowing the typical gap that the aligner needs to introduce when aligning a junction read to the reference genome. With this approach, a “hit” between a read and a transcript is necessarily compatible with the splicing of the transcript. In case of a “hit”, we’ll say that the read and the transcript are “string-based compatible” (to differentiate from our previous notion of “compatible” overlaps that we will call “encoding-based compatible” from now on, unless the context is clear).

8.1 Align the single-end reads to the transcriptome

8.1.1 Find the “hits”

The single-end reads are in `U1.oqseq`, the transcriptome is in `exbytx_seq`.

Since indels were not allowed/supported during the alignment of the reads to the reference genome, we don’t need to allow/support them either for aligning the reads to the transcriptome. Also since our goal is to find (and count) “compatible” overlaps between reads and transcripts, we don’t need to keep track of the details of the alignments between the reads and the transcripts. Finally, since BAM file `untreated1_chr4.bam` is not the full output of the aligner but the subset obtained by keeping only the alignments located on chr4, we don’t need to align `U1.oqseq` to the full transcriptome, but only to the subset of `exbytx_seq` made of the transcripts located on chr4.

With those simplifications in mind, we write the following function that we will use to find the “hits” between the reads and the transcriptome:

```
> ### A wrapper to vwhichPDict() that supports IUPAC ambiguity codes in 'qseq'
> ### and 'txseq', and treats them as such.
> findSequenceHits <- function(qseq, txseq, which.txseq=NULL, max.mismatch=0)
+ {
+     .asHits <- function(x, pattern_length)
+     {
+         query_hits <- unlist(x)
+         if (is.null(query_hits))
+             query_hits <- integer(0)
+         subject_hits <- rep.int(seq_len(length(x)), elementLengths(x))
+         new("Hits", queryHits=query_hits, subjectHits=subject_hits,
+                     queryLength=pattern_length, subjectLength=length(x))
+     }
+
+     .isHitInTranscriptBounds <- function(hits, qseq, txseq)
```

```
+     {
+         sapply(seq_len(length(hits)),
+             function(i) {
+                 pattern <- qseq[[queryHits(hits)[i]]]
+                 subject <- txseq[[subjectHits(hits)[i]]]
+                 v <- matchPattern(pattern, subject,
+                                   max.mismatch=max.mismatch, fixed=FALSE)
+                 any(1L <= start(v) & end(v) <= length(subject))
+             })
+     }
+
+     if (!is.null(which.txseq)) {
+         txseq0 <- txseq
+         txseq <- txseq[which.txseq]
+     }
+
+     names(qseq) <- NULL
+     other <- alphabetFrequency(qseq, baseOnly=TRUE)[ , "other"]
+     is_clean <- other == 0L  # "clean" means "no IUPAC ambiguity code"
+
+     ## Find hits for "clean" original queries.
+     qseq0 <- qseq[is_clean]
+     pdict0 <- PDict(qseq0, max.mismatch=max.mismatch)
+     m0 <- vwhichPDict(pdict0, txseq,
+                       max.mismatch=max.mismatch, fixed="pattern")
+     hits0 <- .asHits(m0, length(qseq0))
+     hits0@queryLength <- length(qseq)
+     hits0@queryHits <- which(is_clean)[hits0@queryHits]
+
+     ## Find hits for non "clean" original queries.
+     qseq1 <- qseq[!is_clean]
+     m1 <- vwhichPDict(qseq1, txseq,
+                       max.mismatch=max.mismatch, fixed=FALSE)
+     hits1 <- .asHits(m1, length(qseq1))
+     hits1@queryLength <- length(qseq)
+     hits1@queryHits <- which(!is_clean)[hits1@queryHits]
+
+     ## Combine the hits.
+     query_hits <- c(queryHits(hits0), queryHits(hits1))
+     subject_hits <- c(subjectHits(hits0), subjectHits(hits1))
+
+     if (!is.null(which.txseq)) {
+         ## Remap the hits.
+         txseq <- txseq0
+         subject_hits <- which.txseq[subject_hits]
+         hits0@subjectLength <- length(txseq)
+     }
+
+     ## Order the hits.
+     oo <- IRanges:::orderIntegerPairs(query_hits, subject_hits)
+     hits0@queryHits <- query_hits[oo]
+     hits0@subjectHits <- subject_hits[oo]
+
+     if (max.mismatch != 0L) {
+         ## Keep only "in bounds" hits.
+         is_in_bounds <- .isHitInTranscriptBounds(hits0, qseq, txseq)
+         hits0 <- hits0[is_in_bounds]
+     }
```

```
+     hits0
+ }
```

Let's compute the index of the transcripts in `exbytx_seq` located on chr4 (`findSequenceHits` will restrict the search to those transcripts):

```
> chr4tx <- transcripts(txdb, vals=list(tx_chrom="chr4"))
> chr4txnames <- mcols(chr4tx)$tx_name
> which.txseq <- match(chr4txnames, names(txseq))
```

We know that the aligner tolerated up to 6 mismatches per read. The 3 following commands find the "hits" for each *original query*, then find the "hits" for each "flipped *original query*", and finally merge all the "hits" (note that the 3 commands take about 1 hour to complete on a modern laptop):

```
> U1.sbcompHITSa <- findSequenceHits(U1.oqseq, txseq,
+                                    which.txseq=which.txseq, max.mismatch=6)
> U1.sbcompHITSb <- findSequenceHits(reverseComplement(U1.oqseq), txseq,
+                                    which.txseq=which.txseq, max.mismatch=6)
> U1.sbcompHITS <- union(U1.sbcompHITSa, U1.sbcompHITSb)
```

8.1.2 Tabulate the "hits"

Number of "string-based compatible" transcripts for each read:

```
> U1.uqnames_nsbcomptx <- nhitPerQuery(U1.sbcompHITS)
> names(U1.uqnames_nsbcomptx) <- U1.uqnames
> table(U1.uqnames_nsbcomptx)

U1.uqnames_nsbcomptx
    0     1     2     3     4     5     6     7     8     9    10
42494 36484 16641 52677 12214  5542 13446  2642  6777  1787    66

> mean(U1.uqnames_nsbcomptx >= 1)

[1] 0.7772501
```

77.7% of the reads are "string-based compatible" with at least 1 transcript in `exbytx`.
Number of "string-based compatible" reads for each transcript:

```
> U1.exbytx_nsbcompHITS <- nhitPerSubject(U1.sbcompHITS)
> names(U1.exbytx_nsbcompHITS) <- names(exbytx)
> mean(U1.exbytx_nsbcompHITS >= 50)

[1] 0.008645784
```

Only 0.865% of the transcripts in `exbytx` are "string-based compatible" with at least 50 reads.
Top 10 transcripts:

```
> head(sort(U1.exbytx_nsbcompHITS, decreasing=TRUE), n=10)

FBtr0301886 FBtr0078118 FBtr0301887 FBtr0077816 FBtr0077815 FBtr0077817 FBtr0077837 FBtr0078047
      40520       34703       34406       11605       11579       11548       10066        6710
FBtr0078128 FBtr0078131
       6704        6701
```

8.1.3 A closer look at the "hits"

[WORK IN PROGRESS, might be removed or replaced soon...]
Any "encoding-based compatible" overlap is of course "string-based compatible":

```
> ## FIXME: 11 September, 2012 requires U1.sbcompHITS object update
> ## stopifnot(length(setdiff(U1.compOV10, U1.sbcompHITS)) == 0)
```

but the reverse is not true:

```
> length(setdiff(U1.sbcompHITS, U1.compOV10))

[1] 474492
```

8.2 Align the paired-end reads to the transcriptome

[COMING SOON...]

9 "Almost compatible" overlaps

In many aspects, "compatible" overlaps can be seen as perfect. We are now insterested in a less perfect type of overlap where the read overlaps the transcript in a way that *would* be "compatible" if 1 or more exons were removed from the transcript. In that case we say that the overlap is "almost compatible" with the transcript. The `isCompatibleWithSkippedExons` function can be used on an *OverlapEncodings* object to detect this type of overlap. Note that `isCompatibleWithSkippedExons` can also be used on a character vector of factor.

9.1 "Almost compatible" single-end overlaps

9.1.1 "Almost compatible" single-end encodings

`U1.ovenc` contains 7 unique encodings "almost compatible" with the splicing of the transcript:

```
> sort(U1.ovenc_table[isCompatibleWithSkippedExons(U1.unique_encodings)])

2:jm:am:am:am:am:am:af:       2:jm:am:am:am:af:             2:gm:am:af:  3:jmm:agm:aam:aam:aaf:
                      1                       2                       3                       3
     3:jmm:agm:aam:aaf:          2:jm:am:am:af:             2:jm:am:af:
                     18                     112                     721
```

Encodings `"2:jm:am:af:"` (721 occurences in `U1.ovenc`), `"2:jm:am:am:af:"` (112 occurences in `U1.ovenc`), and `"3:jmm:agm:aam:aaf:"` (18 occurences in `U1.ovenc`), correspond to the following overlaps:

- `"2:jm:am:af:"`

```
    - read (1 gap):           ooooo----------ooo
    - transcript:     ...   >>>>>>>   >>>>   >>>>>>>>   ...
```

- `"2:jm:am:am:af:"`

```
    - read (1 gap):           ooooo------------------ooo
    - transcript:     ...   >>>>>>>   >>>>   >>>>>   >>>>>>>>   ...
```

- `"3:jmm:agm:aam:aaf:"`

```
    - read (2 gaps):             oo---oooo-----------oo
    - transcript:     ...   >>>>>>>   >>>>   >>>>>   >>>>>>>>   ...
```

```
> U1.OV00_is_acomp <- isCompatibleWithSkippedExons(U1.ovenc)
> table(U1.OV00_is_acomp)  # 860 "almost compatible" overlaps

U1.OV00_is_acomp
 FALSE   TRUE
495625    860
```

Finally, let's extract the "almost compatible" overlaps from `U1.OV00`:

```
> U1.acompOV00 <- U1.OV00[U1.OV00_is_acomp]
```

9.1.2 Tabulate the "almost compatible" single-end overlaps

Number of "almost compatible" transcripts for each alignment in `U1.GAL`:

```
> U1.GAL_nacomptx <- nhitPerQuery(U1.acompOV00)
> mcols(U1.GAL)$nacomptx <- U1.GAL_nacomptx
> head(U1.GAL)

GappedAlignments with 6 alignments and 5 metadata columns:
                    seqnames strand       cigar    qwidth     start       end     width      ngap |
                       <Rle>  <Rle> <character> <integer> <integer> <integer> <integer> <integer> |
  SRR031729.3941844     chr4      -         75M        75       892       966        75         0 |
  SRR031728.3674563     chr4      -         75M        75       919       993        75         0 |
  SRR031729.8532600     chr4      +         75M        75       924       998        75         0 |
  SRR031729.2779333     chr4      +         75M        75       936      1010        75         0 |
  SRR031728.2826481     chr4      +         75M        75       949      1023        75         0 |
  SRR031728.2919098     chr4      -         75M        75       967      1041        75         0 |
                                                                                       seq
                                                                            <DNAStringSet>
  SRR031729.3941844 CTGTGGTGACCAACACCACAGAATGGTTCGGGCCCAATTAGAGGGTTCCCTGCCCCTTTCCTGGCTAGGTTGTCC
  SRR031728.3674563 TCGGGCCCAATTAGAGGGTTCCCTGCCCCTTTCCTGGCTAGGTTGTCCGCTAGCTCATTTCCTGGGCTGTTGTTG
  SRR031729.8532600 CCCAATTAGAGGATTCTCTGCCCCTTTCCTGGCTAGGTTGTCCGGTAGCTCATTTCCCGGGATGTTGTTGTGTCC
  SRR031729.2779333 GTTCTCTGCCCCTTTCCTGGCTAGGTTGTCCGCTAGCTCATTTCCCGAGATGTTGTTGTGTCCCGGGACCCACCT
  SRR031728.2826481 TTCCTGGCTAGGTTGTCCGCTAGCTCATTTCCCGGGCTGTTGTTGTGTCCCGGGACACACCTTATTGTGAGTTTG
  SRR031728.2919098 GCTAGCTCATTTCCCGGGAGGTTGTTGTGTCCCGGGACCCACCTTATTGTGAGTTTGTTGACAGCTCCAAGTTTG
                           NM       ntx   ncomptx  nacomptx
                    <integer> <integer> <integer> <integer>
  SRR031729.3941844         1         0         0         0
  SRR031728.3674563         3         0         0         0
  SRR031729.8532600         2         0         0         0
  SRR031729.2779333         1         0         0         0
  SRR031728.2826481         2         0         0         0
  SRR031728.2919098         1         0         0         0
  ---
  seqlengths:
      chr2L    chr2R    chr3L    chr3R     chr4     chrM     chrX  chrYHet
   23011544 21146708 24543557 27905053  1351857    19517 22422827   347038

> table(U1.GAL_nacomptx)

U1.GAL_nacomptx
     0      1      2      3      4      5      6      7      8      9
203902    299     28     79     20      1     12      6      3      5

> mean(U1.GAL_nacomptx >= 1)

[1] 0.002216731
```

Only 0.22% of the alignments in `U1.GAL` are "almost compatible" with at least 1 transcript in `exbytx`.
Number of "almost compatible" alignments for each transcript:

```
> U1.exbytx_nacompOV00 <- nhitPerSubject(U1.acompOV00)
> names(U1.exbytx_nacompOV00) <- names(exbytx)
> table(U1.exbytx_nacompOV00)

U1.exbytx_nacompOV00
    0     1     2     3     4     6     7     8     9    10    12    13    14    18    20    21
22911    43     5    14     8     2     4     4     9     2     1     1     1     2     1     2
   32    34    44    55   170
    1     1     3     1     1
```

```
> mean(U1.exbytx_nacompOV00 >= 50)
```

```
[1] 0.0000868923
```

Only 0.0087% of the transcripts in `exbytx` are "almost compatible" with at least 50 alignments in `U1.GAL`.

Finally note that the "query start in transcript" values returned by `extractQueryStartInTranscript` are also defined for "almost compatible" overlaps:

```
> head(subset(U1.OV00_qstart, U1.OV00_is_acomp))
```

```
       startInTranscript firstSpannedExonRank startInFirstSpannedExon
132461                66                    1                      66
132472                84                    1                      84
134409               757                    7                      39
134410               689                    8                      39
134411               631                    6                      39
134412               877                    7                      39
```

9.2 "Almost compatible" paired-end overlaps

9.2.1 "Almost compatible" paired-end encodings

`U3.ovenc` contains 5 unique paired-end encodings "almost compatible" with the splicing of the transcript:

```
> sort(U3.ovenc_table[isCompatibleWithSkippedExons(U3.unique_encodings)])
```

```
    2--1:jm--m:am--m:am--m:af--i:     1--2:i--jm:a--am:a--am:a--af:
                                1                                 4
2--2:jm--mm:am--mm:af--jm:aa--af:           1--2:i--jm:a--am:a--af:
                                9                                36
          2--1:jm--m:am--m:af--i:
                               54
```

Paired-end encodings `"2--1:jm--m:am--m:af--i:"` (54 occurences in `U3.ovenc`), `"1--2:i--jm:a--am:a--af:"` (36 occurences in `U3.ovenc`), and `"2--2:jm--mm:am--mm:af--jm:aa--af:"` (9 occurences in `U3.ovenc`), correspond to the following paired-end overlaps:

- `"2--1:jm--m:am--m:af--i:"`

```
- paired-end read (1 gap on the first end, no gap on the
  last end):              ooo----------o  oooo
- transcript:     ...   >>>>>   >>>>   >>>>>>>>>   ...
```

- `"1--2:i--jm:a--am:a--af:"`

```
- paired-end read (no gap on the first end, 1 gap on the
  last end):              oooo   oo---------oo
- transcript:     ...   >>>>>>>>>>>   >>>   >>>>>>   ...
```

- `"2--2:jm--mm:am--mm:af--jm:aa--af:"`

```
- paired-end read (1 gap on the first end, 1 gap on the
  last end):                o----------ooo   oo---oo
- transcript:     ...   >>>>>   >>>>   >>>>>>>>   >>>>>>   ...
```

Note: switch use of "first" and "last" above if the read was "flipped".

```
> U3.OV00_is_acomp <- isCompatibleWithSkippedExons(U3.ovenc)
> table(U3.OV00_is_acomp)  # 104 "almost compatible" paired-end overlaps
```

```
U3.OV00_is_acomp
 FALSE   TRUE
102779    104
```

Finally, let's extract the "almost compatible" paired-end overlaps from `U3.OV00`:

```
> U3.acompOV00 <- U3.OV00[U3.OV00_is_acomp]
```

9.2.2 Tabulate the "almost compatible" paired-end overlaps

Number of "almost compatible" transcripts for each alignment pair in `U3.GALP`:

```
> U3.GALP_nacomptx <- nhitPerQuery(U3.acompOV00)
> mcols(U3.GALP)$nacomptx <- U3.GALP_nacomptx
> head(U3.GALP)

GappedAlignmentPairs with 6 alignment pairs and 3 metadata columns:
                   seqnames strand :       ranges --       ranges |       ntx   ncomptx  nacomptx
                      <Rle>  <Rle> :    <IRanges> --    <IRanges> | <integer> <integer> <integer>
SRR031715.1138209      chr4      + : [ 169,  205] -- [ 326,  362] |         0         0         0
 SRR031714.756385      chr4      + : [ 943,  979] -- [1086, 1122] |         0         0         0
SRR031714.5054563      chr4      + : [ 946,  982] -- [ 986, 1022] |         0         0         0
SRR031715.1722593      chr4      + : [ 966, 1002] -- [1108, 1144] |         0         0         0
SRR031715.2202469      chr4      + : [ 966, 1002] -- [1114, 1150] |         0         0         0
SRR031714.3544437      chr4      - : [1087, 1123] -- [ 963,  999] |         0         0         0
---
seqlengths:
    chr2L    chr2R    chr3L    chr3R     chr4     chrM     chrX  chrYHet
 23011544 21146708 24543557 27905053  1351857    19517 22422827   347038

> table(U3.GALP_nacomptx)

U3.GALP_nacomptx
    0     1     3     4     6
45745    75     5     2     1

> mean(U3.GALP_nacomptx >= 1)

[1] 0.00181112
```

Only 0.18% of the alignment pairs in `U3.GALP` are "almost compatible" with at least 1 transcript in `exbytx`.

Number of "almost compatible" alignment pairs for each transcript:

```
> U3.exbytx_nacompOV00 <- nhitPerSubject(U3.acompOV00)
> names(U3.exbytx_nacompOV00) <- names(exbytx)
> table(U3.exbytx_nacompOV00)

U3.exbytx_nacompOV00
    0     1     5     8    66
22997    15     3     1     1

> mean(U3.exbytx_nacompOV00 >= 50)

[1] 0.00004344615
```

Only 0.0043% of the transcripts in `exbytx` are "almost compatible" with at least 50 alignment pairs in `U3.GALP`.

Finally note that the "query start in transcript" values returned by `extractQueryStartInTranscript` are also defined for "almost compatible" paired-end overlaps:

```
> head(subset(U3.OV00_Lqstart, U3.OV00_is_acomp))

      startInTranscript firstSpannedExonRank startInFirstSpannedExon
43359               785                    4                       6
43366               808                    4                      29
43373               819                    4                      40
43380               785                    4                       6
43387               785                    4                       6
43394               810                    4                      31

> head(subset(U3.OV00_Rqstart, U3.OV00_is_acomp))
```

```
      startInTranscript firstSpannedExonRank startInFirstSpannedExon
43359               845                    4                      66
43366               847                    4                      68
43373               849                    4                      70
43380               842                    4                      63
43387               847                    4                      68
43394               847                    4                      68
```

10 Detect novel splice junctions

10.1 By looking at single-end overlaps

An alignment in `U1.GAL` with "almost compatible" overlaps but no "compatible" overlaps suggests the presence of one or more transcripts that are not in our annotations.

First we extract the index of those alignments (*nsj* here stands for "**n**ovel **s**plice **j**unction"):

```
> U1.GAL_is_nsj <- U1.GAL_nacomptx != 0L & U1.GAL_ncomptx == 0L
> head(which(U1.GAL_is_nsj))
```

```
[1] 57972 57974 58321 67251 67266 67267
```

We make this an index into `U1.OV00`:

```
> U1.OV00_is_nsj <- queryHits(U1.OV00) %in% which(U1.GAL_is_nsj)
```

We intersect with `U1.OV00_is_acomp` and then subset `U1.OV00` to keep only the overlaps that suggest novel splicing:

```
> U1.OV00_is_nsj <- U1.OV00_is_nsj & U1.OV00_is_acomp
> U1.nsjOV00 <- U1.OV00[U1.OV00_is_nsj]
```

For each overlap in `U1.nsjOV00`, we extract the ranks of the skipped exons (we use a list for this as there might be more than 1 skipped exon per overlap):

```
> U1.nsjOV00_skippedex <- extractSkippedExonRanks(U1.ovenc)[U1.OV00_is_nsj]
> names(U1.nsjOV00_skippedex) <- queryHits(U1.nsjOV00)
> table(elementLengths(U1.nsjOV00_skippedex))
```

```
  1   2   3   5 
226  91   2   1 
```

Finally, we split `U1.nsjOV00_skippedex` by transcript names:

```
> f <- factor(names(exbytx)[subjectHits(U1.nsjOV00)], levels=names(exbytx))
> U1.exbytx_skippedex <- split(U1.nsjOV00_skippedex, f)
```

`U1.exbytx_skippedex` is a named list of named lists of integer vectors. The first level of names (outer names) are transcript names and the second level of names (inner names) are alignment indices into `U1.GAL`:

```
> head(names(U1.exbytx_skippedex))  # transcript names
```

```
[1] "FBtr0300689" "FBtr0300690" "FBtr0078100" "FBtr0078101" "FBtr0302164" "FBtr0301733"
```

Transcript FBtr0089124 receives 7 hits. All of them skip exons 9 and 10:

```
> U1.exbytx_skippedex$FBtr0089124
```

```
$`104549`
[1]  9 10

$`104550`
[1]  9 10
```

```
$`104553`
[1]  9 10

$`104557`
[1]  9 10

$`104560`
[1]  9 10

$`104572`
[1]  9 10

$`104577`
[1]  9 10
```

Transcript FBtr0089147 receives 4 hits. Two of them skip exon 2, one of them skips exons 2 to 6, and one of them skips exon 10:

```
> U1.exbytx_skippedex$FBtr0089147

$`72828`
[1] 10

$`74018`
[1] 2 3 4 5 6

$`74664`
[1] 2

$`74670`
[1] 2
```

A few words about the interpretation of `U1.exbytx_skippedex`: Because of how we've conducted this analysis, the aligments reported in `U1.exbytx_skippedex` are guaranteed to not have any "compatible" overlaps with other known transcripts. All we can say, for example in the case of transcript FBtr0089124, is that the 7 reported hits that skip exons 9 and 10 show evidence of one or more unknown transcripts with a splice junction that corresponds to the gap between exons 8 and 11. But without further analysis, we can't make any assumption about the exons structure of those unknown transcripts. In particular, we cannot assume the existence of an unknown transcript made of the same exons as transcript FBtr0089124 minus exons 9 and 10!

10.2 By looking at paired-end overlaps

[COMING SOON...]

11 sessionInfo()

```
> sessionInfo()

R version 3.0.0 (2013-04-03)
Platform: x86_64-unknown-linux-gnu (64-bit)

locale:
 [1] LC_CTYPE=en_US.UTF-8       LC_NUMERIC=C               LC_TIME=en_US.UTF-8
 [4] LC_COLLATE=C               LC_MONETARY=en_US.UTF-8    LC_MESSAGES=en_US.UTF-8
 [7] LC_PAPER=C                 LC_NAME=C                  LC_ADDRESS=C
[10] LC_TELEPHONE=C             LC_MEASUREMENT=en_US.UTF-8 LC_IDENTIFICATION=C

attached base packages:
[1] parallel  stats     graphics  grDevices utils     datasets  methods   base
```

```
other attached packages:
 [1] TxDb.Dmelanogaster.UCSC.dm3.ensGene_2.9.0 BSgenome.Dmelanogaster.UCSC.dm3_1.3.19
 [3] pasillaBamSubset_0.0.7                    BSgenome.Scerevisiae.UCSC.sacCer2_1.3.19
 [5] org.Sc.sgd.db_2.9.0                       RSQLite_0.11.3
 [7] DBI_0.2-5                                 GenomicFeatures_1.12.0
 [9] AnnotationDbi_1.22.1                      leeBamViews_0.99.22
[11] BSgenome_1.28.0                           Biobase_2.20.0
[13] EatonEtAlChIPseq_0.0.9                    rtracklayer_1.20.1
[15] ShortRead_1.18.0                          latticeExtra_0.6-24
[17] RColorBrewer_1.0-5                        lattice_0.20-15
[19] Rsamtools_1.12.1                          Biostrings_2.28.0
[21] GenomicRanges_1.12.2                      IRanges_1.18.0
[23] BiocGenerics_0.6.0

loaded via a namespace (and not attached):
[1] RCurl_1.95-4.1 XML_3.96-1.1   biomaRt_2.16.0 bitops_1.0-5   grid_3.0.0     hwriter_1.3
[7] stats4_3.0.0   tools_3.0.0    zlibbioc_1.6.0
```
