# Supplementary material for: Software for Computing and Annotating Genomic Ranges
Source: PLoS Comput Biol. 2013 Aug 8;9(8):e1003118. doi: 10.1371/journal.pcbi.1003118 (PMC3738458; doi:10.1371/journal.pcbi.1003118)
Supplement: Software S2 — The GenomicRanges package. The GenomicRanges package defines general purpose containers for storing genomic ranges as well as more specialized containers for storing alignments against a reference genome. (GZ) [file pcbi.1003118.s002.gz › GenomicRanges/inst/doc/summarizeOverlaps-modes.pdf]

	Union	IntersectionStrict	IntersectionNotEmpty
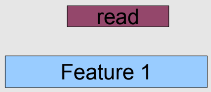	Feature I	Feature I	Feature I
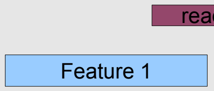	Feature I	No hit	Feature I
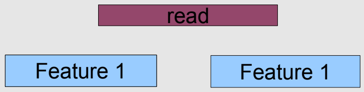	Feature I	No hit	Feature I
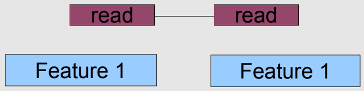	Feature I	Feature I	Feature I
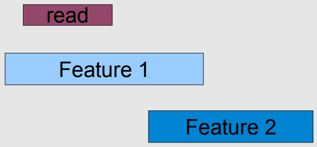	Feature I	Feature I	Feature I
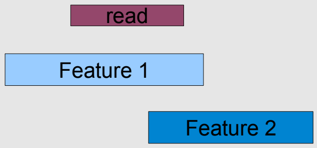	No hit	Feature 1	Feature I
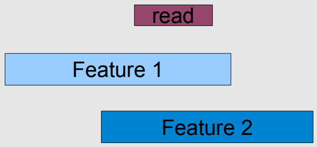	No hit	No hit	No hit

* Picture reproduced from HTSeq web site :
http://www-huber.embl.de/users/anders/HTSeq/doc/count.html
