# Supplementary material for: Software for Computing and Annotating Genomic Ranges
Source: PLoS Comput Biol. 2013 Aug 8;9(8):e1003118. doi: 10.1371/journal.pcbi.1003118 (PMC3738458; doi:10.1371/journal.pcbi.1003118)
Supplement: Software S2 — The GenomicRanges package. The GenomicRanges package defines general purpose containers for storing genomic ranges as well as more specialized containers for storing alignments against a reference genome. (GZ) [file pcbi.1003118.s002.gz › GenomicRanges/inst/doc/summarizeOverlaps.pdf]

Counting with `summarizeOverlaps`

Valerie Obenchain

Edited: August 2012; Compiled: April 16, 2013

Contents

1 Introduction **1**

2 A First Example **1**

3 Counting Modes **2**

4 Counting Features **3**

5 `pasilla` Data **6**
5.1 source files 6
5.2 counting 7

6 Refererences **8**

1 Introduction

This vignette illustrates how reads mapped to a genome can be counted with `summarizeOverlaps`. Different "modes" of counting are provided to resolve reads that overlap multiple features. The built-in count modes are fashioned after the "Union", "IntersectionStrict", and "IntersectionNotEmpty" methods found in the HTSeq package by Simon Anders (see references).

2 A First Example

In this example reads are counted from a list of BAM files and returned in a `matrix` for use in further analysis such as those offered in DESeq and edgeR.

```
> library(Rsamtools)
> library(DESeq)
> library(edgeR)
> fls <- list.files(system.file("extdata",package="GenomicRanges"),
+     recursive=TRUE, pattern="*bam$", full=TRUE)
```

```
> bfl <- BamFileList(fls, index=character())
> features <- GRanges(
+     seqnames = c(rep("chr2L", 4), rep("chr2R", 5), rep("chr3L", 2)),
+     ranges = IRanges(c(1000, 3000, 4000, 7000, 2000, 3000, 3600, 4000,
+         7500, 5000, 5400), width=c(rep(500, 3), 600, 900, 500, 300, 900,
+         300, 500, 500)), "-",
+     group_id=c(rep("A", 4), rep("B", 5), rep("C", 2)))
> olap <- summarizeOverlaps(features, bfl)
> deseq <- newCountDataSet(assays(olap)$counts, rownames(colData(olap)))
> edger <- DGEList(assays(olap)$counts, group=rownames(colData(olap)))
```

3 Counting Modes

The modes of "Union", "IntersectionStrict" and "IntersectionNotEmpty" provide different approaches to resolving reads that overlap multiple features. Figure 1 illustrates how both simple and gapped reads are handled by the modes. Note that a read is counted a maximum of once; there is no double counting. These methods do not currently handle paired-end reads. For additional detail on the counting modes see the `summarizeOverlaps` man page.

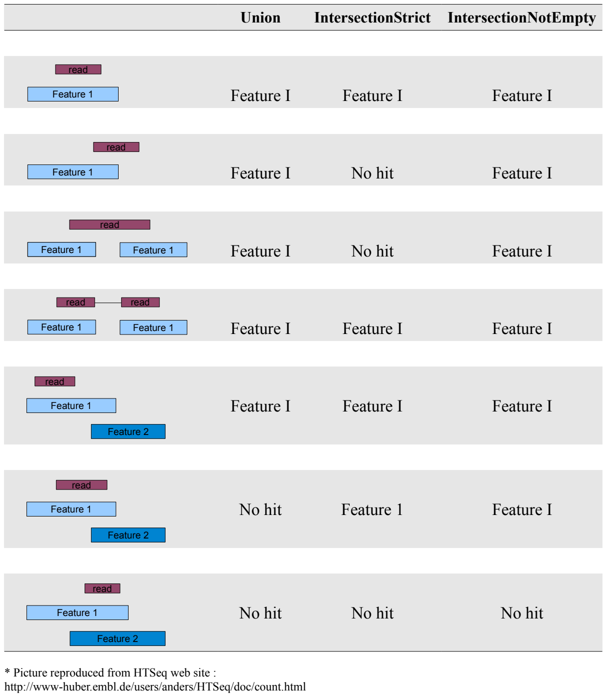

Figure 1: Counting Modes

4 Counting Features

Features can be exons, transcripts, genes or any region of interest. The number of ranges that define a single feature is specified in the `features` argument.

When annotation regions of interest are defined by a single range a *GRanges* should be used as the `features` argument. With a *GRanges* it is assumed that each row (i.e., each range) represents a distinct feature. If `features` was a *GRanges* of exons, the result would be counts per exon.

When the region of interest is defined by one or more ranges the `features` argument should be a *GRangesList*. In practice this could be a list of exons by gene or transcripts by gene or other similar relationships. The count result will

be the same length as the *GRangesList*. For a list of exons by genes, the result would be counts per gene.

The combination of defining the features as either*GRanges* or *GRangesList* and choosing a counting mode controls how `summarizeOverlaps` assigns hits. Reguardless of the mode chosen, each read is assigned to at most a single feature. These options are intended to provide flexibility in defining different biological problems.

This next example demonstrates how the same read can be counted differently depending on how the `features` argument is specified. We use a single read that overlaps two ranges, gr1 and gr2.

```
> rd <- GappedAlignments("a", seqnames = Rle("chr1"), pos = as.integer(100),
+     cigar = "300M", strand = strand("+"))
> gr1 <- GRanges("chr1", IRanges(start=50, width=150), strand="+")
> gr2 <- GRanges("chr1", IRanges(start=350, width=150), strand="+")
```

When provided as a *GRanges* both gr1 and gr2 are considered distinct features. In this case none of the modes count the read as a hit. Mode `Union` discards the read becasue more than 1 feature is overlapped. `IntersectionStrict` requires the read to fall completely within a feature which is not the case for either gr1 or gr2. `IntersetctionNotEmpty` requires the read to overlap a single unique disjoint region of the `features`. In this case gr1 and gr2 do not overlap so each range is considered a unique disjoint region. However, the read overlaps both gr1 and gr2 so a decision cannot be made and the read is discarded.

```
> gr <- c(gr1, gr2)
> data.frame(union = assays(summarizeOverlaps(gr, rd))$counts,
+            intStrict = assays(summarizeOverlaps(gr, rd,
+                mode="IntersectionStrict"))$counts,
+            intNotEmpty = assays(summarizeOverlaps(gr, rd,
+                mode="IntersectionNotEmpty"))$counts)

  union intStrict intNotEmpty
1     0         0           0
2     0         0           0
```

Next we count with `features` as a *GRangesList*; this is list of length 1 with 2 elements. Modes `Union` and `IntersectionNotEmpty` both count the read for the single feature.

```
> grl <- GRangesList(c(gr1, gr2))
> data.frame(union = assays(summarizeOverlaps(grl, rd))$counts,
+            intStrict = assays(summarizeOverlaps(grl, rd,
+                mode="IntersectionStrict"))$counts,
+            intNotEmpty = assays(summarizeOverlaps(grl, rd,
+                mode="IntersectionNotEmpty"))$counts)

  union intStrict intNotEmpty
1     1         0           1
```

In this more complicated example we have 7 reads, 5 are simple and 2 have gaps in the CIGAR. There are 12 ranges that will serve as the `features`.

```
> group_id <- c("A", "B", "C", "C", "D", "D", "E", "F", "G", "G", "H", "H")
> features <- GRanges(
+     seqnames = Rle(c("chr1", "chr2", "chr1", "chr1", "chr2", "chr2",
+         "chr1", "chr1", "chr2", "chr2", "chr1", "chr1")),
+     strand = strand(rep("+", length(group_id))),
+     ranges = IRanges(
+         start=c(1000, 2000, 3000, 3600, 7000, 7500, 4000, 4000, 3000, 3350, 5000, 5400),
+         width=c(500, 900, 500, 300, 600, 300, 500, 900, 150, 200, 500, 500)),
+    DataFrame(group_id)
+ )
> reads <- GappedAlignments(
+     names = c("a","b","c","d","e","f","g"),
+     seqnames = Rle(c(rep(c("chr1", "chr2"), 3), "chr1")),
+     pos = as.integer(c(1400, 2700, 3400, 7100, 4000, 3100, 5200)),
+     cigar = c("500M", "100M", "300M", "500M", "300M", "50M200N50M", "50M150N50M"),
+     strand = strand(rep.int("+", 7L)))
>
```

Using a *GRanges* as the `features` all 12 ranges are considered to be different features and counts are produced for each row,

```
> data.frame(union = assays(summarizeOverlaps(features, reads))$counts,
+            intStrict = assays(summarizeOverlaps(features, reads,
+                mode="IntersectionStrict"))$counts,
+            intNotEmpty = assays(summarizeOverlaps(features, reads,
+                mode="IntersectionNotEmpty"))$counts)

   union intStrict intNotEmpty
1      1         0           1
2      1         1           1
3      0         0           0
4      0         0           0
5      0         1           1
6      0         0           0
7      0         0           0
8      0         0           0
9      0         0           0
10     0         0           0
11     0         1           1
12     0         0           0
```

When the data are split by group to create a *GRangesList* the highest list-levels are treated as different features and the multiple list elements are considered part of the same features. Counts are returned for each group.

```
> lst <- split(features, mcols(features)[["group_id"]])
> length(lst)

[1] 8

> data.frame(union = assays(summarizeOverlaps(lst, reads))$counts,
+            intStrict = assays(summarizeOverlaps(lst, reads,
+                mode="IntersectionStrict"))$counts,
+            intNotEmpty = assays(summarizeOverlaps(lst, reads,
+                mode="IntersectionNotEmpty"))$counts)

  union intStrict intNotEmpty
A     1         0           1
B     1         1           1
C     1         0           1
D     1         1           1
E     0         0           0
F     0         0           0
G     1         1           1
H     1         1           1
```

If desired, users can supply their own counting function as the `mode` argument and take advantage of the infrastructure for counting over multiple BAM files and parsing the results into a *SummarizedExperiment*. See `?'BamViews-class'` or `?'BamFile-class'` in Rsamtools.

5 pasilla Data

In this excercise we count the pasilla data by gene and by transcript then create a *CountDataSet*. This object can be used in differential expression methods offered in the DESeq package.

5.1 source files

Files are available through NCBI Gene Expression Omnibus (GEO), accession number GSE18508. `http://www.ncbi.nlm.nih.gov/projects/geo/query/acc.cgi?acc=GSE18508`. SAM files can be converted to BAM with the `asBam` function in the Rsamtools package. Of the seven files available, 3 are single-reads and 4 are paired-end. Smaller versions of untreated1 (single-end) and untreated2 (paired-end) have been made available in the pasillaBamSubset package. This subset includes chromosome 4 only.

`summarizeOverlaps` is capable of counting paired-end reads in both a `BamFile`-method (set argument `singleEnd=TRUE`) or a `GappedAlignmentPairs`-method. For this example, we use the 3 single-end read files,

- treated1.bam

- untreated1.bam
- untreated2.bam

Annotations are retrieved as a GTF file from the ENSEMBL web site. We download the file our local disk, then use Rtracklayer's `import` function to parse the file to a *GRanges* instance.

```
> library(rtracklayer)
> fl <- paste0("ftp://ftp.ensembl.org/pub/release-62/",
+              "gtf/drosophila_melanogaster/",
+              "Drosophila_melanogaster.BDGP5.25.62.gtf.gz")
> gffFile <- file.path(tempdir(), basename(fl))
> download.file(fl, gffFile)
> gff0 <- import(gffFile, asRangedData=FALSE)
```

Subset on the protein-coding, exon regions of chromosome 4 and split by gene id.

```
> idx <- mcols(gff0)$source == "protein_coding" &
+            mcols(gff0)$type == "exon" &
+            seqnames(gff0) == "4"
> gff <- gff0[idx]
> ## adjust seqnames to match Bam files
> seqlevels(gff) <- paste("chr", seqlevels(gff), sep="")
> chr4genes <- split(gff, mcols(gff)$gene_id)
```

5.2 counting

The `params` argument can be used to subset the reads in the bam file on characteristics such as position, unmapped or paired-end reads. Quality scores or the "NH" tag, which identifies reads with multiple mappings, can be included as metadata columns for further subsetting. See `?ScanBamParam` for details about specifying the `param` argument.

```
> param <- ScanBamParam(
+              what='qual',
+              which=GRanges("chr4", IRanges(1, 1e6)),
+              flag=scanBamFlag(isUnmappedQuery=FALSE, isPaired=NA),
+              tag="NH")
```

We use `summarizeOverlaps` to count with the default mode of "Union". If a `param` argument is not included all reads from the BAM file are counted.

```
> fls <- c("treated1.bam", "untreated1.bam", "untreated2.bam")
> path <- "pathToBAMFiles"
> bamlst <- BamFileList(fls, index=character())
> genehits <- summarizeOverlaps(chr4genes, bamlst, mode="Union")
```

A `CountDataSet` is constructed from the counts and experiment data in *pasilla*.

```
> expdata = new("MIAME",
+               name="pasilla knockdown",
+               lab="Genetics and Developmental Biology, University of
+                   Connecticut Health Center",
+               contact="Dr. Brenton Graveley",
+               title="modENCODE Drosophila pasilla RNA Binding Protein RNAi
+                   knockdown RNA-Seq Studies",
+               url="http://www.ncbi.nlm.nih.gov/projects/geo/query/acc.cgi?acc=GSE18508",
+               abstract="RNA-seq of 3 biological replicates of from the Drosophila
+                   melanogaster S2-DRSC cells that have been RNAi depleted of mRNAs
+                   encoding pasilla, a mRNA binding protein and 4 biological replicates
+                   of the the untreated cell line.")
>               pubMedIds(expdata) <- "20921232"
> design <- data.frame(
+               condition=c("treated", "untreated", "untreated"),
+               replicate=c(1,1,2),
+               type=rep("single-read", 3),
+               countfiles=path(colData(genehits)[,1]), stringsAsFactors=TRUE)
> geneCDS <- newCountDataSet(
+                   countData=assays(genehits)$counts,
+                   conditions=design)
> experimentData(geneCDS) <- expdata
> sampleNames(geneCDS) = colnames(genehits)
```

If the primary interest is to count by transcript instead of by gene, the annotation file can be split on transcript id.

```
> chr4tx <- split(gff, mcols(gff)$transcript_id)
> txhits <- summarizeOverlaps(chr4tx, bamlst)
> txCDS <- newCountDataSet(assays(txhits)$counts, design)
> experimentData(txCDS) <- expdata
```

6 Refererences

http://www-huber.embl.de/users/anders/HTSeq/doc/overview.html http://www-huber.embl.de/users/anders/HTSeq/doc/count.html
