# Supplementary material for: Software for Computing and Annotating Genomic Ranges
Source: PLoS Comput Biol. 2013 Aug 8;9(8):e1003118. doi: 10.1371/journal.pcbi.1003118 (PMC3738458; doi:10.1371/journal.pcbi.1003118)
Supplement: Software S3 — The GenomicFeatures package. The GenomicFeatures package is a set of tools and methods for making and manipulating transcript-centric annotations. (GZ) [file pcbi.1003118.s003.gz › GenomicFeatures/inst/doc/GenomicFeatures.pdf]

Making and Utilizing TranscriptDb Objects

Marc Carlson Patrick Aboyoun Hervé Pagès
Seth Falcon Martin Morgan

April 25, 2013

1 Introduction

The *GenomicFeatures* package retrieves and manages transcript-related features from the UCSC Genome Bioinformatics[1] and BioMart[2] data resources. The package is useful for ChIP-chip, ChIP-seq, and RNA-seq analyses.

```
> library("GenomicFeatures")
```

2 *TranscriptDb* Objects

The *GenomicFeatures* package uses *TranscriptDb* objects to store transcript metadata. This class maps the 5' and 3' untranslated regions (UTRs), protein coding sequences (CDSs) and exons for a set of mRNA transcripts to their associated genome. *TranscriptDb* objects have numerous accessors functions to allow such features to be retrieved individually or grouped together in a way that reflects the underlying biology.

All *TranscriptDb* objects are backed by a SQLite database that manages genomic locations and the relationships between pre-processed mRNA transcripts, exons, protein coding sequences, and their related gene identifiers.

3 Retrieving Data from *TranscriptDb* objects

3.1 Loading Transcript Data

There are two ways that users can load pre-existing data to generate a *TranscriptDb* object. One method is to use the `loadDb` method to load the object directly from an appropriate .sqlite database file.

[1]`http://genome.ucsc.edu/`
[2]`http://www.biomart.org/`

Here we are loading a previously created *TranscriptDb* object based on UCSC known gene data. This database only contains a small subset of the possible annotations for human and is only included to demonstrate and test the functionality of the *GenomicFeatures* packageas a demonstration.

```
> samplefile <- system.file("extdata", "UCSC_knownGene_sample.sqlite",
+                           package="GenomicFeatures")
> txdb <- loadDb(samplefile)
> txdb

TranscriptDb object:
| Db type: TranscriptDb
| Supporting package: GenomicFeatures
| Data source: UCSC
| Genome: hg18
| Genus and Species: Homo sapiens
| UCSC Table: knownGene
| Resource URL: http://genome.ucsc.edu/
| Type of Gene ID: Entrez Gene ID
| Full dataset: no
| miRBase build ID: NA
| transcript_nrow: 135
| exon_nrow: 544
| cds_nrow: 324
| Db created by: GenomicFeatures package from Bioconductor
| Creation time: 2012-04-13 14:47:54 -0700 (Fri, 13 Apr 2012)
| GenomicFeatures version at creation time: 1.9.4
| RSQLite version at creation time: 0.11.1
| DBSCHEMAVERSION: 1.0
```

In this case, the *TranscriptDb* object has been returned by the `loadDb` method.

More commonly however, we expect that users will just load a TranscriptDb annotation package like this:

```
> library(TxDb.Hsapiens.UCSC.hg19.knownGene)
> txdb <- TxDb.Hsapiens.UCSC.hg19.knownGene #shorthand (for convenience)
> txdb

TranscriptDb object:
| Db type: TranscriptDb
```

```
| Supporting package: GenomicFeatures
| Data source: UCSC
| Genome: hg19
| Organism: Homo sapiens
| UCSC Table: knownGene
| Resource URL: http://genome.ucsc.edu/
| Type of Gene ID: Entrez Gene ID
| Full dataset: yes
| miRBase build ID: GRCh37
| transcript_nrow: 80922
| exon_nrow: 286852
| cds_nrow: 235842
| Db created by: GenomicFeatures package from Bioconductor
| Creation time: 2013-03-08 09:43:09 -0800 (Fri, 08 Mar 2013)
| GenomicFeatures version at creation time: 1.11.14
| RSQLite version at creation time: 0.11.2
| DBSCHEMAVERSION: 1.0
```

Loading the package like this will also create a *TranscriptDb* object, and by default that object will have the same name as the package itself.

3.2 Pre-filtering data based on Chromosomes

It is possible to filter the data that is returned from a *TranscriptDb* object based on it's chromosome. This can be a useful way to limit the things that are returned if you are only interested in studying a handful of chromosomes.

To determine which chromosomes are currently active, use the `isActiveSeq` method. For example:

```
> head(isActiveSeq(txdb))

chr1 chr2 chr3 chr4 chr5 chr6
TRUE TRUE TRUE TRUE TRUE TRUE
```

Will tell you all the chromosomes that are active for the TxDb.Hsapiens.UCSC.hg19.knownGene *TranscriptDb* object (by default it will be all of them).

If you then wanted to only set Chromosome 1 to be active you could do it like this:

```
> isActiveSeq(txdb)[seqlevels(txdb)] <- FALSE
> isActiveSeq(txdb) <- c("chr1"=TRUE)
```

So if you ran this, then from this point on in your R session only chromosome 1 would be consulted when you call the various retrieval methods..

Exercise 1
Use `isActiveSeq` *to set only chromsome 15 to be active. BTW, the rest of this vignette will assume you have succeeded at this.*

Solution:

```
> isActiveSeq(txdb)[seqlevels(txdb)] <- FALSE
> isActiveSeq(txdb) <- c("chr15"=TRUE)
```

3.3 Retrieving data using the select method

The *TranscriptDb* objects inherit from *AnnotationDb* objects (just as the *ChipDb* and *OrgDb* objects do). One of the implications of this relationship is that these object ought to be used in similar ways to each other. Therefore we have written supporting `cols`, `keytypes`, `keys` and `select` methods for *TranscriptDb* objects.

These methods can be a useful way of extracting data from a *TranscriptDb* object. And they are used in the same way that they would be used to extract information about a *ChipDb* or an *OrgDb* object. Here is a simple example of how to find the UCSC transcript names that match with a set of gene IDs.

```
> keys <- c("100033416", "100033417", "100033420")
> cols(txdb)

 [1] "CDSID"      "CDSNAME"    "CDSCHROM"   "CDSSTRAND"  "CDSSTART"
 [6] "CDSEND"     "EXONID"     "EXONNAME"   "EXONCHROM"  "EXONSTRAND"
[11] "EXONSTART"  "EXONEND"    "GENEID"     "TXID"       "EXONRANK"
[16] "TXNAME"     "TXCHROM"    "TXSTRAND"   "TXSTART"    "TXEND"

> keytypes(txdb)

[1] "GENEID"   "TXID"     "TXNAME"   "EXONID"   "EXONNAME" "CDSID"    "CDSNAME"

> select(txdb, keys = keys, cols="TXNAME", keytype="GENEID")
```

```
     GENEID     TXNAME
1 100033416 uc001yxl.4
2 100033417 uc001yxo.3
3 100033417 uc001yxq.3
4 100033420 uc001yxr.3
```

Exercise 2
For the genes in the example above, find the chromosome and strand information that will go with each of the transcript names.

Solution:

```
> cols(txdb)

 [1] "CDSID"      "CDSNAME"    "CDSCHROM"   "CDSSTRAND"  "CDSSTART"
 [6] "CDSEND"     "EXONID"     "EXONNAME"   "EXONCHROM"  "EXONSTRAND"
[11] "EXONSTART"  "EXONEND"    "GENEID"     "TXID"       "EXONRANK"
[16] "TXNAME"     "TXCHROM"    "TXSTRAND"   "TXSTART"    "TXEND"

> cols <- c("TXNAME", "TXSTRAND", "TXCHROM")
> select(txdb, keys=keys, cols=cols, keytype="GENEID")

     GENEID     TXNAME TXCHROM TXSTRAND
1 100033416 uc001yxl.4   chr15        +
2 100033417 uc001yxo.3   chr15        +
3 100033417 uc001yxq.3   chr15        +
4 100033420 uc001yxr.3   chr15        +
```

3.4 Methods for returning *GRanges* objects

Retrieving data with select is useful, but sometimes it is more convenient to extract the result as *GRanges* objects. This is often the case when you are doing counting or specialized overlap operations downstream. For these use cases there is another family of methods available.

Perhaps the most common operations for a *TranscriptDb* object is to retrieve the genomic coordinates or *ranges* for exons, transcripts or coding sequences. The functions `transcripts`, `exons`, and `cds` return the coordinate information as a *GRanges* object.

As an example, all transcripts present in a *TranscriptDb* object can be obtained as follows:

```
> GR <- transcripts(txdb)
> GR[1:3]

GRanges with 3 ranges and 2 metadata columns:
      seqnames               ranges strand |     tx_id     tx_name
         <Rle>            <IRanges>  <Rle> | <integer> <character>
  [1]    chr15 [20362688, 20364420]      + |     52237  uc001yte.1
  [2]    chr15 [20487997, 20496811]      + |     52238  uc001ytf.1
  [3]    chr15 [20723929, 20727150]      + |     52239  uc001ytj.3
  ---
  seqlengths:
       chr15
   102531392
```

The transcripts function returns a *GRanges* class object. You can learn a lot more about the manipulation of these objects by reading the *GenomicRanges* introductory vignette. The show method for a *GRanges* object will display the ranges, seqnames (a chromosome or a contig), and strand on the left side and then present related metadata on the right side. At the bottom, the seqlengths display all the possible seqnames along with the length of each sequence.

In addition, the transcripts function can also be used to retrieve a subset of the transcripts available such as those on the +-strand of chromosome 1.

```
> GR <- transcripts(txdb, vals <- list(tx_chrom = "chr15", tx_strand = "+"))
> length(GR)

[1] 1718

> unique(strand(GR))

[1] +
Levels: + - *
```

The exons and cds functions can also be used in a similar fashion to retrive genomic coordinates for exons and coding sequences.

Exercise 3
Use exons to retrieve all the exons from chromosome 15. How does the length of this compare to the value returned by transcripts?

Solution:

```
> EX <- exons(txdb)
> EX[1:4]

GRanges with 4 ranges and 1 metadata column:
      seqnames               ranges strand |   exon_id
         <Rle>            <IRanges>  <Rle> | <integer>
  [1]    chr15 [20362688, 20362858]      + |    190706
  [2]    chr15 [20362943, 20363123]      + |    190707
  [3]    chr15 [20364397, 20364420]      + |    190708
  [4]    chr15 [20487997, 20488227]      + |    190709
  ---
  seqlengths:
       chr15
   102531392

> length(EX)

[1] 10658

> length(GR)

[1] 1718
```

3.5 Working with Grouped Features

Often one is interested in how particular genomic features relate to each other, and not just their location. For example, it might be of interest to group transcripts by gene or to group exons by transcript. Such groupings are supported by the `transcriptsBy`, `exonsBy`, and `cdsBy` functions.

The following call can be used to group transcripts by genes:

```
> GRList <- transcriptsBy(txdb, by = "gene")
> length(GRList)

[1] 782

> names(GRList)[10:13]

[1] "100033424" "100033425" "100033426" "100033427"
```

```
> GRList[11:12]

GRangesList of length 2:
$100033425
GRanges with 1 range and 2 metadata columns:
      seqnames               ranges strand |     tx_id     tx_name
         <Rle>            <IRanges>  <Rle> | <integer> <character>
  [1]    chr15 [25324204, 25324297]      + |     52324  uc001yxw.3

$100033426
GRanges with 1 range and 2 metadata columns:
      seqnames               ranges strand | tx_id    tx_name
  [1]    chr15 [25325288, 25325381]      + | 52325 uc001yxx.3

---
seqlengths:
     chr15
 102531392
```

The `transcriptsBy` function returns a *GRangesList* class object. As with *GRanges* objects, you can learn more about these objects by reading the *GenomicRanges* introductory vignette. The `show` method for a *GRangesList* object will display as a list of *GRanges* objects. And, at the bottom the seqlengths will be displayed once for the entire list.

For each of these three functions, there is a limited set of options that can be passed into the `by` argument to allow grouping. For the `transcriptsBy` function, you can group by gene, exon or cds, whereas for the `exonsBy` and `cdsBy` functions can only be grouped by transcript (tx) or gene.

So as a further example, to extract all the exons for each transcript you can call:

```
> GRList <- exonsBy(txdb, by = "tx")
> length(GRList)

[1] 3281

> names(GRList)[10:13]

[1] "52246" "52247" "52248" "52249"

> GRList[[12]]
```

```
GRanges with 1 range and 3 metadata columns:
      seqnames               ranges strand |   exon_id   exon_name exon_rank
         <Rle>            <IRanges>  <Rle> | <integer> <character> <integer>
  [1]    chr15 [22043463, 22043502]      + |    190748        <NA>         1
  ---
  seqlengths:
       chr15
   102531392
```

As you can see, the *GRangesList* objects returned from each function contain locations and identifiers grouped into a list like object according to the type of feature specified in the `by` argument. The object returned can then be used by functions like `findOverlaps` to contextualize alignments from high-throughput sequencing.

The identifiers used to label the *GRanges* objects depend upon the data source used to create the *TranscriptDb* object. So the list identifiers will not always be Entrez Gene IDs, as they were in the first example. Furthermore, some data sources do not provide a unique identifier for all features. In this situation, the group label will be a synthetic ID created by *GenomicFeatures* to keep the relations between features consistent in the database this was the case in the 2nd example. Even though the results will sometimes have to come back to you as synthetic IDs, you can still always retrieve the original IDs.

Exercise 4
Starting with the tx_ids that are the names of the GRList object we just made, use `select` *to retrieve that matching transcript names. Remember that the list used a* `by` *argument = "tx", so the list is grouped by transcript IDs.*

Solution:

```
> GRList <- exonsBy(txdb, by = "tx")
> tx_ids <- names(GRList)
> head(select(txdb, keys=tx_ids, cols="TXNAME", keytype="TXID"))
```

```
   TXID     TXNAME
1 52237 uc001yte.1
2 52238 uc001ytf.1
3 52239 uc001ytj.3
4 52240 uc021sex.1
```

```
5 52241 uc010tzb.1
6 52242 uc021sey.1
```

Finally, the order of the results in a *GRangesList* object can vary with the way in which things were grouped. In most cases the grouped elements of the *GRangesList* object will be listed in the order that they occurred along the chromosome. However, when exons or CDS are grouped by transcript, they will instead be grouped according to their position along the transcript itself. This is important because alternative splicing can mean that the order along the transcript can be different from that along the chromosome.

3.6 Predefined grouping functions

The `intronsByTranscript`, `fiveUTRsByTranscript` and `threeUTRsByTranscript` are convenience functions that provide behavior equivalent to the grouping functions, but in prespecified form. These functions return a *GRangesList* object grouped by transcript for introns, 5' UTR's, and 3' UTR's, respectively. Below are examples of how you can call these methods.

```
> length(intronsByTranscript(txdb))
```

```
[1] 3281
```

```
> length(fiveUTRsByTranscript(txdb))
```

```
[1] 1770
```

```
> length(threeUTRsByTranscript(txdb))
```

```
[1] 1748
```

3.7 Getting the actual sequence data

The *GenomicFeatures* package also provides provides functions for converting from ranges to actual sequence (when paired with an appropriate *BSgenome* package).

```
> library(BSgenome.Hsapiens.UCSC.hg19)
> tx_seqs1 <- extractTranscriptsFromGenome(Hsapiens,
+                                           TxDb.Hsapiens.UCSC.hg19.knownGene)
```

And, once these sequences have been extracted, you can translate them into proteins with `translate`:

```
> translate(tx_seqs1)

  A AAStringSet instance of length 3281
       width seq
   [1]   125 EDQDDEARVQYEGFRPGMYVRVEIENVPCEFV...EDHNGRQRLLKYTPQHMHCGAAFWA*FSDSCH
   [2]   288 RIAS*GRAEFSSAQTSEIQRRRSSVLLSTDPG...TFFHSVIFLFFESVFYSVYFNYGNNCFFTVTD
   [3]   588 RSGQRLPEQPEAEGGDPGKQRRRAEHRAVGLP...QEERNSKVICERDLLENETHLYLCSIKICFSS
   [4]    10 HHLNCRPQTG
   [5]     9 STVTLPHSQ
   ...   ... ...
[3277]    10 QVPMRVQVGQ
[3278]   306 MVTEFIFLGLSDSQELQTFLFMLFFVFYGGIV...LNPIIYTLRNKDMKTAIRRLRKWDAHSSVKF*
[3279]   550 LAVSLFFDLFFLFMCICCLLAQTSRVLSTGPL...LGTNRGRRQSLTPRRLHPAQLEILY*KHTVGF
[3280]   496 LAVSLFFDLFFLFMCICCLLAQTSRVLSTGPL...RDQQGQEAVTDPETFASCTARDPLLKAHCWFL
[3281]   531 LAVSLFFDLFFLFMCICCLLAQTSRVLSTGPL...LGTNRGRRQSLTPRRLHPAQLEILY*KHTVGF
```

Exercise 5
But of course this is not a meaningful translation, because the call to `extractTranscriptsFromGenome` *will have extracted all the transcribed regions of the genome regardless of whether or not they are translated. look at the manual page for* `extractTranscriptsFromGenome` *and see how you can use cdsBy to only translate only the coding regions.*

Solution:

```
> cds_seqs <- extractTranscriptsFromGenome(Hsapiens, cdsBy(txdb, by="tx"))
> translate(cds_seqs)

  A AAStringSet instance of length 1819
       width seq
   [1]   102 MYVRVEIENVPCEFVQNIDPHYPIILGGLGNS...IPLYYIEDHNGRQRLLKYTPQHMHCGAAFWA*
   [2]   435 MEWKLEQSMREQALLKAQLTQLKESLKEVQLE...PIVQDHQEHPGLGSNCCVPFFCWAWPPRRRR*
   [3]   317 MKIANNTVVTEFILLGLTQSQDIQLLVFVLIL...IYTLRNQEVKTSMKRLLSRHVVCQVDFIIRN*
   [4]   314 METANYTKVTEFVLTGLSQTPEVQLVLFVIFL...LRNPIIYTLRNKEVKAAMRKLVTKYILCKEK*
   [5]   317 MKIANNTVVTEFILLGLTQSQDIQLLVFVLIL...IYTLRNQEVKTSMKRLLSRHVVCQVDFIIRN*
   ...   ... ...
[1815]   186 MAGGVLPLRGLRALCRVLLFLSQFCILSGGEQ...PANCTVRDHVHCLGRSEFKDICQQNVFLQVY*
[1816]   258 MYNSKLWEASGHWQHYSENMFTFEIEKDTFAL...ILSENYGGKWYPVNFLKKDLWLTLTWITVVH*
[1817]   803 MAAEALAAEAVASRLERQEEDIRWLWSEVERL...NKIHGEILVTSAIDKLKNLRKTRTLNAEEAF*
```

```
[1818]   306 MVTEFIFLGLSDSQELQTFLFMLFFVFYGGIV...LNPIIYTLRNKDMKTAIRRLRKWDAHSSVKF*
[1819]   134 MSESINFSHNLGQLLSPPRCVVMPGMPFPSIR...CHGPGLQGSCYKGETQESVESRVLPGPRHRH*
```

4 Creating New *TranscriptDb* Objects or Packages

The *GenomicFeatures* package provides functions to create *TranscriptDb* objects based on data downloaded from UCSC Genome Bioinformatics or BioMart. The following subsections demonstrate the use of these functions. There is also support for creating *TranscriptDb* objects from custom data sources using `makeTranscriptDb`; see the help page for this function for details.

4.1 Using `makeTranscriptDbFromUCSC`

The function `makeTranscriptDbFromUCSC` downloads UCSC Genome Bioinformatics transcript tables (e.g. `"knownGene"`, `"refGene"`, `"ensGene"`) for a genome build (e.g. `"mm9"`, `"hg19"`). Use the `supportedUCSCtables` utility function to get the list of supported tables.

```
> supportedUCSCtables()[1:4, ]

                                  track        subtrack
knownGene                   UCSC Genes            <NA>
knownGeneOld3           Old UCSC Genes            <NA>
wgEncodeGencodeManualV3  Gencode Genes Genecode Manual
wgEncodeGencodeAutoV3    Gencode Genes   Genecode Auto
```

```
> mm9KG <- makeTranscriptDbFromUCSC(genome = "mm9", tablename = "knownGene")
```

The function `makeTranscriptDbFromUCSC` also takes an important argument called `circ_seqs` to label which chromosomes are circular. The argument is a character vector of strings that correspond to the circular chromosomes (as labeled by the source). To discover what the source calls their chromosomes, use the `getChromInfoFromUCSC` function to list them. By default, there is a supplied character vector that will attempt to label all the mitochondrial chromosomes as circular by matching to them. This is the `DEFAULT_CIRC_SEQS` vector. It contains strings that usually correspond to mitochondrial chromosomes. Once the database has been generated with

the circular chromosomes tagged in this way, all subsequent analysis of these chromosomes will be able to consider their circularity for analysis. So it is important for the user to make sure that they pass in the correct strings to the `circ_seqs` argument to ensure that the correct sequences are tagged as circular by the database.

```
> head(getChromInfoFromUCSC("hg19"))

  chrom    length
1  chr1 249250621
2  chr2 243199373
3  chr3 198022430
4  chr4 191154276
5  chr5 180915260
6  chr6 171115067
```

4.2 Using `makeTranscriptDbFromBiomart`

Retrieve data from BioMart by specifying the mart and the data set to the `makeTranscriptDbFromBiomart` function (not all BioMart data sets are currently supported):

```
> mmusculusEnsembl <-
+    makeTranscriptDbFromBiomart(biomart = "ensembl",
+                                dataset = "mmusculus_gene_ensembl")
```

As with the `makeTranscriptDbFromUCSC` function, the `makeTranscriptDbFromBiomart` function also has a `circ_seqs` argument that will default to using the contents of the `DEFAULT_CIRC_SEQS` vector. And just like those UCSC sources, there is also a helper function called `getChromInfoFromBiomart` that can show what the different chromosomes are called for a given source.

Using the `makeTranscriptDbFromBiomart` `makeTranscriptDbFromUCSC` functions can take a while and may also require some bandwidth as these methods have to download and then assemble a database from their respective sources. It is not expected that most users will want to do this step every time. Instead, we suggest that you save your annotation objects and label them with an appropriate time stamp so as to facilitate reproducible research.

4.3 Using `makeTranscriptDbFromGFF`

You can also extract transcript information from either GFF3 or GTF files by using the `makeTranscriptDbFromGFF` function. Usage is similar to `makeTranscriptDbFromBiomart` and `makeTranscriptDbFromUCSC`.

4.4 Saving and Loading a *TranscriptDb* Object

Once a *TranscriptDb* object has been created, it can be saved to avoid the time and bandwidth costs of recreating it and to make it possible to reproduce results with identical genomic feature data at a later date. Since *TranscriptDb* objects are backed by a SQLite database, the save format is a SQLite database file (which could be accessed from programs other than Rif desired). Note that it is not possible to serialize a *TranscriptDb* object using R's `save` function.

```
> saveDb(mm9KG, file="fileName.sqlite")
```

And as was mentioned earlier, a saved *TranscriptDb* object can be initialized from a .sqlite file by simply using `loadDb`.

```
> mm9KG <- loadDb("fileName.sqlite")
```

4.5 Using `makeTxDbPackageFromUCSC` and `makeTxDbPackageFromBiomart`

It is often much more convenient to just make an annotation package out of your annotations. If you are finding that this is the case, then you should consider the convenience functions: `makeTxDbPackageFromUCSC` and `makeTxDbPackageFromBiomart`. These functions are similar to `makeTranscriptDbFromUCSC` and `makeTranscriptDbFromBiomart` except that they will take the extra step of actually wrapping the database up into an annotation package for you. This package can then be installed and used as of the standard TranscriptDb packages found on in the Bioconductor repository.

5 Session Information

```
R version 3.0.0 (2013-04-03)
Platform: x86_64-unknown-linux-gnu (64-bit)

locale:
```

```
 [1] LC_CTYPE=en_US.UTF-8       LC_NUMERIC=C
 [3] LC_TIME=en_US.UTF-8        LC_COLLATE=C
 [5] LC_MONETARY=en_US.UTF-8    LC_MESSAGES=en_US.UTF-8
 [7] LC_PAPER=C                 LC_NAME=C
 [9] LC_ADDRESS=C               LC_TELEPHONE=C
[11] LC_MEASUREMENT=en_US.UTF-8 LC_IDENTIFICATION=C

attached base packages:
[1] parallel  stats     graphics  grDevices utils     datasets  methods
[8] base

other attached packages:
 [1] BSgenome.Hsapiens.UCSC.hg19_1.3.19
 [2] BSgenome_1.28.0
 [3] Biostrings_2.28.0
 [4] TxDb.Hsapiens.UCSC.hg19.knownGene_2.9.0
 [5] GenomicFeatures_1.12.1
 [6] AnnotationDbi_1.22.3
 [7] Biobase_2.20.0
 [8] GenomicRanges_1.12.2
 [9] IRanges_1.18.0
[10] BiocGenerics_0.6.0

loaded via a namespace (and not attached):
 [1] DBI_0.2-6          RCurl_1.95-4.1     RSQLite_0.11.3     Rsamtools_1.12.2
 [5] XML_3.96-1.1       biomaRt_2.16.0     bitops_1.0-5       rtracklayer_1.20.1
 [9] stats4_3.0.0       tools_3.0.0        zlibbioc_1.6.0
```
